# Dynamic chromatin regulatory programs during embryogenesis of hexaploid wheat

**DOI:** 10.1186/s13059-022-02844-2

**Published:** 2023-01-13

**Authors:** Long Zhao, Yiman Yang, Jinchao Chen, Xuelei Lin, Hao Zhang, Hao Wang, Hongzhe Wang, Xiaomin Bie, Jiafu Jiang, Xiaoqi Feng, Xiangdong Fu, Xiansheng Zhang, Zhuo Du, Jun Xiao

**Affiliations:** 1 Key Laboratory of Plant Cell and Chromosome Engineering, Institute of Genetics and Developmental Biology, Chinese Academy of Sciences, Beijing, 100101 China; 2 University of Chinese Academy of Sciences, Beijing, 100049 China; 3 Nanjing Agricultural University, Nanjing, Jiangsu China; 4 Shandong Agricultural University, Tai’an, Shandong, China; 5 John Innes Centre, Colney Lane, Norwich, NR4 7UH UK; 6 State Key Laboratory of Molecular Developmental Biology, Institute of Genetics and Developmental Biology, Chinese Academy of Sciences, Beijing, 100101 China; 7 CAS-JIC Centre of Excellence for Plant and Microbial Science (CEPAMS), Institute of Genetics and Developmental Biology, Chinese Academy of Sciences, Beijing, 100101 China

## Abstract

**Background:**

Plant and animal embryogenesis have conserved and distinct features. Cell fate transitions occur during embryogenesis in both plants and animals. The epigenomic processes regulating plant embryogenesis remain largely elusive.

**Results:**

Here, we elucidate chromatin and transcriptomic dynamics during embryogenesis of the most cultivated crop, hexaploid wheat. Time-series analysis reveals stage-specific and proximal–distal distinct chromatin accessibility and dynamics concordant with transcriptome changes. Following fertilization, the remodeling kinetics of H3K4me3, H3K27ac, and H3K27me3 differ from that in mammals, highlighting considerable species-specific epigenomic dynamics during zygotic genome activation. Polycomb repressive complex 2 (PRC2)-mediated H3K27me3 deposition is important for embryo establishment. Later H3K27ac, H3K27me3, and chromatin accessibility undergo dramatic remodeling to establish a permissive chromatin environment facilitating the access of transcription factors to *cis*-elements for fate patterning. Embryonic maturation is characterized by increasing H3K27me3 and decreasing chromatin accessibility, which likely participates in restricting totipotency while preventing extensive organogenesis. Finally, epigenomic signatures are correlated with biased expression among homeolog triads and divergent expression after polyploidization, revealing an epigenomic contributor to subgenome diversification in an allohexaploid genome.

**Conclusions:**

Collectively, we present an invaluable resource for comparative and mechanistic analysis of the epigenomic regulation of crop embryogenesis.

**Supplementary Information:**

The online version contains supplementary material available at 10.1186/s13059-022-02844-2.

## Introduction

Embryogenesis, during which the fusion of parental gametes following fertilization generates an entirely new organism, represents the beginning of development and ensures the life cycle for plants and animals [1–4]. Advance in low-input genome-wide chromatin analysis technologies has boosted time-series epigenomic analyses during early embryogenesis in mammals [5, 6]. These pioneering studies have revealed a critical role of epigenomic remodeling in initiating embryogenesis and characterized both conserved and species-specific patterns of epigenomic remodeling [3–5].

Epigenomic dynamics accompany a series of developmental events during mammalian early embryogenesis, including maternal-to-zygotic transition (MZT), zygotic genome activation (ZGA), lineage specification and fate determination, and tissue fate differentiation [4, 7, 8]. During the above processes, histone modifications undergo widespread remodeling, exhibiting both conserved and species-specific patterns [4, 9]. In mice, paternal tri-methylation of histone H3 lysine 4 (H3K4me3), a hallmark of transcriptional activation, is rapidly removed after fertilization but re-established during major ZGA [10]. By contrast, noncanonical H3K4me3 (ncH3K4me3) covering broad domains presents at both promoter and distal regions until the major ZGA stage [11, 12]. In humans, H3K4me3 largely exhibits a canonical pattern, with sharp peaks at promoters [8]. Strong H3K4me3 could still be found at pre-ZGA four-cell stage, with more than half (~ 53%) retained during ZGA, while other loci lose H3K4me3 and remain inactive upon ZGA [8]. Tri-methylation of histone H3 lysine 27 (H3K27me3), a hallmark of transcriptional repression, is erased at promoter regions in a parental-allelic differential manner during early embryogenesis in mice [4, 10]. In humans, global erasure of H3K27me3 also occurs during ZGA in a synchronized manner on both parental genomes [8]. Chromatin accessibility also undergoes pervasive reorganization that is critical for converting the terminally differentiated gametes into a totipotent state [13]. Accessible chromatin is progressively established during early embryogenesis and exhibits a significant increase in 8-cell embryos in mice, correlated with gene activation [14, 15].

In comparison, our knowledge of the epigenomic dynamics, including histone modifications, histone variant, chromatin accessibility, and their potential implications in developmental regulation, such as MZT, lineage specification, fate transition, and embryonic maturation, remains largely unknown in plants, especially for crops with complex genomic structure [16]. Comparisons of embryogenesis between plants and animals highlight both conservation and difference. Plants and animals share a general cell differentiation strategy, in which cell fate specification accompanies embryonic cell divisions. In addition, both transcriptional programs go through the same hourglass model where a conserved phylotypic period during mid-embryogenesis punctuates divergent early- and late-stage between species within a phylum, whereas an inverse hourglass model where transcriptomes during early- and late-embryogenesis between phyla are more comparable than the mid-stage [17–21]. Nevertheless, substantial differences in embryogenesis between the two kingdoms have been widely recognized, such as maternal dominance in animals versus the equal contribution of both parents to early embryogenesis in plants. Moreover, complete versus partial organogenesis, characterized by dormancy or not, has been observed in plants and animals [1, 22, 23]. Thus, elucidating epigenomic landscapes and dynamics that orchestrate plant embryogenesis will provide insights into the epigenomic basis of plant development and facilitate the characterization of specific patterns of epigenomic dynamics in plants.

As an important crop that provides 20% of the calories and protein daily consumed by humans, wheat embryogenesis is of particular interest for elucidating the genomic and epigenomic regulation of the ontogenetic of polyploidy. Hexaploid wheat (AABBDD) results from two times hybridization and polyploidization events of three putative diploid wild grass progenitor species [24, 25]. A recent study has mapped the transcriptional landscape of polyploidy wheat and diploid ancestors during embryogenesis, revealing the evolutionary divergence of gene expression and contribution of the A, B, and D subgenomes to grain development [20]. However, the epigenomic landscapes and their contributions to transcriptional regulatory networks and cell fate transitions remain to be elucidated. To fill this knowledge gap, we mapped the transcriptomic and epigenomic landscapes, including the genome-wide profiles of seven types of histone modifications, H2A.Z, a histone variant, and chromatin accessibility, in eight characteristic developmental stages of wheat embryogenesis, generating an unprecedented resource for studying the epigenomic basis of a hexaploid genome. From the data, we revealed species-specific chromatin remodeling programs in early embryogenesis and provided genetic evidence for PRC2 mediated H3K27me3 function in establishment of embryo, as well as predicted transcriptional circuitry during embryonic patterning. Moreover, we provided evidence explaining the epigenomic contributions to the evolutionary divergence among wheat sub-genomes and the developmental phase-specific transcriptional divergence during wheat embryogenesis.

## Result

### Charting epigenomic landscapes during wheat embryogenesis

We applied CUT&Tag (Cleavage Under Targets and Tagmentation) and ATAC-seq (Assay for Transposase Accessible Chromatin with high-throughput sequencing) to map the histone modification and chromatin accessibility during plant embryogenesis [26, 27]. A pilot run validated the effectiveness of the above methods. First, CUT&Tag results showed high reproducibility between experimental replicates (Pearson correlation coefficient *R* > 0.94) and high correlation with published chromatin immunoprecipitation (ChIP)-seq results (*R* > 0.8) but also high repeatability (*R* > 0.94 between two experimental replicates). Second, we validated that the methods were sensitive to lower nuclei input. Robust CUT&Tag results were obtained using 1000 nuclei at a sequencing depth of ~ 1/3–1/20 of ChIP-seq experiments (Additional file 1: Fig. S1a-c). ATAC-seq from about 5000 nuclei showed good signal-to-noise ratio, reasonable fragment size and genomic distribution, and fully met the demand of transcription factor (TF) footprint calling (Additional file 1: Fig. S1d-h).

**Fig. 1:**
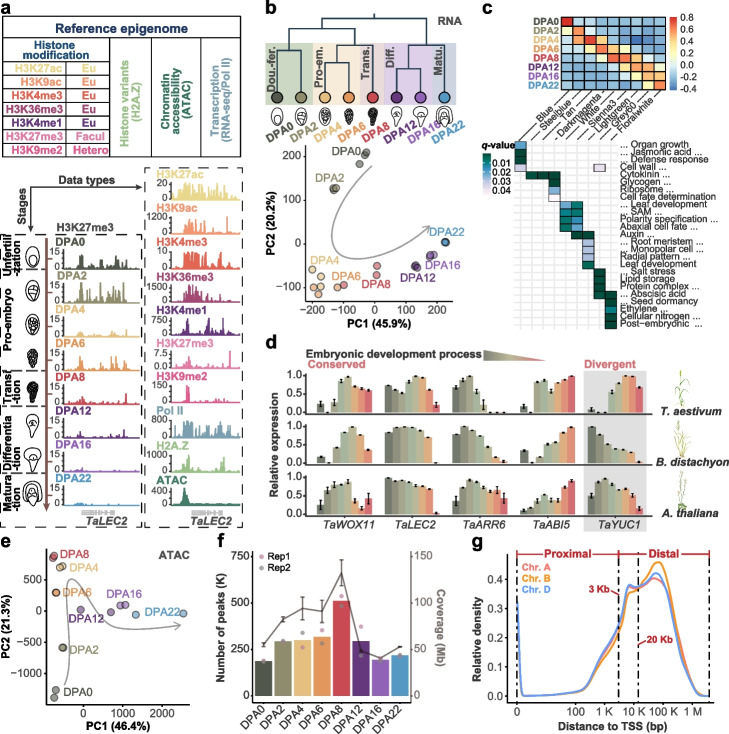
Charting chromatin landscapes of wheat embryogenesis. **a** Experimental design and major axes of the data series: data types and sampling developmental stages. The embryo sac was sampled for DPA0-DPA4, while the isolated embryo was used for DPA6-DPA22. DPA, days post anthesis. **b** Cluster dendrogram and PCA of transcriptome showing five distinct development stages: around double fertilization (DPA 0/2), pro-embryo (DPA 4/6), transition (DPA 8), differentiation (DPA 12/16), and maturation (DPA 22). Three biological replicates were sequenced for each developmental stage. **c** Representative modules from WGCNA cluster and enrichment of corresponding GO. **d** The conserved and diversified expression pattern of representative genes between monocot (T. aestivum and B. distachyon) and dicot (A. thaliana) during embryogenesis. **e**–**g** Dynamic pattern of genome-wide chromatin accessibility measured by ATAC-seq during embryogenesis. PCA analysis (**e**), peak numbers and peak coverage dynamics (**f**), and peak distribution in different sub-genomes (**g**). Two biological replicates were used for data analysis

Having the techniques established, we generated a ‘reference epigenome’ of wheat embryogenesis across eight characteristic stages (DPA 0, 2, 4, 6, 8, 12, 16, 22, days post anthesis). Embryo sacs were used for early embryogenesis (DPA0-4), while dissected embryos were sampled for later stages (DPA6-22). The epigenome includes seven histone modifications (H3K4me1, H3K4me3, H3K9ac, H3K9me2, H3K27ac, H3K27me3, and H3K36me3), occupancy of histone variant H2A.Z and RNA polymerase II, and chromatin accessibility, as well as transcriptomes (Fig. 1a). Epigenomic and transcriptome data contained two or three biological replicates, respectively. Globally, chromatin marks exhibited highly characteristic distribution patterns correlated with gene transcription (Additional file 1: Fig. S2a, b).

Plant embryogenesis generally divides into three stages: early embryogenesis, during which a totipotent zygote is formed following fertilization; mid embryogenesis, following which the major cell lineages and body pattern are built; and late embryogenesis, during which nutrition is accumulated in the mature embryo followed by seed dormancy [1, 2]. Unsupervised principle component analysis (PCA) and hierarchical clustering of RNA-seq and ATAC-seq revealed a continuous trajectory (Fig. 1b, e), whereas histone modifications showed a punctured stage-specific transition pattern (Additional file 1: Fig. S2c). GO enrichment of temporally correlated gene modules revealed that DNA replication was overrepresented during early embryogenesis, organ/tissue specification genes were enriched during mid-embryogenesis, and nutrient accumulation and dormancy-related genes were expressed more frequently during late embryogenesis (Fig. 1c). Known factors involved in embryo formation, such as *WOX11*, *LEC2*, *ARR6*, and *ABI5*, but not other genes, such as *YUC1*, showed conserved embryonic expression patterns between wheat, *Brachypodium*, and *Arabidopsis* (Fig. 1d). Such patterns indicate the evolutionary conservation and divergence of monocots and dicots embryogenesis [20, 21, 28]. Accessible chromatin was progressively established until DPA8, the transition stage for fate patterning, followed by gradually decreasing afterward (Fig. 1f). Notably, the majority of ATAC-seq peaks (an indicator of accessible chromatin) were located more than 3 Kb (20 Kb in specific cases) away from the transcriptional start site (TSS) of wheat genes (Fig. 1g and Additional file 1: Fig. S1f); the distance was significantly distant than plants with a small genome [26, 29]. Interestingly, the ATAC-seq peak-TSS distance and proportion of distal ATAC-seq peaks increased as a function of genome size in plant species. For example, the median distance was 129 bp in *Arabidopsis* with a genome of 125 Mb, whereas the value increased to 4620 bp in barley with a ~ 5 Gb genome (Additional file 1: Fig. S2d) [29]. Consistently, the largest B subgenome of hexaploid wheat has more distal ATAC-seq peaks than the A and D subgenomes (Fig. 1g) [25].

Taken together, we have generated a comprehensive set of epigenomic and transcriptomic datasets, representing a “reference epigenome” of wheat embryogenesis. The concordant changes of histone modification and chromatin accessibility with gene transcription facilitate elucidating the epigenomic basis of diverse regulatory events in wheat embryogenesis.

### Distinct proximal and distal chromatin accessibility dynamics

**Fig. 2:**
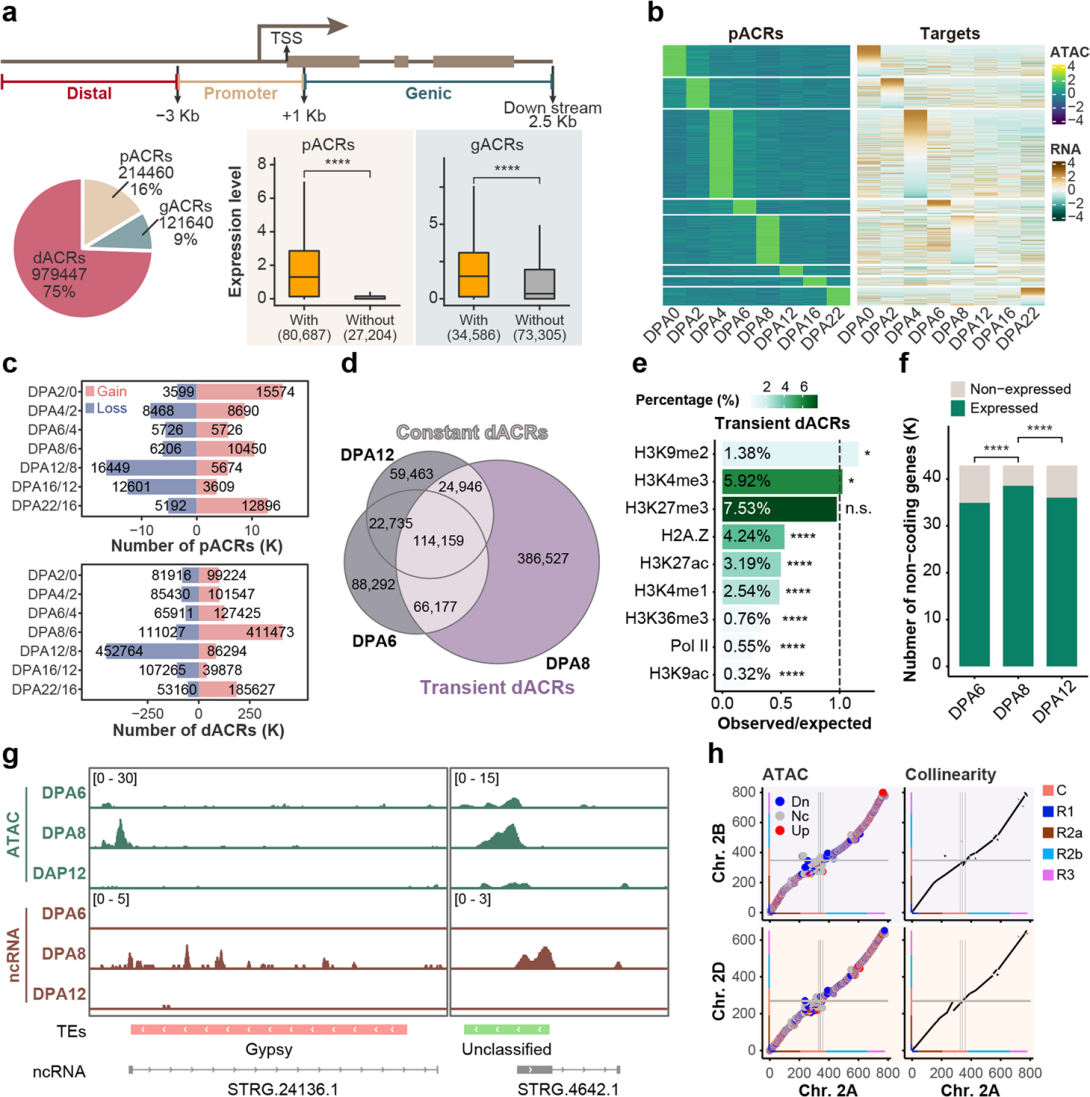
Distinct feature of different ACRs in transcriptional regulation in wheat. **a** Subdividing ACRs based on distribution pattern around genes, and correlation between /gACRs and genes expression. **b** Heatmap showing stage-specific pACRs and corresponding genes expression pattern. **c** Distinct gain and loss of pACRs and dACRs between adjacent embryonic developmental stages. d Venn diagram showing overlapping dACRs among DPA6, 8, and 12. **e** Histone modifications enrichment at transient dACRs. Overlap between transient dACRs peaks and different epigenetics modification peaks was calculated by bedtools and peaks with 1 bp overlap were considered as overlap. The observed/expected ratio was calculated between the observed overlap number divided randomly selected background overlap number. The randomly selected background was generated from genomic intervals that with any epigenetics modification and located in intergenic regions. For more details, see Method. **f** The number of expressed non-coding RNA during DPA6-12. **g** IGV showing the transient dACRs was correlated with non-coding RNA-specific expression at DPA8, which overlapped with TEs. **h** Comparisons of chromatin accessibility of collinear regions among subgenomes, chromosome 2B compared to 2A (up-left) and chromosome 2D with 2A (down-left). Collinear regions were identified based on sequence conservation (right). Color bars on x and y axis indicate the chromosome segments defined by IWGSC Refseq v1.0. Grey lines indicate the centromere for individual chromosomes. Mann–Whitney U test (two-sided) was used for a. Fisher exact test was used for e and f. *p <  = 0.05; **p <  = 0.01; ***p <  = 0.001; ****p <  = 0.0001

Accessible chromatin exposes regulatory DNA sequences to transcription factors, which provides an effective way to elucidate transcriptional regulation [30]. In total, we identified 1,315,547 accessible chromatin regions (ACRs) from ATAC-seq, which were further categorized into gene body (g), promoter (p), and distal (d) ACRs based on the location relative to genes (Fig. 2a). As expected, both pACRs and gACRs were significantly associated with transcriptional activation (Fig. 2a, 2b, and Additional file 2: Dataset S1). Interestingly, the gain and loss of pACRs and dACRs varied during embryogenesis (Fig. 2c and Additional file 1: Fig. S3a). Many genes gained pACRs after fertilization at DPA2 but dramatically lost them during late embryogenesis at DPA12 and DPA16 (Fig. 2c). Significantly, dACRs, accounting for about 75% of total ACRs, exhibited a sharp “burst” at DPA8 and then quickly declined at DPA12 (Fig. 2c). This transient increase in chromatin accessibility is specific to the dACRs as both peak number and the signal intensity of ACRs at the proximal region were comparable among DPA6, DPA8, and DPA12 (Additional file 1: Fig. S3b, S3c).

We further analyzed the feature of ‘transient’ dACRs at DPA8 as compared to “constant” dACRs that presented at DPA8 and either of DPA6 or DPA12 (Fig. 2d, e and Additional file 1: Fig. S3d, S3e). The constant dACR region is generally enriched for active chromatin states, such as Pol II and H3K27ac marked regions, while depleted with TEs and heterochromatin-related histone modifications, such as H3K9me2 (Additional file 1: Fig. S3d, S3e). By contrast, transient dACRs harbored a higher proportion of TEs (~ 75%) compared with constant dACRs (~ 40%) and were relatively enriched for H3K9me2 (Fig. 2e, Additional file 1: Fig. S3d). Consistent with gained dACRs, more non-coding RNA (ncRNA) were detected at DPA8 as compared to DPA6 and DPA12 (Fig. 2f, Additional file 1: Fig. S3f). Of note, more than 74% of transient dACRs marked ncRNA (*n* = 6,412) are transcribed from TEs loci (Fig. 2g). Thus, the burst of certain dACR at DPA8 may be correlated with the transient expression of TEs during embryogenesis. Besides the temporal dynamics, subgenomes of hexaploid wheat exhibited considerable differences in chromatin accessibility, especially at the centromere and telomere, in addition to the low-collinear chromosome regions (Fig. 2h and Additional file 1: Fig. S3g).

In summary, proximal and distal chromatin accessibility undergoes distinct reprogramming during embryogenesis. Interestingly, a large proportion of distal regulation and asymmetry of chromatin accessibility among subgenomes exhibit uniqueness for the large hexaploid genome.

### Species-specific chromatin remodeling during embryogenesis

**Fig. 3:**
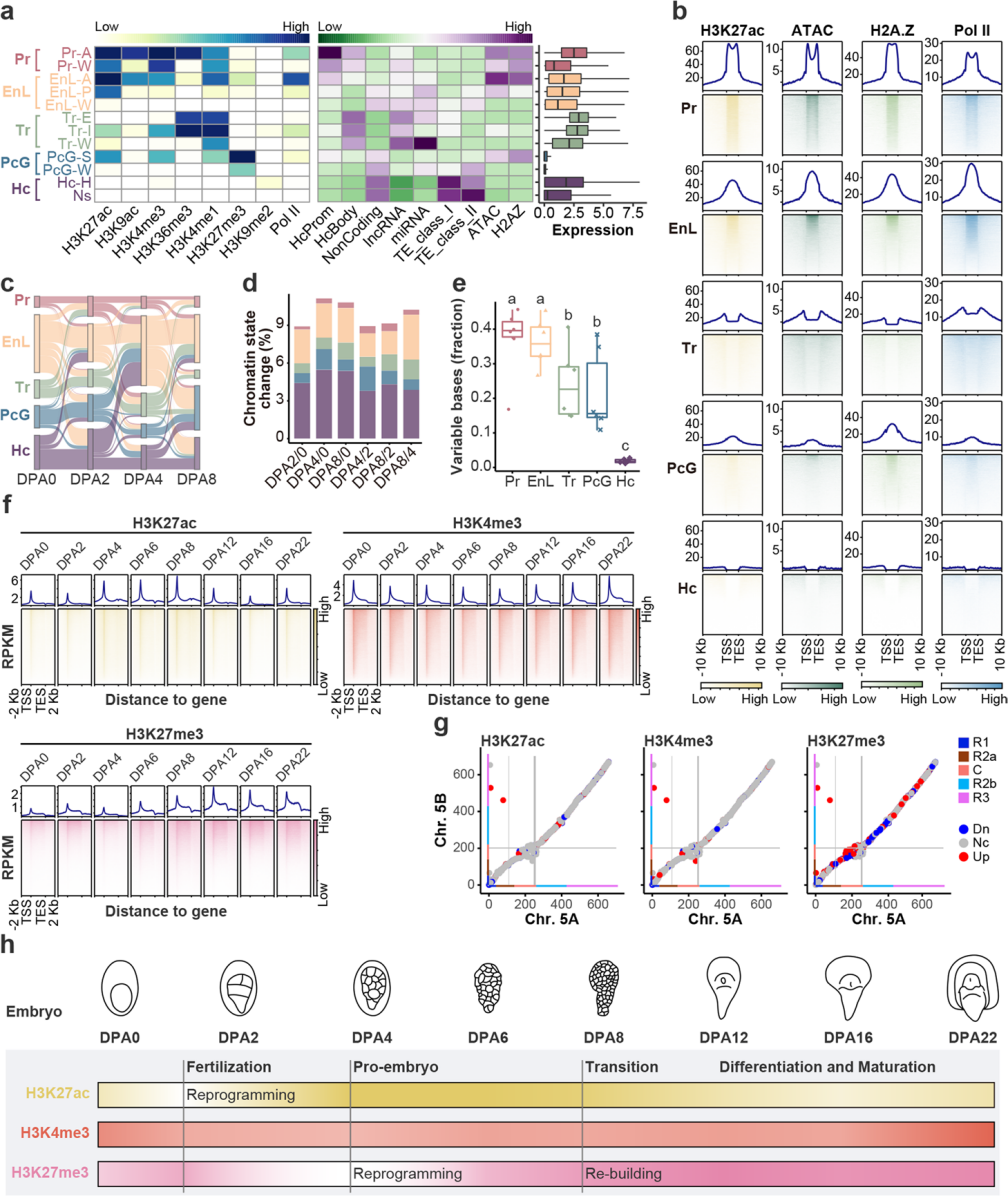
Reprogramming of histone modifications during wheat embryogenesis. **a** Emission probabilities for histone modifications in 12 ChromHMM states, which could be mainly categorized into five groups: Pr (promoter), EnL (enhancer-like), Tr (transcription), PcG (polycomb group), and Hc (heterochromatin) state. The abbreviation following dash: A, active; W, weak; P, poised; S, strong; I, initiation; E, elongation; H, H3K9me2-associated; Ns, No signal. **b** Heatmap showing the histone modification level at different chromatin states. RPKM was used for data normalization. **c** Chromatin states transition among different developmental stages. **d** Percentage of changed chromatin state coverage to the total genome size among different embryonic stages. Colors filled represent the five major chromatin state groups as shown in **a**. **e** Dynamic of five major chromatin states during different embryonic stages. Fisher’s Least Significant Difference (LSD) was used for significance calling. **f** Histone modification dynamic in promoter and genic regions during wheat embryogenesis. g Comparisons of histone modifications H3K27ac, H3K4me3, H3K27me3, and H3K9me3 of collinear regions between Chr. 5A and Chr. 5B. Downregulated (Dn) and upregulated (Up) loci were defined by the log2 (fold change Chr. 5B/ Chr. 5A) values bigger and lower than 1 and − 1, respectively. Nc indicates no change. Color bars and grey lines indicate the chromosome segments and centromere regions as those in Fig. 2d. h Schematic represents the chromatin state dynamic during wheat embryogenesis. The dark and light colors represent the strength and weakness of the signal, respectively

To infer chromatin states, we integrated profiles of eight histone modifications and Pol II occupancy from four embryonic developmental stages (DPA 0, 2, 4, 8) using ChromHMM [31, 32]. Totally, 12 chromatin states were categorized, which were further grouped into five major functional classes: promoter (Pr), enhancer-like (EnL), transcriptional (Tr), polycomb group (PcG), and heterochromatin (Hc) (Fig. 3a). Promoter and enhancer-like classes were enriched for H3K27ac, higher chromatin accessibility, histone variant H2A.Z, and Pol II occupancy (Fig. 3a, 3b, Additional file 1: Fig. S4a). As expected, chromatin states were dramatically altered across embryo stages (Fig. 3c), around 10% of the genome changed chromatin state between adjacent developmental stages (Fig. 3d), with the promoter and enhancer-like class varying the most (Fig. 3e).

In mice and humans, H3K4me3 and H3K27me3 are extensively reprogrammed immediately after fertilization, which is pivotal for early embryogenesis [4, 9]. Thus, we investigated the dynamics of the above histone modifications during wheat embryonic development, separating proximal and distal regions as those histone modifications exhibit large distributions in the intergenic regions (Additional file 1: Fig. S2a). Genome-wide H3K4me3 exhibited a moderate change in both proximal and distal regions, as well as H2A.Z (Fig. 3f and Additional file 1: Fig. S4b, S4c). Instead, H3K27ac exhibited a sharp decline at the pro-embryo stage, then restored and maintained high until mid-embryo, followed by gradually decreasing at late and mature embryo stages (Fig. 3f and Additional file 1: Fig. S4b). For H3K27me3, a sharp erasure occurred at the pro-embryo stage, then gradually restored and kept high at late and maturation stages (Fig. 3f and Additional file 1: Fig. S4b). Interestingly, subgenomes of hexaploid wheat exhibited a different abundance of histone modifications even in collinear regions (Fig. 3g and Additional file 1: Fig. S4d, S4e). H3K27me3 exhibited higher variation among sub-genomes than active histone marks such as H3K27ac, H3K4me3, and histone variant H2A.Z (Fig. 3g and Additional file 1: Fig. S4d, S4e).

Together, we revealed species-specific dynamic patterns of histone modification reprogramming during early embryogenesis in wheat (Fig. 3f, h, and Fig S4b, S4c). Different subgenomes of polyploidy wheat have varied histone modification, especially for repressive histone modification H3K27me3 (Fig. 3g, and Fig S4d, S4e).

### Reprogramming of H3K27ac, H3K27me3, and chromatin accessibility shapes gene expression dynamics in early embryogenesis

**Fig. 4:**
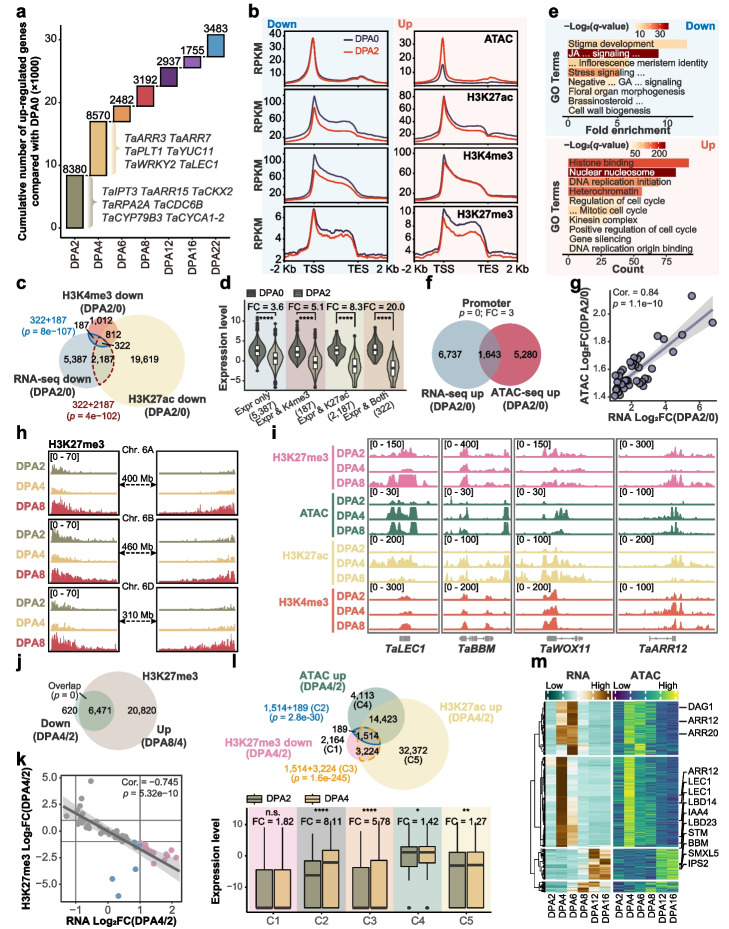
H3K27ac and H3K27me3 reprogramming and chromatin accessibility change shape early embryogenesis. **a** Waterfall plots showing the number of genes first upregulated at different stages compared with DPA0. Representative genes were listed. **b** Chromatin accessibility and histone modification profiles of downregulated (left) and upregulated (right) genes. **c** Overlap between downregulated genes and corresponding active histone modification marks decreasing. **d** Expression levels of different gene sets generated from **c**. **e** GO enrichment for 2696 downregulated genes whose active histone modification marks decreased (**c**) (top) and 1643 upregulated genes which gained pACRs (**f**) (bottom). **f** Overlap between up-regulated genes and corresponding chromatin accessibility gains. **g** Correlation between gained pACRs and up-regulated genes based on 1643 genes overlapped. Genes were ranked by RNA-seq fold change and separated into 50 bins. **h** Dynamic of H3K27me3 modification in Chr. 6 during embryogenesis, with resetting at DPA4 and regaining at DAP8. **i** IGV showing histone modifications and chromatin accessibility changes on embryo developmental essential genes. **j** Overlap between genes with H3K27me3 decreasing at DPA4 and increasing at DPA8. **k** Correlation between H3K27me3 decreasing and target genes’ activation from DAP4 to DPA2. Genes were ranked by RNA-seq fold change and separated into 50 bins. H3K27me3 loss with mRNA level increased genes are shown in red color, while loss of H3K27me3 without mRNA changed is shown in blue color, while gray color indicates no change of H3K27me3. **l** Overlap among genes with downregulated H3K27me3 and up-regulated ATAC-seq and H3K27ac modification (top) and altered expression levels of different gene sets (bottom). **m** Synchronous pattern between the gain of chromatin accessibility and elevation of gene expression on the basis of H3K27me3 decreasing at DPA4. Several developmental essential genes were highlighted. Fisher exact test was used for significance calling for **c**, **f**, **j**, and **l**, Pearson test was used for **g**. Mann–Whitney U test (two-sided) was used for difference comparison in **d** and **l**, * *p* <  = 0.05; **p <  = 0.01; ***p <  = 0.001; ****p <  = 0.0001

Upon fertilization, the maternal program is silenced, followed by zygotic gene activation, during which epigenetic reconfiguration occurs in both animals and higher plants [9, 23, 34]. During wheat embryogenesis, gene activation was mainly observed at DPA2 and 4, including cell cycle and cytokine signaling genes associated with zygotic activation (Fig. 4a, Additional file 1: Fig. S5a and Additional file 3: Dataset S2). Furthermore, active histone modification H3K27ac and H3K4me3 suffered an overall lopsided decrease at down-regulated genes, while nearly no change occurred at up-regulated genes (Fig. 4b and Additional file 1: Fig. S5a), which is different from the counterparts in mammals [4, 9]. H3K27me3 decreased at both up-and down-regulated genes, indicating that H3K27me3 may not be the major regulator for the divergent expression of genes. By contrast, chromatin accessibility did not change much at downregulated genes but gained at up-regulated genes (Fig. 4b). Such profiles indicate that loss of active histone modification may contribute to gene silencing, while gain of chromatin accessibility with reduction of H3K27me3 may trigger gene activation. Indeed, attenuation of either or both H3K27ac and H3K4me3 was extensively enriched for down-regulated genes (Fig. 4c, d, Additional file 1: Fig. S5b and Additional file 4: Dataset S3), with remarkable down-regulation for loss of both modifications. H3K27ac reprogramming was more effective compared with H3K4me3 (Fig. 4d). Genes subjected to both decreases in active histone marks and transcriptional levels were enriched for maternally-silenced genes, such as orthologs of floral homeotic gene *AP2*, signaling-related gene *MAPKKK17*, and stigma expressed gene *IAA1* (Fig. 4e, Additional file 1: Fig. S5a, b). Whereas genes gained chromatin accessibility exhibited a significant overlap and correlation with transcriptional up-regulation (Fig. 4f, g, Additional file 1: Fig. S5b and Additional file 5: Dataset S4). These genes were enriched for DNA replication, cell cycle, heterochromatin, etc., which are essential for zygotic initiation (Fig. 4e and Additional file 1: Fig. S5a, b).

After zygotic initiation, we observe a genome-wide drop of H3K27me3 at DPA4 but sharply re-built at DPA8 (Fig. 4h, Additional file 1: Fig. S5c and Additional file 6: Dataset S5). For essential embryonic genes such as *LEC1*, *BBM*, *WOX11*, and *ARR12*, a permissive chromatin environment was observed at pro-embryo (DPA4), with decreased H3K27me3 and increased H3K27ac, and ATAC-seq signal, while a limited change of H3K4me3 from DPA2 (Fig. 4i). About 90% of the H3K27me3 erasure regions at pro-embryo (DPA4) were re-built in the transition stage (DPA8), along with a large amount of de novo gain of H3K27me3 regions (Fig. 4j). The erasure of H3K27me3 was associated with the cytokinin pathway and essential embryonic genes, while the few augment was involved in the auxin-related genes (Fig. 4j and Additional file 1: Fig. S5d, e). In general, H3K27me3 was negatively correlated with transcription (Fig. 4k and Additional file 1: Fig. S2b). However, decreasing H3K27me3 alone could not explain gene activation from DPA2 to DPA4 (Fig. 4k, l). Of note, H3K27me3 reduction was highly overlapped with adding H3K27ac and chromatin accessibility (Fig. 4l). Overlapped genes showed significant activation, with a more dramatic effect by gaining chromatin accessibility (Fig. 4l and Additional file 7: Dataset S6). Furthermore, there were 561 genes whose transcriptional activation exhibited highly synchronous patterns with a gain of chromatin accessibility after DPA4 (Fig. 4m and Additional file 8: Dataset S7). This synchronization likely ensures the proper timing of gene activation. For example, essential gene *LEC1* was first activated followed by pluripotency gene *ARR12* and later differentiation genes *XMXL5* and *IPS2* (Fig. 4m and Additional file 8: Dataset S7). Together, this suggests that resetting H3K27me3 lifts the chromatin layer barrier on embryo patterning genes, which is progressively activated along with the gain of chromatin accessibility.

By contrast, the global H3K27me3 was readily found at DAP6 and fully restored at DAP8 (Fig. 4h, i and Fig S5c). It suggests that the re-gain of H3K27me3 might be important for proper embryo development. In *Arabidopsis*, Polycomb repressive complex 2 (PRC2) is responsible for deposition of H3K27me3; consisting of four subunits including the single-copy gene encoded FERTILIZATION-INDEPENDENT ENDOSPRM (FIE) [35]. In wheat, there are seven genes encoding *FIE* orthologues (Additional file 1: Fig. S6a), with a group of homoeologs (A/B/D triads) highly expressed during embryogenesis (Additional file 1: Fig. S6a). In situ hybridization further confirmed the high expression of *TraesCS7D02G305100* (one of the triads) in embryo at DPA6, while gradually reduced at DPA8 and DPA12 (Additional file 1: Fig. S6b). We further generated mutations of this group of FIE coding triads by genome editing [36, 37]. Various lines with different combinations of mutations of the triads (*fie*_*aabbdd*) were genotyped by sequencing (Additional file 1: Fig. S6c). In heterozygous line *D47* (*fie*_aabbDd), all the triads harbored severe mutation with frameshift, while for homozygous lines (*fie*_aabbdd) *B69* and *C87*, each had a weak mutation for either A or B triads, respectively (Additional file 1: Fig. S6c). Consistently, offspring of *D47* line showed severe embryo development defects at the early DPA6 stage (Additional file 1: Fig. S6d), the homozygous seeds of *D47* were likely arrested (Additional file 1: Fig. S6e), whereas the homozygous line *C89* was fertile but showed delayed embryonic development (Additional file 1: Fig. S6d) and reduced germination rate (Additional file 1: Fig. S6f). In wildtype KN199, the abaxial-adaxial axis of the embryo becomes obvious and the scutellum arises from the abaxial side at DPA8; while the scutellum was still not visible at DPA12 in line *C87* (Fig S6d). Thus, PRC2-mediated H3K27me3 deposition is important for wheat embryo development.

Collectively, reprogramming of histone modification, particularly H3K27ac and H3K27me3, and establishing chromatin accessibility at early embryo development correlates with the proper transition from maternal to zygotic process and re-gain of PRC2 deposition of H3K27me3 is vital for the embryo development.

### Transcription factors mediated regulatory network orchestrates embryonic patterning

**Fig. 5:**
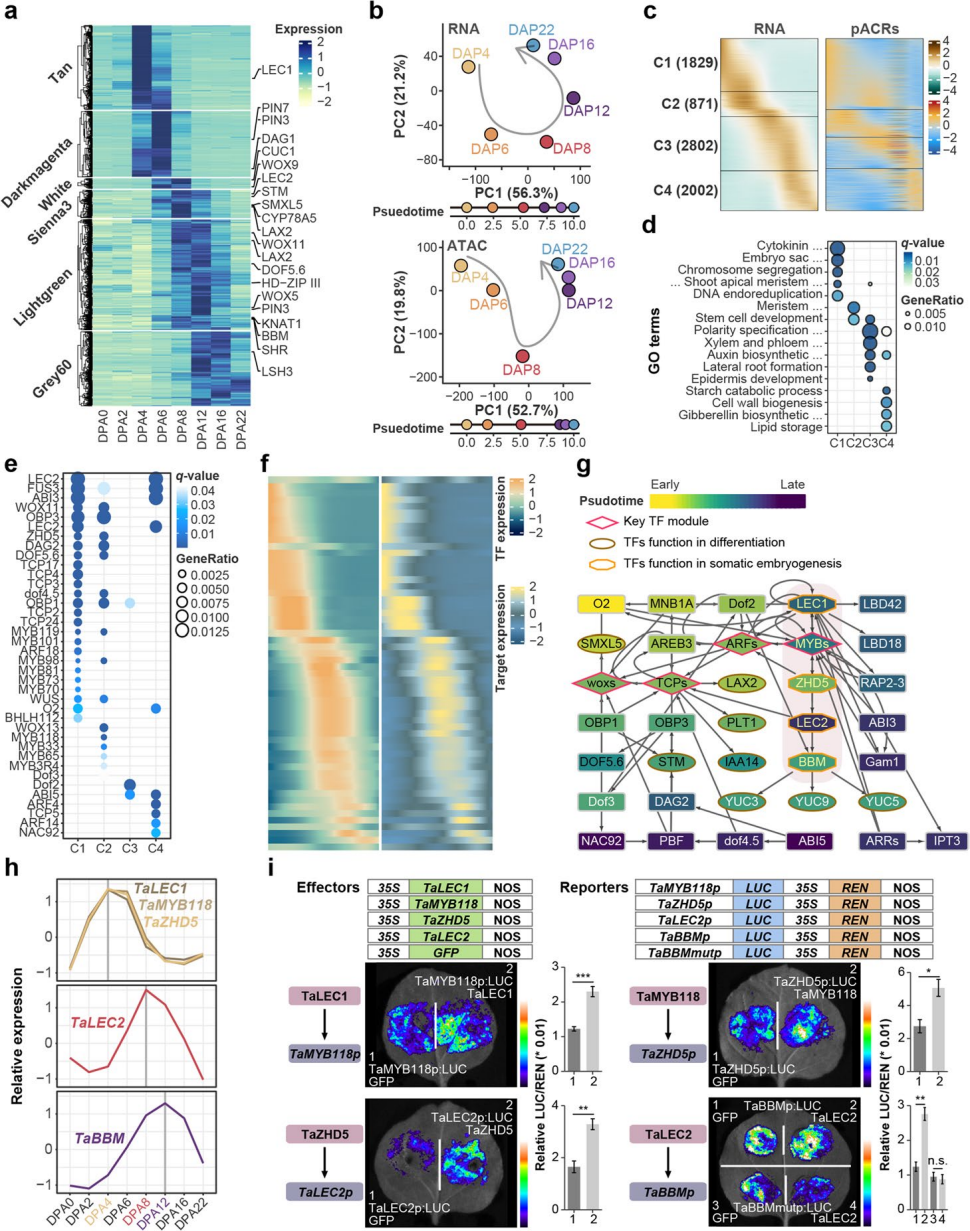
Transcription regulatory network governing embryonic patterning. **a** Expression heatmap of selected key modules genes from Fig. 1c. Genes with ortholog identified to function during embryogenesis in other species were indicated. **b** PCA trajectories of genes expression (top) from (a) and corresponding chromatin accessibility (bottom). Developmental time unit (DTU) values on the right panel were calculated based on the straight distance between two adjacent points and then scaled from 0 to 10. **c** Sorted standardized temporal genes, pACRs and expression profiles. Four clusters were generated based on expression order. **d**, **e** GO and TFs enrichment for four clusters of genes from (**c**). **f** Heatmap showing the synchronous patterns between TFs and target genes. Each row in heatmap represents links between one TF and corresponding genes. **g** GRNs for key TFs participated in embryonic patterning. Genes were roughly ranked by the expression order from top to bottom and from left to right. Colors indicate the expression timing wave during embryo patterning. Shapes indicate the types of TFs. **h** Expression pattern of representative key genes in the GRN network. **i** Luciferase reporter assays validation of transcriptional regulation among representative TF-target pairs. Mutation of B3 domain protein binding sites was introduced in proximal ACR region of TaBBM promoter, indicated as TaBBMmutp. Student’s t test was used for the statistical significance (*p <  = 0.05; ** *p* <  = 0.01; ***p <  = 0.001)

During mid-embryogenesis, the chromatin environment was relatively accessible, marked by high ATAC-seq and H3K27ac signals (Fig. 1f, 3f, h, and Additional file 1: Fig. S3a, S4b). Embryo patterning is gradually formed along with a precisely programmed transcription regulation pyramid [38]. The complex relationship between TFs-target genes and TFs per se could be well exemplified by the continuous transcription trajectories (Fig. 5a, b and Additional file 9: Dataset S8). We calculated the pseudotime of stages covering early- to mature-embryo based on the PCA distance (Fig. 5b). As expected, an excellent association between gene expression and pACR was observed (Fig. 5c), indicating that individual pACRs and TF binding govern the expression of these genes. A comparison of genes expressed in the early time point (clusters 1 and 2) and the latter ones (cluster 3 and 4) revealed a functional diversity, in which the early elevated genes were involved in cell division, whereas the latter ones in cell specification (Fig. 5d). Moreover, motifs of TFs binding sites, whose orthologs can facilitate inducement of cell totipotency in *Arabidopsis*, were enriched in early built ACRs, such as the binding motifs of LEC1, MYB118, WUS, etc. (Fig. 5e). By contrast, the TFs functioning in seed dormancy mainly occupied the later elevated ACRs, such as ABI5 (Fig. 5e). This result demonstrates the evolutionary conservation of several key factors in embryo patterning in monocot and eudicot.

We further dissect the architecture of the gene regulation network. In addition to binding motif specificity, we filtered target genes to TFs by co-expression pattern (Method; Fig. 5f). As a result, 1158 paired TF-cis motif interactions were identified, including 154 target genes, 695 ACRs, and 191 TFs (Fig. 5f). To better understand how TFs are coordinated during embryo pattern formation, we extracted the TF-TF networks (Fig. 5g). We identified four pivotal classes of TFs at the center of the network, TCPs, ARFs, MYBs, and WOXs. Some TFs, such as WOXs and ARFs, were reported to regulate embryo patterning in other species. Several known genes regulating organ and tissue specification in *Arabidopsis* were governed by these TF modules, such as I*AA14*, *LAX2*, *PLT1*, and *SMXL5*. We selected a regulatory module containing LEC1- Myb118- ZHD5- LEC2- BBM for validation. We found *LEC1*, *Myb118*, and *ZHD15* genes were highly expressed in pro-embryo stages, while *LEC2* was activated later, with the peak expression occurring at the transition stage, and *BBM* gradually induced at mid-embryo, indicating a sequential expressed pattern (Fig. 5h). The luciferase activity from the reporter assay further validated the regulatory circuit along LEC1, MYB118, ZHD5, LEC2, and BBM module (Fig. 5i). Interestingly, TaLEC2 could activate *TaBBM* expression via the B3 domain binding motifs within pACR of *TaBBM* (Fig. 5i), which is not reported in model plant *Arabidopsis*.

Thus, a cohesive and sequential wave of TF regulation governs embryo pattern formation during mid-embryogenesis in wheat.

### Chromatin condensation associated with totipotency reduction and organogenesis restriction during embryonic maturation

**Fig. 6:**
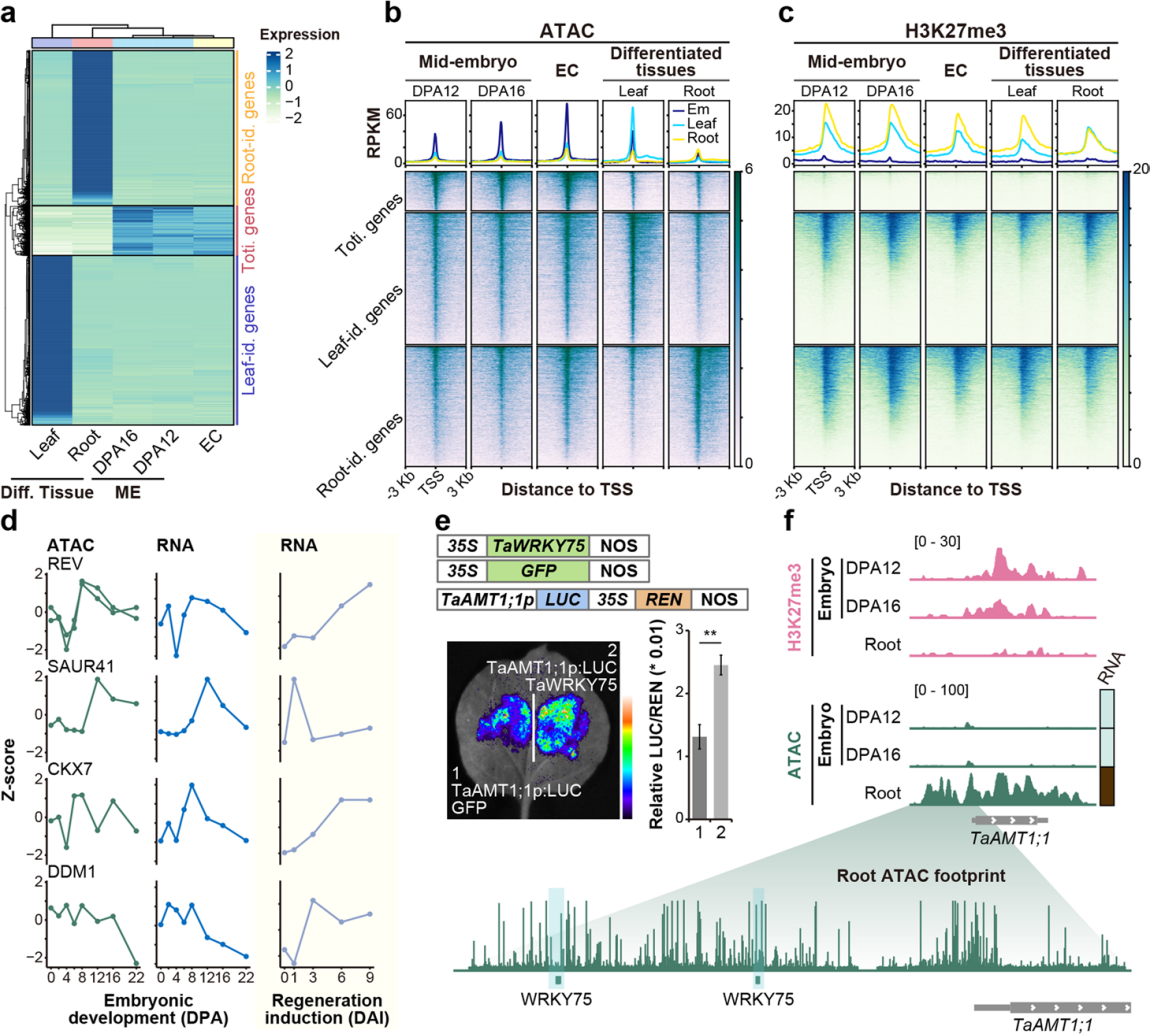
Maturation of embryogenesis in the context of chromatin regulation. **a** Identification of organ identity and totipotent genes based on expression specification across different tissues, ME: mid-embryo, EC: embryonic callus, Leaf-id: leaf identity, Root-id: root identity, Toti. genes: totipotent genes. **b**, **c** ATAC (**b**) and H3K27me3 (**c**) modification on the promoters of different gene sets in a cross five tissues. **d** Chromatin accessibility (left) and expression (mid) of regeneration genes during wheat embryogenesis and their expression during callus induction (right). **e** Luciferase reporter assays show the transcriptional activation capability of TaWRKY75 to TaAMT1;1 in N. benthamiana leaves. Student’s t test was used for the statistical significance (*p <  = 0.05; **p <  = 0.01; ***p <  = 0.001). f H3K27me3 and chromatin accessibility regulated root-identity gene TaAMT1;1 specific expression pattern in root and late embryo (LE). The ATAC footprint tracks showing the binding sites of *TaWRKY75* at the promoter of *TaAMT1;1*

Along with embryo maturation, cells differentiate and gradually lose totipotency (Additional file 1: Fig. S7a), but unlike animals, extensive organogenesis is prohibited in plants [1–4]. We asked how the totipotency capability is attenuated and why the terminal organ is not formed during embryo maturation. First, we identified organ identity genes by their specific expression in terminal organs such as roots and leaves. For comparison, totipotent genes were characterized by high expression in mid-embryo or embryonic callus (Fig. 6a and Additional file 10: Dataset S9). GO enrichment of different groups of genes verified the accuracy of gene identity (Additional file 1: Fig. S7b). Next, we evaluated the chromatin landscape of each gene cluster in different organs. Depletion of H3K27me3 on organ identity genes was observed at the respective organ, whereas obvious ATAC-seq signals were detected at both totipotency and organ identity genes in corresponding organs (Fig. 6b, c, Additional file 1: Fig. S7c, d). Surprisingly, low/no H3K27me3 was enriched in totipotent genes even in differentiated organs (Fig. 6c). In embryonic callus, from which new plant will regenerate, the promoter region of both totipotent genes and organ identity genes were opened with high chromatin accessibility and low H3K27me3 level (Fig. 6b, c). Thus, chromatin accessibility and H3K27me3 coordinately regulate the organ identity gene’s activation potential, while chromatin accessibility but not H3K27me3 mediates repression is indispensable for totipotency gene silencing. Indeed, several regeneration-related factors, such as REV [39], SAUR41 [40], CKX7 [41], and DDM1 [42], were gradually diminished during embryo maturation along with closing chromatin status but activated during the callus induction process (Fig. 6d). Last, we investigated the gene regulatory logic underlying plant organ formation. WRKYs and AtHB TFs footprint were enriched in regulatory regions of root and leaf identity genes, respectively, whereas bHLH and GATA TFs footprint were enriched in both gene sets (Additional file 1: Fig. S7e). Remarkably, nearly half of the identified TFs did not show organ-specific expression but a wide-spectrum pattern, indicating that TF expression per se cannot determine the specificity of organ identity (Additional file 1: Fig. S7f and Additional file 11: Dataset S10). For example, although *WRKY75* was expressed during embryo maturation and it owns the capability to bind and active *AMT1;1*, an ammonium transporter coding gene in the root (Fig. 6e, Additional file 1: Fig. S7f), AMT1;1 was silenced due to the H3K27me3 repression and compaction chromatin environment at the promoter (Fig. 6f).

Taken together, H3K27me3 and chromatin compaction mediated epigenetic regulation associated with the proper embryo maturation process, with reduced totipotency capability but not extensive organogenesis in wheat.

### Epigenomic regulation contributes to subgenome divergence of polyploid genome and stage-specific transcriptional divergence

As wheat is an allohexaploid plant, we further examined the epigenomic contributions to the evolution and polyploidization. We clustered genes based on evolutionary age (Additional file 1: Fig. S8a) and found that most of the genus *Triticum* specific genes, especially genes from *A. tauschii* (DD), did not participate in embryonic development (Additional file 1: Fig. S8a, S8b). For the homeolog triads present in all three subgenomes, chromatin accessibility was associated with the bias expression pattern (Additional file 1: Fig. S9) in addition to histone modifications as reported [43]. As for the bias-expressed homoeologs, suppressed triads were generally more than dominant triads, and D-subgenome suppressed triads were relatively less than A or B-subgenome suppressed. More balanced triads were expressed in the middle period than in early and late embryogenesis (Additional file 1: Fig. S8c). Such observation was generally consistent with the previous report but different in detail [20].

**Fig. 7:**
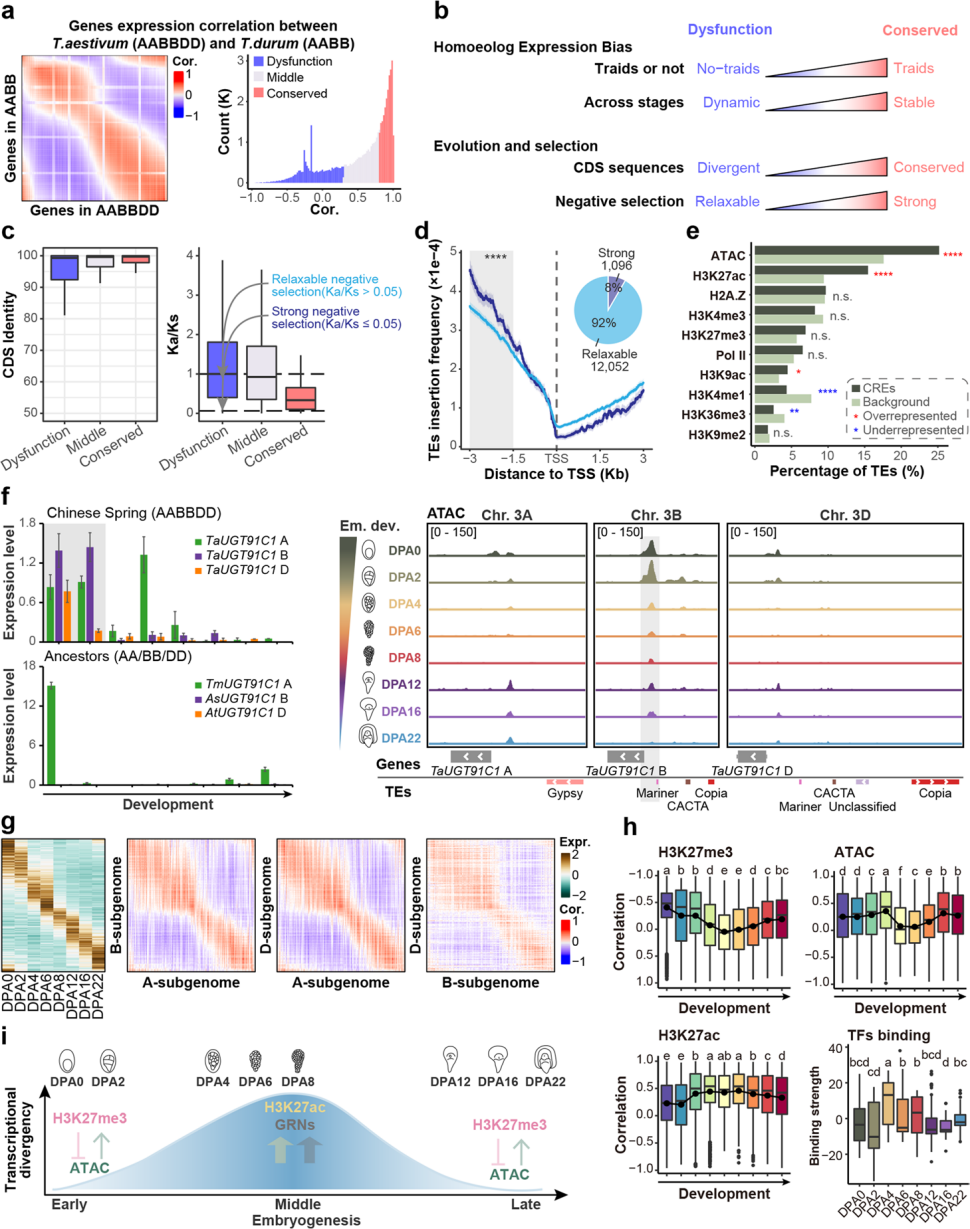
Transcriptional divergence between different ploidy wheat and epigenetic governed stage-specific regulation. **a** Comparisons of gene expressions between hexaploid wheat (AABBDD) and ancestor (AABB). Pearson correlation index was calculated between gene expression in hexaploid wheat and counterpart in ancestor (left). Genes were clustered into three categories based on Pearson index (right). Transcription data was generated from public data [20]. **b** Schematic representation of genomic features differences between dysfunction and conserved cluster genes generated in **a**. Statistics were generated from Additional file 1: Fig. S10b-f. **c**, Coding sequence similarity and Ka/Ks comparisons among different gene sets in **a**. Dysfunction genes could be clustered into genes with relaxing and strong negative selection based on the Ka/Ks value. d The proportion of relaxing and strong negative selection genes in dysfunction gene set and TEs insertion frequency difference in the promoters. **e** Epigenetic modification enrichment at TEs insertion loci. The background generation method is the same as Fig. 2.e. **f** The expression pattern of representative gene TaUGT91C1 in our data and public data (left) and epigenetic regulations (right). **g** Comparisons of homoeologs expressions among subgenomes of hexaploid wheat. Each row represents an individual gene A-subgenome of hexaploid wheat, and the genes were ranked by activated order (left). Pearson correlation index was calculated between homoeologs expression in different subgenomes of hexaploid wheat as those in a. h H3K27me3, chromatin accessibility and H3K27ac contribution to gene expression across wheat embryogenesis and key TFs binding strength at different developmental stages. The top 100 variable motifs calculated by chrVAR were used as key TFs. **i** Stage-specific transcriptional divergence regulation model in wheat embryogenesis. Histone modification H3K27me3 and chromatin accessibility contributed to the early- and late-stage, while H3K27ac and TFs-binding contributed to the mid-stage. Fisher exact test was used for significance calling in **d** and **e** (*p <  = 0.05; **p <  = 0.01; ***p <  = 0.001). Fisher’s least significant difference (LSD) was used in h for significance calling

Polyploidization may drive neo- or sub-functionalization of genes to confer phenotypic plasticity, which can be represented by the varied expression pattern. Instead of identifying genes of differential expression at a specific embryonic stage by comparing hexaploid wheat with tetraploid and diploid [20], we searched for genes exhibiting varied expression profiles during embryogenesis. Indexed by Pearson correlation degree between allohexaploid (AABBDD) and ancestors (AA/BB/DD/AABB), genes were categorized into dysfunction, middle, and conserved clusters (Fig. 7a, Additional file 1: Fig. S10a and Additional file 12: Dataset S11). As expected, conserved genes were mostly homoeolog triads, with sub-genome balanced expression, and showed higher sequence conservation and stronger negative selection pressure (Fig. 7b, c and Additional file 1: Fig. S10b-f). However, a considerable proportion (8%, 1096 genes) of dysfunctional genes changed expression in hexaploid wheat as compared to ancestors, but with slight sequence variation and subject to strong negative selection (Fig. 7c). Promoters of these genes, especially around 1.5 ~ 3 Kb upstream of the TSS, were preferably inserted by TEs (Fig. 7d). Intriguingly, TE insertion regions tended to be accessible chromatin and enriched for H3K27ac (Fig. 7e). For example, *TaUGT91C1* showed a B-subgenome high expression in hexaploid wheat, which is different from its ancestors (Fig. 7f). Accordingly, the promoter harbored TEs with higher chromatin accessibility are found in the B sub-genome compared to their A or D counterparts.

A previous study on embryogenesis revealed a conserved hourglass model in hexaploid wheat and the tetraploid and diploid ancestry, but slightly different for the phylotypic stage [20]. Here, we find a developmental stage-specific pattern for transcriptional divergence. A sharp mid-developmental transition was presented, separating two conserved early and late developmental stages in the comparison between homoeologs in hexaploid per se and between counterparts of hexaploid and its ancestors (Fig. 7a, g, Additional file 1: Fig. S10a, Additional file 12: Dataset S11 and Additional file 13: Dataset S12). We thus hypothesized that different regulatory factors might drive such divergence. Indeed, H3K27ac mainly contributed to the middle stage rather than the early- and late-stage, whereas H3K27me3 and chromatin accessibility behaved oppositely (Fig. 7h). In addition, chromVAR analysis indicated that key TFs function mainly in the middle embryogenesis (Fig. 7h and Additional file 14: Dataset S13). Thus, the stage-specific pattern of transcriptional divergence may be governed by chromatin layer regulation at the early and late stages, while *cis*-motifs and TFs function at the middle stage (Fig. 7i).

## Discussion

### A reference developmental epigenome in wheat for multiple analysis

The scarcity of comprehensive epigenome profiles accompanying plant development has significantly hampered a systematic understanding of the epigenetic regulation of plant developmental processes and prevented a comparative analysis of plant-animal differences. In the present study, we have generated comprehensive epigenomic landscapes consisting of seven types of histone modifications, accessible chromatin, H2A.Z and RNA Pol II occupancy, and resulting transcriptomes across multiple embryonic stages of wheat (Fig. 1a). It is a challenge to dissect and collect enough pure embryo cells for epigenome profiling during early embryogenesis in wheat. We sampled embryo sacs during early embryogenesis from DPA0 to DPA4, whereas isolated embryos for DPA6 and later stages. Based on the PCA and clustering analysis (Fig. 1b, 1e), the chromatin accessibility and transcriptome pattern was not considerably influenced by sampling methods. As well, transitions in both global chromatin states and individual histone modifications were not detected when the sampling method was changed (Fig. 3d, f, h, Additional file 1: Fig. S4b). Thus, though we cannot entirely exclude the potential influence of surrounding tissues on the conclusions generated from early embryogenesis, the above observation indicates that this caveat only minimally affected the depiction of the biological process. This resource presents a comprehensive reference developmental epigenomic landscape in crops and plants. We envisage that this resource would enable the identification of genetic and epigenetic loci critical for embryonic regulatory events in wheat, elucidation of general principle, and regulatory logic of epigenomic dynamics in plant embryogenesis.

Different from animals, plant embryogenesis ends up with a dormancy process without complete organogenesis. Our data suggest that this is associated with chromatin compaction mediated prevention of organ identity gene activation. Meanwhile, the immature embryo is widely used as explant for regeneration and genetic transformation in wheat. However, such capability is dramatically reduced along with the maturation of the embryo. Our epigenome data indicates that key factors, which need to be activated during regeneration, are epigenetically silenced in the mature embryo (Fig. 6d–f). Thus, to a broad extent, the dataset generated in this study could facilitate the understanding of genetic and epigenetic regulation underlying other cellular events beyond embryogenesis, such as organogenesis and plant regeneration (Fig. 6). In addition, such a dataset also allows the comparison of specific epigenomic signatures between wheat and other plant species, as well as plants and animals.

### Conservative and species-specific epigenomic remodeling kinetics during early embryogenesis

Termination of the maternal program and activation of the zygotic genome is a critical process for embryogenesis in plants and animals, along with genome-wide transcriptome alteration [3, 4, 9, 22, 44, 45]. Similar to animals [4, 9], active and repressive histone marks are reprogrammed during MZT process to facilitate the transition between fused gametes to zygotes in wheat (Fig. 3h, Fig. 4b–d, 4h). As well, the chromatin gradually gained accessibility during zygotic activation process in both human [15] and wheat (Fig. 1f, Fig. 2c, Additional file 1: Fig. S3a). However, the remodeling kinetics of individual histone mark is distinct. For instance, H3K4me3 is rapidly removed at promoter after fertilization but re-established during major ZGA in mouse embryos [11, 12]. However, such a pattern was not observed during ZGA in wheat (Fig. 4c), and H3K4me3 showed a mild change during whole embryogenesis (Fig. 3f, 3h). Instead, a quick decline of H3K27ac was observed upon fertilization and then re-established after ZGA (Fig. 3f, h, Fig. 4b, c, i, l and Additional file 1: Fig. S4b), which was not clearly characterized during early mouse or human embryogenesis [4, 8]. It would be interesting to test whether H3K27ac in plants functions as H3K4me3 in mammals during early epigenomic reprogramming. Removing H3K27me3 was detected at the pro-embryo stage at DPA4, while a considerable portion of H3K27me3 was re-established in DPA8 (Fig. 3h and Fig. 4h, j). Such a “rebooting” model is again conserved between animals and plants, while the timing is slightly different [8]. This might be related to the different rhythms of ZGA process in wheat compared to mammals. Importantly, H3K27me3 dynamics mark many essential genes (Fig. 4i, m and Additional file 1: Fig. S5d), indicating its pivotal role in embryogenesis. Indeed, turn-down the function of embryo expressed PRC2 component *FIE* induced a delay in embryonic differentiation, and even aborted embryo development in severe mutant (Additional file 1: Fig. S6). Consistently, recent studies in *Arabidopsis* have reported that a PRC2 component MEDEA, the writer of H3K27me3, is required for embryogenesis [46].

Besides histone modification, variants of Histone 3 (H3.1, H3.3) have been reported to reset during embryonic development in *Arabidopsis* [47]. Here, we did not observe a genome-wide change of histone variant H2A.Z (Additional file 1: Fig. S4c), indicating its less importance. Chromatin accessibility of wheat embryos was gradually built until the transition stage (DPA8) but reduced at the late stage, which is consistent with the chromatin condensation pattern observed in *Arabidopsis* [48]. While it is not entirely surprising that the epigenomic reprogramming during early embryogenesis varied among different species of plants and animals, our findings pinpoint the specific histone modification with a distinct kinetic dynamic that might be involved.

### Three-phase regulatory model for wheat embryogenesis

Embryogenesis is governed by a series of precisely temporally-ordered transcriptional regulation programs [1–3]. Our analyses support a “three-phase regulation model” during embryogenesis (Fig. 7i). Removing local repressive histone modifications is a prerequisite for establishing a permissive chromatin environment, which is the cornerstone of transcription factor-mediated gene regulation. Accordingly, a panorama of genomic and epigenomic dynamics reveals that early embryos are characterized by extensive reprogramming of global histone modifications to establish permissive chromatin (Fig. 1f, Fig. 3h, Fig. 4l, m). Then, extensive bursts of chromatin accessibility and TF-*cis*-elements binding occur at mid-stages (Fig. 5). Finally, repressive histone modifications and compacted chromatin are established at the late stage (Fig. 6 and Fig. 7h, i). Thus, the beginning and end of embryogenesis are dominated by chromatin modification-based regulation, whereas mid-embryogenesis is mainly governed by TF-*cis*-elements regulatory circuits under general accessible chromatin. This regulatory organization is consistent with the stage-specific pattern of transcriptional divergence between allohexaploid and diploid ancestor wheat.

### Insights into the organization and implication of chromatin in a large and polyploid genome

Genome size and ploidy levels affect gene expression and chromatin regulation [49]. Wheat is an allohexaploid species containing three sub-genomes, with a large genome size of about 16.5 Gb, representing an excellent model for studying the epigenetic basis of genome organization and polyploidy. We found that wheat is characterized by a significantly large portion of ACRs and associated histone modifications located in regions distal from gene promoters than other plants with a relatively smaller genome (Fig. 2 and Additional file 1: Fig. S2d). Interestingly, a transient genome-wide open chromatin is observed at the mid-embryonic stage (DPA8), mainly in the distal regions. Such burst of distal accessible regions harbors relatively higher repressive histone modification H3K9me2 and TEs (Fig. 2e, and Additional file 1: Fig. S3d). Moreover, non-coding transcripts are detected at the covered TEs specifically at DPA8 (Fig. 2g). It suggests that the distal chromatin accessibility increase at DPA8 may regulate transient transcription of TEs. Similarly, activation of TEs such as LINE is detected at the 2-cell stage of early embryos in mice, which was highly marked by open chromatin and is essential for ZGA [50, 51]. In *Arabidopsis*, the expression of 24-nt sRNAs reached a peak at the mid-embryonic stage, which further promoted cell-autonomous TE silencing [52, 53]. Further analysis of DNA methylome and small RNA profiling around the transition stage would give a better conclusion of the functional consequence of such dACR eruptions in wheat. Nevertheless, our data uncover a large proportion of distal regulatory elements and highlight their importance in gene and non-coding transcripts regulation in wheat with a large genome.

Another intriguing question is to what extent epigenomic differences could explain the divergence among different sub-genomes. Indeed, we observed that the biased expression of homeolog triads during embryogenesis is correlated with differential histone modifications and chromatin accessibility among A, B, and D sub-genome (Fig. 2h, Fig. 3g, Additional file 1: Fig. S3g, and Additional file 1: Fig. S4d, S4e). A considerable proportion of genes also exhibit varied expression in allohexaploid bread wheat compared with corresponding diploid ancestors [20] (Fig. 7a and Additional file 1: Fig. S10a). Many genes with slight or no sequence variation at the genic region still exhibit divergent expression, indicating involvement of epigenetic regulation. We found that promoters and distal regions of those loci contain distinct TE contents, characterized by accessible chromatin and H3K27ac modification (Fig. 7d, e). Thus, distinct epigenomic landscapes among subgenomes, especially TE inserted active chromatin regions, might contribute to evolutionary transcriptional divergence during wheat embryogenesis.

## Conclusions

Embryo development is one of the most fundamental and remarkable processes in biology. The genetic and epigenetic control of embryogenesis has long been elusive in plant. Here, we generated a reference epigenome for embryo development in wheat, including chromatin state, accessible chromatin regions, and temporal transcription factors-genes regulatory networks. Our data highlighted conservative and species-distinct epigenomic remodeling kinetics during early embryogenesis in wheat, especially for reprogramming of H3K27ac and H3K27me3. By integration of transcriptome and epigenomic data, we uncovered an orchestrated transcriptional regulation by three-phase regulatory models: chromatin accessibility, histone modification, and *cis*–trans regulome. H3K27me3 and chromatin accessibility regulation dominated during the early- and late-embryogenesis, while H3K27ac and gene regulatory networks (GRNs) mainly functioned during mid-embryogenesis, which matched the “stage-specific transcriptional divergence” model.

The common wheat is an allohexaploid plant with three sub-genomes and high proportion TEs occupancy. The epigenomic modification and gene expression were extensively divergent among subgenomes. In addition, the distinct chromatin environment was associated with the biased transcription of homeolog triads. The different TEs occupancy at promoter regions has also potentially influenced the genes’ fate following polyploidization. These insights extend our understanding of the genetics and epigenetic regulation of embryonic development of wheat and provided a wealthy data resource and candidate regulators for functional genomic study.

## Methods

### Plant materials and growth conditions

A spring wheat (*Triticum aestivum*) Chinese Spring was used in this study. All plant was grown in growth room under a 16-h light/8 h dark cycle with light intensity 1000 μmol m^−2^ s^−1^ and temperature 15–26 °C depending on growth stage. The stamens are removed before the pollen maturation. Then, we conduct artificial pollination and record the number of days to ensure the accurate time of seed development. Embryo and embryo sac at the specific developmental stage were sampled for later use of total RNA extraction and nuclei isolation. The dissection method of embryo and embryo sac was largely based on the previous description with some modifications [20, 54]. Embryo sacs containing egg/embryo and surrounding tissue in early stages were dissected in a 5% Sucrose solution which contained 0.1% RNase inhibitor. First, a longitudinal incision was slightly made at the raised surface of the young seed and the transparent embryo was exposed at the tip part of the young seed; the embryo is then released intact by gentle manipulation with fine forceps using the dissecting microscope. For DPA6 and later stages, the embryo was relatively independent and easy to detach from the embryo sac using the same method. Embryos and embryo sacs sampled from eight to ten spikes were pooled for one biological replicate in early stages and three to five were pooled for one biological replicate in late stages. Three biological replicates were generated for RNA-seq samples and two biological replicates were generated for CUT & Tag and ATAC-seq samples.

### RNA extraction, mRNA and non-coding RNA sequencing

Total RNA was extracted using HiPure Plant RNA Mini Kit according to the manufacturer’s instructions (Magen, R4111-02). RNA-seq libraries construction is specified according to different aims. For mRNA sequencing, oligo (dT) was used for enriching the mRNA from total RNA. For non-coding RNA sequencing, rRNA is subtracted by using Ribo-Zero Plus rRNA depletion kit (Illumina), and then fragmentation and random primer was used for reverse transcript process. Sequencing is performed via the Illumina NovaSeq platform by Annoroad Gene Technology [55].

### CUT&Tag experiment

The CUT&Tag experiment was performed as reported with minor modifications [27, 55]. In brief, the fresh samples were soaked in the HBM buffer, chopped with a razor blade, and filtered through a 40-μm cell strainer. The crude nuclei were washed twice and counted by hemocytometer. Extracted nuclei (~ 1000) were resuspended softly in 50 μL antibody buffer containing the corresponding antibody. After overnight incubation at 4 °C, the primary antibody was removed by centrifugation, and the nuclei were incubated in 50 μL wash buffer with secondary antibody at 4 °C for around 1–2 h and then washed twice with wash buffer. A 1:100 dilution of pA-Tn5 complex was prepared in CT-300 buffer, after nuclei were centrifuged, add 100 μL mix and incubate 2–3 h in 4 °C. After incubation with pA-Tn5, wash twice with CT-300 buffer. Then, incubate nuclei in 300 μL Tagmentation buffer at 37 °C for 1 h. To stop tagmentation reaction, add 10 μL 0.5 M EDTA, 3 μL 10% SDS, and 2.5 μL 20 mg/mL Protease K, incubate 1 h in 50 °C. The DNA was extracted with phenol to chloroform to isoamyl alcohol, precipitated with ethanol and resuspended in ddH_2_O. The library was amplified 17 cycles by Q5 high fidelity polymerase (NEB, M0491L) and purified by AMPure XP beads (Beckman, A63881). Finally, the library was sequenced using an Illumina Novaseq platform by Annoroad Gene Technology. All antibody information was list in Additional file 15: Table S1.

### ATAC-seq experiment

The method of nuclei extraction was performed as before [26, 55]. After checking the nuclear integrity, the nuclei extracted (~ 5,000 per reaction) were incubated with the Tn5 transposase and tagmentation buffer at 37 °C for 30 min (Vazyme Biotech, TD501-01) After tagmentation, the DNA is purified by PCR purification kit (QIAGEN, 28,106). PCR was performed to amplify the library for 9–12 cycles using the following PCR conditions: 72 °C for 5 min; 98 °C for 30 s; and thermocycling at 98 °C for 15 s, 63 °C for 30 s and 72 °C for 40 s; following by 72 °C 5 s. After the PCR reaction, libraries were purified with AMPure beads (Beckman, A63881). The library was sequenced using an Illumina Novaseq platform by Annoroad Gene Technology.

### Bioinformatics data preprocessing and alignment

All fastq data, including DNA and RNA sequencing, were generated based on Illumina Hiseq-PE150. Raw data were filtered by fastp (v0.20.0) with “–detect_adapter_for_pe” parameter for reads filter, low-quality bases trimming, and adapters removing [56]. Furthermore, the clean data was evaluated by fastqc software (v0.11.8) (https://github.com/s-andrews/FastQC) to ensure the high quality of reads.

Both DNA sequencing including CUT&Tag, and ATAC-seq and RNA sequencing data were aligned based on *Triticum aestivum* (Chinese Spring) genome assembly (IWGSC RefSeq v1.0) [25], which was downloaded from https://urgi.versailles.inra.fr/download/iwgsc/IWGSC_RefSeq_Assemblies/v1.0/. The IWGSC Annotation v1.1 was used as the gene annotation reference. For DNA sequencing data, BWA-MEM (v0.7.17) algorithm was used for alignment with “-M” parameter to avoid shorter split hits [57]. For RNA sequencing data, hisat2 (2.1.0) was applied for reads mapping with default parameters [58].

### RNA-seq data processing and expression clustering

Sam files generated from hisat2 were converted to bam files without deduplication. FeatureCount v1.6.4 was used for reads quantity per gene [59]. An R package edgeR was used for DEGs (differentially expressed genes) examination, with a threshold absolute value of Log_2_ Fold Change ≥ 1 and FDR ≤ 0.05 [60]. The raw matrix was further normalized to TPM (transcripts per kilobase million) for gene expression quantification. Gene expression data from different tissue was downloaded from a previous publication [43]. TPM values of genes were clustered by k-means method in the heatmap. Modules information generated by WGCNA [61] was used for key gene set selection. For functional enrichment, GO annotation files were generated from IWGSC Annotation v1.1, and an R package clusterProfiler was used for enrichment analysis [62]. For non-coding RNA-seq analysis, hisat2 was used for alignment, and Stringtie (v2.1.4) was used for ncRNA transcriptions assembly [63]. GffCompare (v0.12.6) was used to compare de novo transcriptions with coding genes annotation from IWGSC Refseq v1.1 [25]. The de novo transcriptions that did not overlap with coding genes were selected as ncRNA.

### Cut&Tag and ATAC-seq data processing

Cut&Tag data analysis was largely based on the previously provided pipeline [27]. In brief, two replicates sam files were converted to bam files and sorted by samtools, respectively [64]. We further filter the reads mapped with “samtools view -bS -F 1,804 -f 2 -q 30” to filter the low-quality mapped reads. The high-quality mapped reads were reduplicated using Picard-2.20.5–0 (“Picard Toolkit.” 2019). Two replicate bam files were merged by samtools. For IGV browser visualization, merged bam files were converted to RPKM (Reads Per Kilobase per Million mapped reads) normalized bigwig files with 10 bp bin size for browser visualization by bamCoverage provided by deepTools (3.3.0) with parameters “-bs 10 –effectiveGenomeSize 14,600,000,000 –normalizeUsing RPKM –smoothLength 50” [65]. For Cut&Tag data comparison, scale factors were calculated by ChIPseqSpikeInFree (v1.2.4) [66]. For peak calling, both SEACR v1.3 and MACS2 v 2.1.2 were used [67, 68]. We performed SEACR with numeric threshold 0.05 and normalized stringent model. For narrow histone markers (H3K27ac and H3K4me3) and broad histone markers (H3K27me3), parameters “-p 1e-3” and “–broad –broad-cutoff 0.05” provided by MACS2 were used, respectively. Finally, only peaks generated by MACS2 which overlapped with that generated by SEACR were retained for downstream analysis by “intersect -wa” parameters of bedtools v2.27.1 [69].

The bam file process and bigwig conversion steps in ATAC-seq are the same as that in Cut&Tag. For ATAC-seq peak calling, only MACS2 was used with parameters “–cutoff-analysis –nomodel –shift -100 –extsize 200”. ChIPseqSpikeInFree was not applied in ATAC-seq data process. For both Cut&Tag and ATAC-seq peaks, if a peak overlapped with the proximal of a gene, including 3 kb upstream and 2.5 kb downstream, we assigned the peak to the gene. If multiple genes meet the condition, a position priority strategy (promoter > exon > intron > 5′UTR > 3′UTR > downstream) and nearest gene principle was used for target genes assign. An R package ChIPseeker was used for this peaks annotation process [70].

### Differential chromatin modification enriched regions detection

Reads count under special peaks of Cut&Tag and ATAC-seq were calculated by FeatureCount [59]. For Cut&Tag data, scale factors generated by ChIPseqSpikeInFree were used for differential analysis following the suggested usage of DESeq2 method in ChIPseqSpikeInFree manual [66, 71].

### Chromatin state analysis

For chromatin state analysis, chromHMM was used [31, 32]. “BinarizeBam” and “LearnModel” commands were used for chromatin-state annotation. We used 8 CUT&Tag data as input. Five to twenty-five chromatin-states were generated and we selected the 12-chromatin-states as the final chromatin-states (Fig. 3a left panel).

### ATAC-seq footprints identification

HINT (Hmm-based IdeNtification of Transcription factor footprints) was used for ATAC-seq footprints identity [72]. JASPAR Plantae database (https://jaspar.genereg.net/) was used as motifs set [73]. Custome wheat genome were configurated based on the introduction of HINT software using IWGSC refseq v1.1 Chinese Spring genome.

### Psuedotime indexing and gene regulatory network construction in embryo body formation

Only six-module genes generated from WGCNA were used for the analysis in Fig. 5. The psuedotime indexing method is the same as previous studies [19, 21, 74]. Briefly, principal component analysis (PCA) was used for both RNA- and ATAC-seq data for specific genes. The psuedotime was calculated based on the sample distance between neighbor samples and was scaled to a range from 0 to 10. For one gene, the expression model was fit based on expression level and psuedotime using the “loess” function in R, and 500-time points were generated between 0 to 10, as well as the corresponding expression levels based on the fitted curve. For one gene, the expression level was normalized by *Z*-score. We further calculated the PC1 and PC2 for every gene using the expression values of eight samples. Because the standard expression values of all genes can form a circle, the atan2 function in R was used to return the angle in radians for the tangent PC2/PC1, which were further used for gene expression order ranking. As a result, the psuedotime expression of genes was ranked and visualized by complexHeatmap in R [75]. GO and motif (generated from footprint analysis) enrichment was calculated by clusterProfiler [62].

For gene regulatory network building, the TFs for significantly enriched motifs with *p*-value < 0.05 were used. We further calculated the Pearson correlation between TFs and corresponding targets, and only significant pairs (*p*-value < 0.05) were retained. To simplify the GRN, we focused on the TFs-TFs network. As a result, several TFs contained multiple genes, we combine those as TF modules, including TCPs, ARFs, MYBs, and WOXs.

### Luciferase reporter assays

The pACRs genomic sequence of downstream targets (*TaBBM*, *TaZHD5*, *TaLEC2*, *TaMYB118*, and *TaAMT1*;1) promoter was amplified and fused in-frame with the CP461-LUC vector to generate the reporter construct *target-pro: LUC*. The candidate B3 domain TF binding motifs of TaLEC2 were disrupted by site-directed mutagenesis at the promoter of *TaBBM*, and the *TaBBMmut*-pro:LUC reporter construct was obtained to further confirm the regulatory effect of TaLEC2 on *TaBBM* (Additional file 16: Dataset S14). The CDS of upstream TFs (TaLEC1, TaMYB118, TaZHD5, TaLEC2, and TaWRKY75) was cloned into PTF101 vector to generate the effector construct *35Spro: TF*. Primer sequences were listed in Additional file 15: Table S1. Then, *35Spro:TF* and the reporter vector target-pro: LUC were transformed into *Agrobacterium* GV3101. The *35Spro:GFP* and target-pro: LUC were co-transformed as controls. The bacterial solution was injected to the back of the leaves of *Nicotiana benthamiana* (6–8 leaf stage) using a syringe with the needle removed. The *Nicotiana benthamiana* were cultivated for 2–3 days at a temperature of 22 °C and a light cycle of 16 h light/8 h dark. Firefly luciferase (LUC) and Renilla luciferase (REN) activities were measured using a dual luciferase assay reagent (Promega).

### In situ* hybridization assays*

The in situ hybridization was performed as reported with minor modifications [76]. The seeds of wheat were fixed in formalin-acetic acid-alcohol at 4 °C condition overnight. An ethanol series was used for plant material dehydration. The materials was further embedded in paraffin and sectioned at 8 μm thickness using a histology microtome (RM2235, Leica Microsystems). Sense and antisense RNA probes were designed and synthesized using a DIG northern Starter Kit (Roche) based on the sequence of *TraesCS7D02G305100*. Primer sequences were listed in Additional file 15: Table S1.

### Construction and genotyping of TaFIE-CRISPR/Cas9 lines

For wheat genome editing, plasmid constructs pJIT163-Ubi-Cas9 [36] and pU6-gRNA39 [37] were used. Sequences of PCR primers and other oligonucleotides used for construction are listed in Additional file 15: Table S1. To identify mutations in *TaFIE-7A* (TraesCS7A02G308300), *TaFIE-7B* (*TraesCS7B02G377900LC*), and *TaFIE-7D* (*TraesCS7D02G305100*), gene-specific primers were designed around the gRNA target site. Primers F1 and R were used to amplify *TaFIE-7A*, F2 and R were used to amplify *TaFIE-7B*, and F3 and R were used to amplify *TaFIE-7D*. PCR products were genotyped by Sanger sequencing.

### Histological analysis

Seeds of different DPA time were fixed under a vacuum in FAA solution (5% v/v formaldehyde, 5% v/v acetic acid, 63% v/v ethanol). The samples were then dehydrated through a graded ethanol series and embedded in Technovit 7100 resin (Kulzer, https://www.kulzer-technik.de), according to the manufacturer’s instructions. Sections (2 mm thick) were cut using a UC7&2265 microtome (Leica, http://www.leica-micro systems.com) and stained with 0.02% toluidine blue.

### Quantification of germination rate

Seeds were treated with 1% H_2_O_2_ at 4 °C condition overnight. The germination rate was calculated after germination at room temperature for 2 days.

### Statistics and data visualization

If not specified, R (https://cran.r-project.org/;version 4.0.2) was used to compute statistics and generate plots. For two groups’ comparison of data that fit a normal distribution, Student’s *t*-test was used, such as Fig. 5i, 6e and Additional file 1: Fig. S6e and S6f. For two groups’ comparison of data that does not fit a normal distribution, Mann–Whitney *U* test was used, such as Fig. 2a, 4d and l. For three or more independent groups comparison of data, Fisher’s least significant difference (LSD) was used, such as Fig. 3e, 7h, and Additional file 1: Fig. S4e. For enrichment analysis, Fisher’s exact test was used, such as Fig. 2e, 2f, 4c, 4f, 4j, 4l, 7d, 7e, Additional file 1: Fig. S3b, S3d and S3e. For genomic interval overlapping enrichment analysis in Fig. 2e, 7e, Additional file 1: Fig. S3d and S3e, a background interval was generated using the “shuffle” command in bedtools v2.27.1 [69].

## Peer review information

Wenjing She was the primary editor of this article and managed its editorial process and peer review in collaboration with the rest of the editorial team.

## Review history

The review history is available as Additional file 17.

## Supplementary Information

**Additional file 1: Supplementary Figures S1-S10** (with legends). **Figure S1.** Quality control for CUT & Tag and ATAC-seq. **Figure S2.** Features of various modifications. **Figure S3.** Dynamic ACRs during wheat embryogenesis and difference among sub-genomes. **Figure S4.** Histone modifications dynamic and subgenome comparison. **Figure S5.** Histone modifications reprogramming at pro-embryo. **Figure S6.** Potential function of PRC2 in embryogenesis. **Figure S7.** Inhibition of deep organogenesis in late embryo at chromatin level. **Figure S8.** Chromatin landscape affected sub-genome bias expression. **Figure S9.** Epigenetic modification on different types of homoeologs bias expressed genes. **Figure S10.** Comparisons of embryogenesis among different ploidy wheat.

**Additional file 2: Dataset S1.** The stage-specific pACRs.

**Additional file 3: Dataset S2.** DEGs between DPA2 and DPA0.

**Additional file 4: Dataset S3.** H3K27ac and H3K4me3 decreasing at DPA2 compared with DPA0.

**Additional file 5: Dataset S4.** The correlation between up-regulated genes expression and up-regulated pACRs in DPA2 compared with DPA0.

**Additional file 6: Dataset S5.** The resetting and re-building of H3K27me3.

**Additional file 7: Dataset S6.** Overlap between promoter H3K27me3 resetting and H3K27ac/ATAC-seq up-regulation.

**Additional file 8: Dataset S7.** The synchronization of ATAC-seq and RNA-seq dynamic.

**Additional file 9: Dataset S8.** Selected key modules genes.

**Additional file 10: Dataset S9.** The expression of organ identity genes.

**Additional file 11: Dataset S10.** The expression patterns of candidate organ identity TFs.

**Additional file 12: Dataset S11.** Pearson correlation of gene expressions between hexaploid wheat and ancestor.

**Additional file 13: Dataset S12.** The genes expression rank of homoeologs.

**Additional file 14: Dataset S13.** The deviation scores of key motifs during embryonic development.

**Additional file 15: Table S1.** Antibody and Primer information used.

**Additional file 16: Dataset S14.** Mutation of BBM promoter sequence.

**Additional file 17.** Review history.

## Data Availability

The raw sequence data in this study were deposited in the Genome Sequence Archive (https://bigd.big.ac.cn/gsa) under accession number PRJCA008382 [77]. RNA-seq data of embryo in diploid wheat was download from Gene Expression Omnibus (GEO) under accession number GSE129695 [78]. Code used for all processing and analysis is available under BSD 3-Clause License at Github (https://github.com/LongZhao1992/Dynamic-chromatin-regulatory-programs-during-embryogenesis-of-hexaploid-wheat.git) [79] and Zenodo (https://zenodo.org/badge/latestdoi/582184578) [80].
