# Supplementary material for: Dynamic chromatin regulatory programs during embryogenesis of hexaploid wheat
Source: Genome Biol. 2023 Jan 13;24:7. doi: 10.1186/s13059-022-02844-2 (PMC9837924; doi:10.1186/s13059-022-02844-2)
Supplement: Supplementary file 1 — Additional file 1: Supplementary Figures S1-S10 (with legends). Figure S1. Quality control for CUT & Tag and ATAC-seq. Figure S2. Features of various modifications. Figure S3. Dynamic ACRs during wheat embryogenesis and difference among sub-genomes. Figure S4. Histone modifications dynamic and subgenome comparison. Figure S5. Histone modifications reprogramming at pro-embryo. Figure S6. Potential function of PRC2 in embryogenesis. Figure S7. Inhibition of deep organogenesis in late embryo at chromatin level. Figure S8. Chromatin landscape affected sub-genome bias expression. Figure S9. Epigenetic modification on different types of homoeologs bias expressed genes. Figure S10. Comparisons of embryogenesis among different ploidy wheat. [file 13059_2022_2844_MOESM1_ESM.pdf]

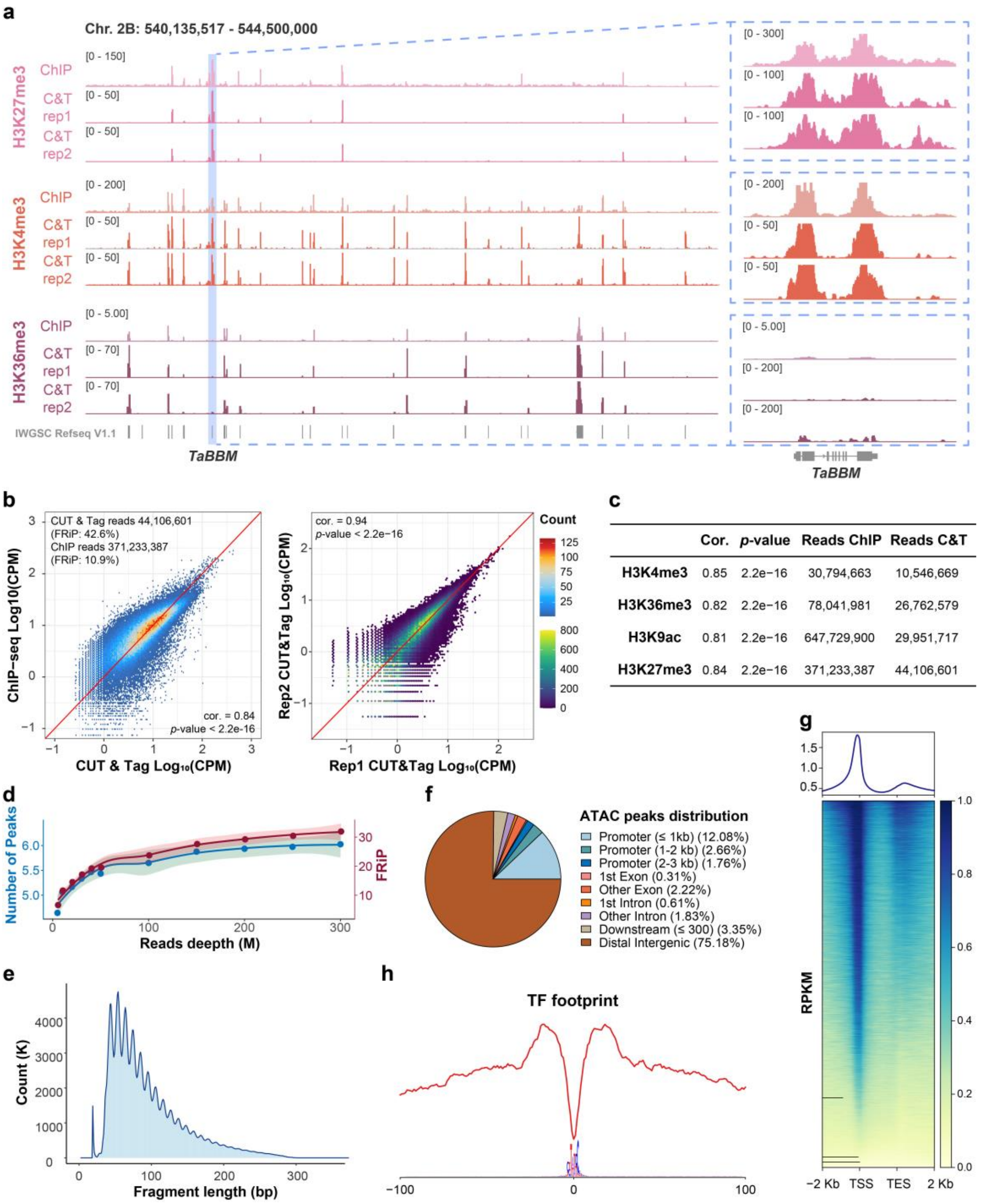

Figure S1. Quality control for CUT & Tag and ATAC-seq. a-c, CUT & Tag recapture ChIPseq signal with high reproducibility and low sequencing depth. IGV (Integrated Genome Browser) showing high reproducibility between ChIP-seq and CUT & Tag in seedlings (a). Correlation between ChIP-seq and CUT & Tag as well as two biological replicates (b). Sequencing depth comparison for various histone modifications between CUT&Tag and ChIPseq (c). d-h, Quality control of ATAC-seq data. Peaks number and FRiP (The fraction of reads in called peak regions) were calculated at different sequence depths (d). Fragment size distribution (e) and peak distribution within the genome (f). ATAC-seq signal distribution along with genes (g). Transcription factor footprint profile of ATAC-seq data (h). "Promoter (<= 1 kb)" represented the peaks with +1~-1 kb to TSS; "Promoter (1-2 kb)" represented the

peaks with -1~-2 kb to TSS; “Promoter (2-3 kb)” represented the peaks with -2~-3 kb to TSS.

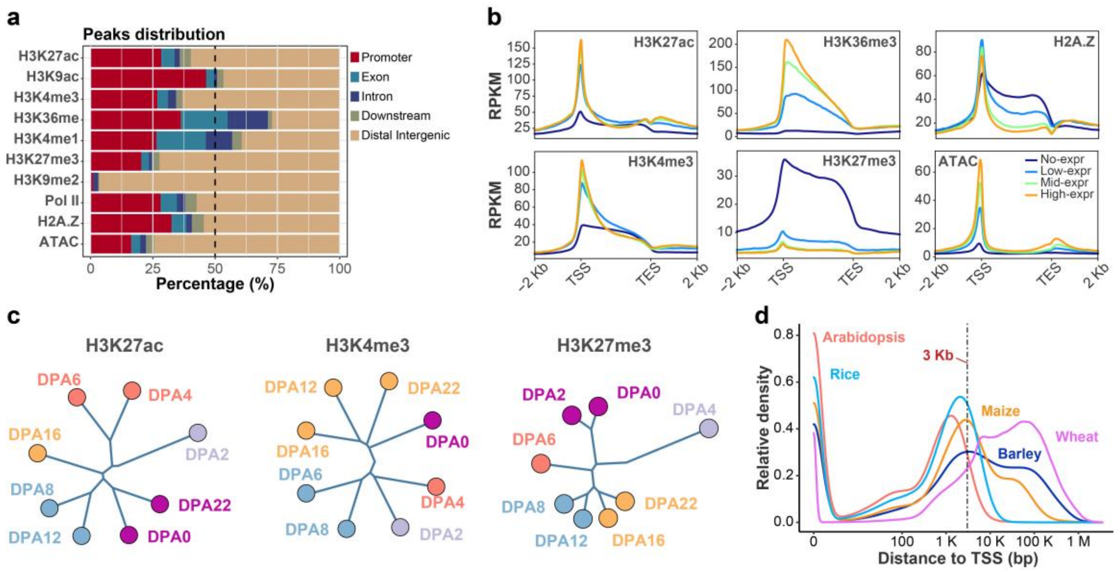

Figure S2. Features of various modifications. a, Peak distribution of different histone modifications relative to genes. b, Correlation between different types of histone modification profiles and gene expression levels. c, Cluster dendrogram of histone modifications. Reads count in peaks were normalized by CPM, and euclidean distances were calculated for tree clustering. Two biological replicates were merged for analysis. d, The ACRs distribution pattern along with the distance to TSS of different species with different genome sizes, including *Arabidopsis*, rice, maize, barley and wheat. Part of raw data from the previous publication [29].

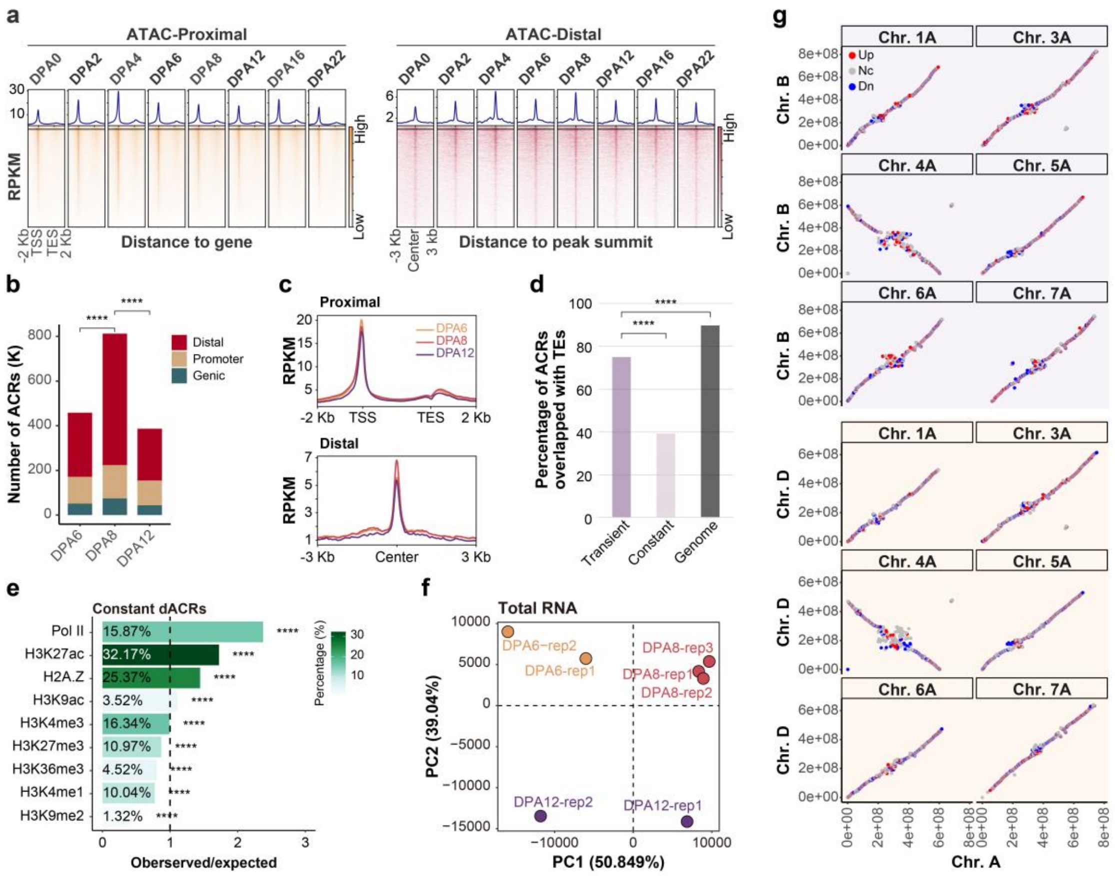

Figure S3. Dynamic ACRs during wheat embryogenesis and difference among sub-genomes. a, ACRs dynamic in proximal (left) and distal regions (right) during wheat embryogenesis. b, The number of different types of ACRs at DPA6, 8, 12. c, ATAC-seq signal in proximal and distal regions at DPA6, 8, 12. d, Percentage of different types ACRs generated from Fig. 2d overlapped with TEs. Transient: DPA8 specific ACRs, Constant: ACRs present in DPA8 and either of DAP6 or DPA12, Genome: randomly select intergenic regions from the genome. e, Histone modifications enrichment at constant dACRs. The analysis method is the same as that in Fig. 2e. f, PCA analysis of total RNAseq data. Two or three biological replicates were used for each development stage. g, Chromatin accessibility (ATAC), histone modifications and various types of TEs distributions change on Chr. 7 of A-, B- and D-subgenome at DPA 6, 8 and 12. Color bars on x and y axis indicate the chromosome segments defined by IWGSC Refseq v1.0. Y axis represented the number of peaks per 10 Mb. Fisher exact test was used for significance calling in b,d and e (* : p <= 0.05; ** : p <= 0.01; ***: p <= 0.001).

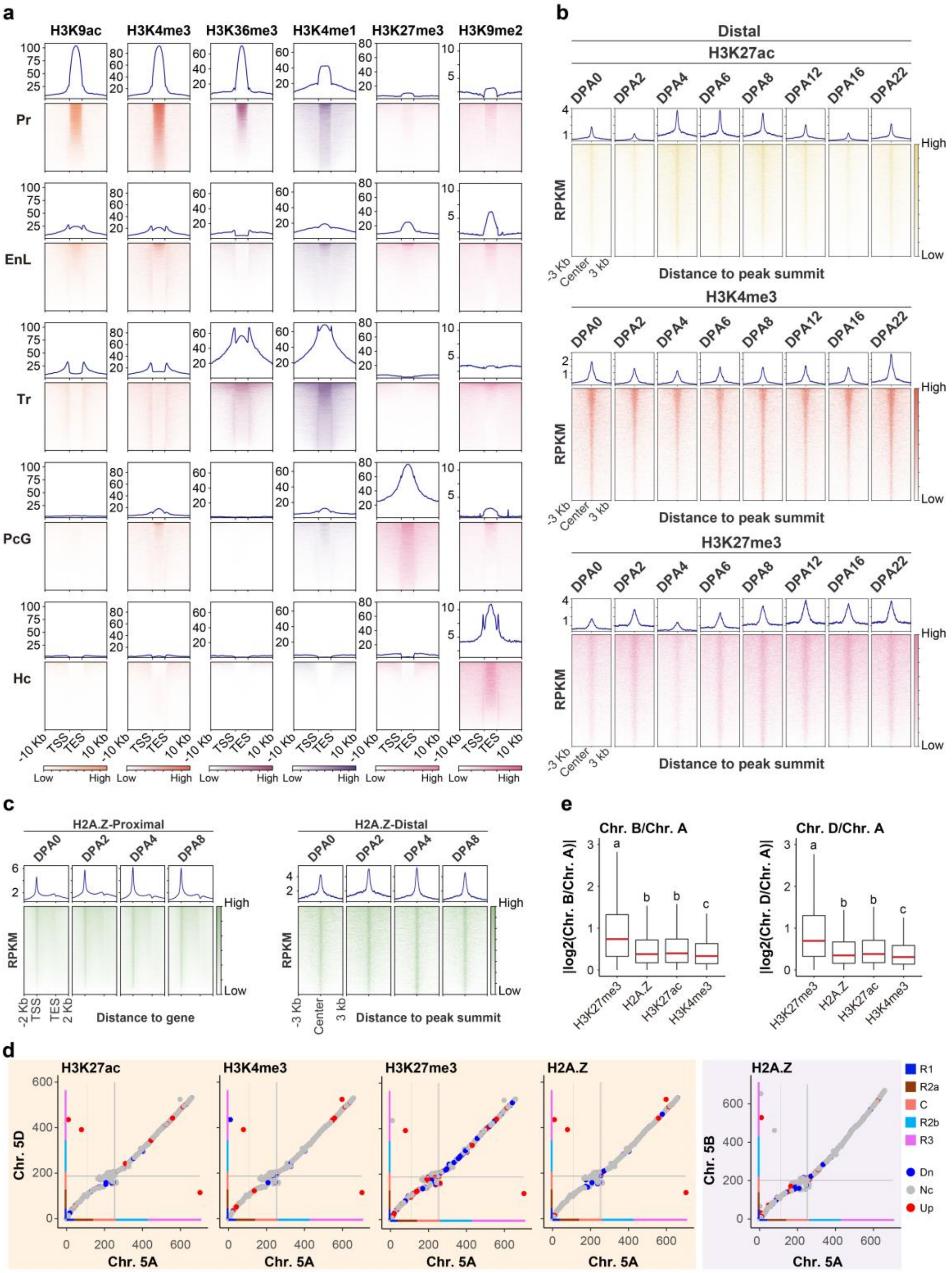

Figure S4. Histone modifications dynamic and subgenome comparison. a, Heatmap showing the histone modification level at different chromatin states. RPKM was used for data normalization. b, Histone modifications dynamic in distal regions during wheat embryogenesis. c, H2A.Z dynamic during wheat embryogenesis. d, Comparisons of histone modifications H3K27ac, H3K4me3, H3K27me3 and H3K9me3 of collinear regions between Chr. 5A and Chr. 5D. Down- (Dn), and Up-regulated (Up) loci were defined by the log_2(fold change Chr. 5D/ Chr. 5A) values bigger and lower than 1 and -1, respectively. Nc indicates no change. Color bars and

grey lines indicate the chromosome segments and centromere regions as those in Fig. 2d. e, Comparison of histone modification signal between subgenomes. Fisher's Least Significant Difference (LSD) was used for significance calling.

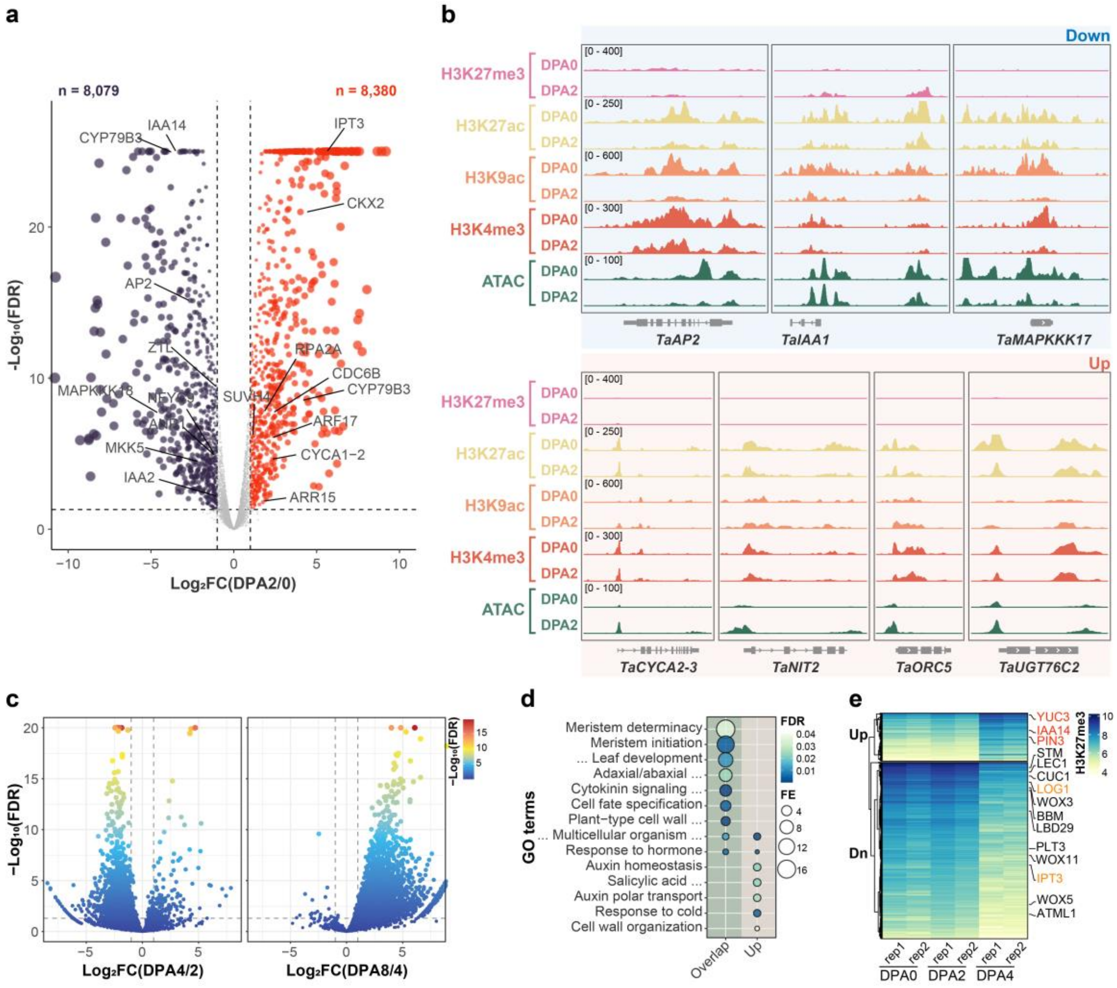

Figure S5. Histone modifications reprogramming at pro-embryo. a, Volcano plot of differentially expressed genes during MZT. DEGs were defined by the threshold value of |log$_2$(Fold Change DPA2/DAP0)| ≥1 and FDR ≤0.05 by edgeR. b, IGV showing histone modifications and chromatin accessibility changes on representative down-regulated genes *AP2*, *MAPKKK17*, as well as *IAA1* (light blue shade), and up-regulated genes *CYCA2-3*, *NIT2*, *ORC5*, as well as *UGT76C2* (light red shade). c, Volcano plot of differential H3K27me3 enriched regions between DPA2 and DPA4 (left), and between DPA4 and DPA8 (right). Differential regions were defined by the threshold absolute value of |log$_2$(Fold Change)| ≥1 and FDR ≤0.05 by DESeq2. d, GO enrichment of gene sets which lost H3K27me3 at DPA4 and re-gain at DPA8, and gene sets which *de novo* gain of H3K27me3 at DPA8. e, Heatmap showing differential H3K27me3 modification genes between DAP4 and DAP2. Embryonic development essential genes were highlighted. Auxin-related genes were labeled in red and Cytokinin-related genes were labeled in orange.

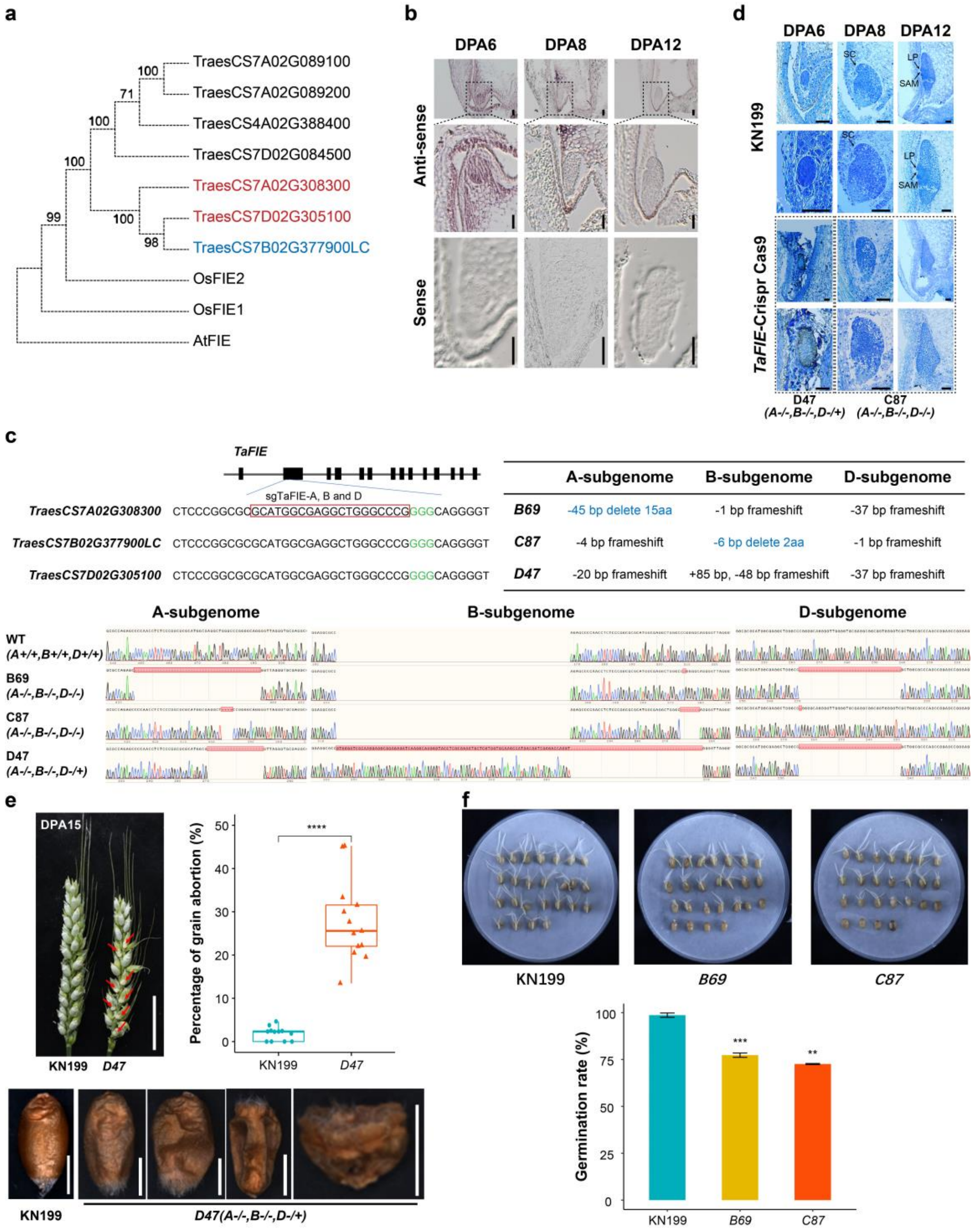

Figure S6. Potential function of PRC2 in embryogenesis. a, Phylogenetic tree showing the identification of *TaFIE*. High-expressed *TaFIEs* were labeled in red color, and the low-confidence gene was labeled in blue color. b, *In situ* assays showing the expression pattern of *TaFIE* (bar = 100 µm). c, Genotyping of different *file* mutate lines. Weak mutat alleles were labeled in blue color. d, Embryonic development of *wt* and *fie* (bar = 100 µm). SC, scutellum; SAM, shoot apical meristem; LP, leaf primordia. e, Grain abortion of *fie*. Grains at DPA15 were shown, and red arrows pointed the arrested developmental seed (up). Grains at DPA35 showed the shrinking phenotype in *fie* mutate lines. More than 15 spikes were counted

for each line. f, The germination rate of *fie* compared with wt. Three replicates were used and 25 grains were used for one replicate. The student's t-test was used for e and f.

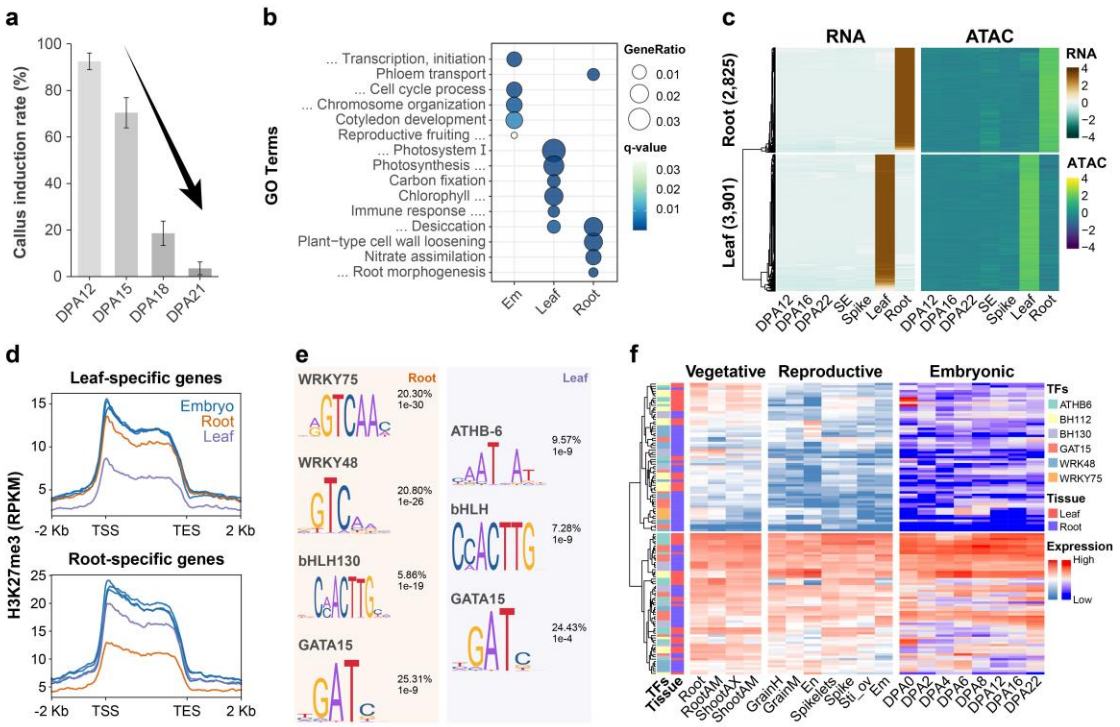

Figure S7. Inhibition of deep organogenesis in late embryo at chromatin level. a, Callus induction rate drops as embryo developing after DPA 12 (three bio-replicates were used, n>50 for each biological test). b, GO enrichment of tissue identity genes found in Fig. 6a. c, Synchronous patterns between root- and leaf-identity genes expression and corresponding pACRs in individual tissue. d, H3K27me3 modification levels on root- and leaf-specific genes in individual tissue. e, Motifs enrichment within regulatory regions of root- (left) and leaf-specific genes (right). Percentage showed the presence frequency, while *P*-value indicates the enrichment. f, Expression patterns of candidate TFs that can bind motifs found in e, cross different tissues and during embryogenesis.

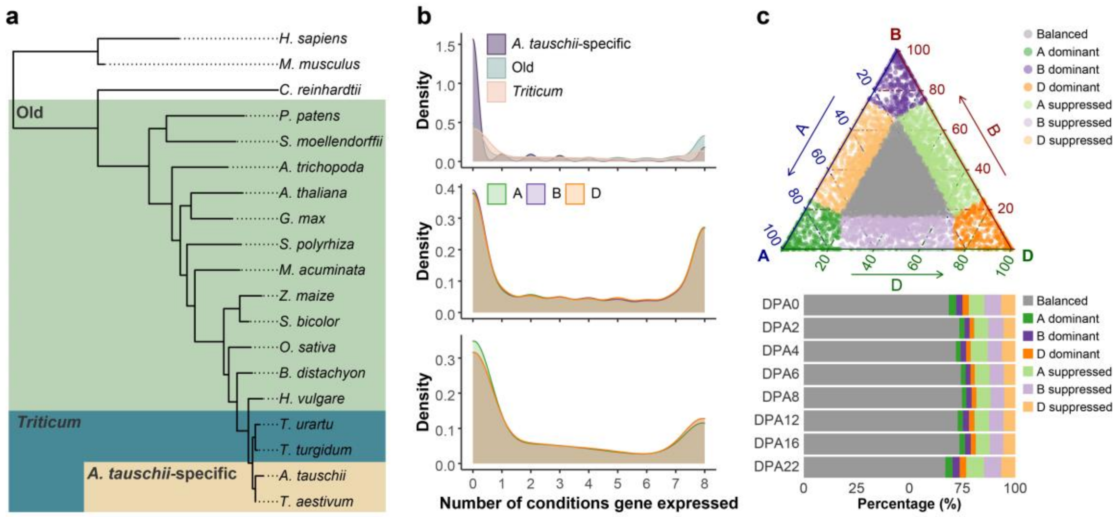

Figure S8. Chromatin landscape affected sub-genome bias expression. a, Phylostratigraphic map of various species. b, Expression spectrum of different evolutionary age genes and sub-genome specific genes in wheat embryo (up- and mid-panel). Expression spectrum of sub-genome specific genes in different environments (bottom-panel). c, Define of homeologs bias expression (left) and percentage of different homeologs bias expression types across eight wheat embryonic developmental stages (right).

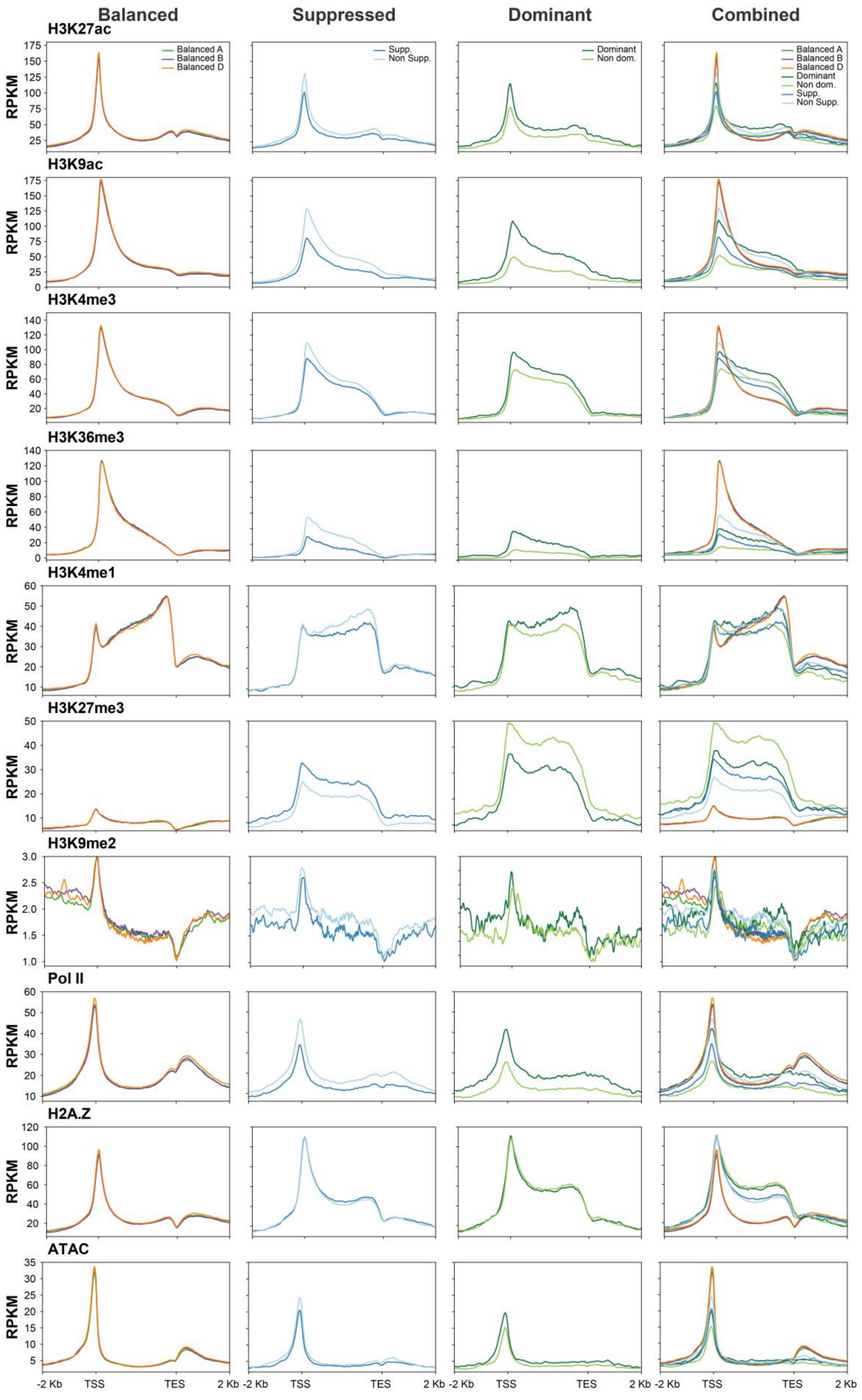
Balanced
Suppressed
Dominant
Combined
H3K27ac
H3K9ac
H3K4me3
H3K36me3
H3K4me1
H3K27me3
H3K9me2
Pol II
H2A.Z
ATAC
RPKM
Balanced A
Balanced B
Balanced D
Supp.
Non Supp.
Dominant
Non dom.
-2 Kb
TSS
TES
2 Kb

Figure S9. Epigenetic modification on different types of homeologs bias expressed genes. Balanced, suppressed, and dominant genes were shown in the first three columns, and a combination of different types of homeologs was shown in the fourth column. Epigenetic modification signals were normalized using RPKM, with 10 bp bin size.

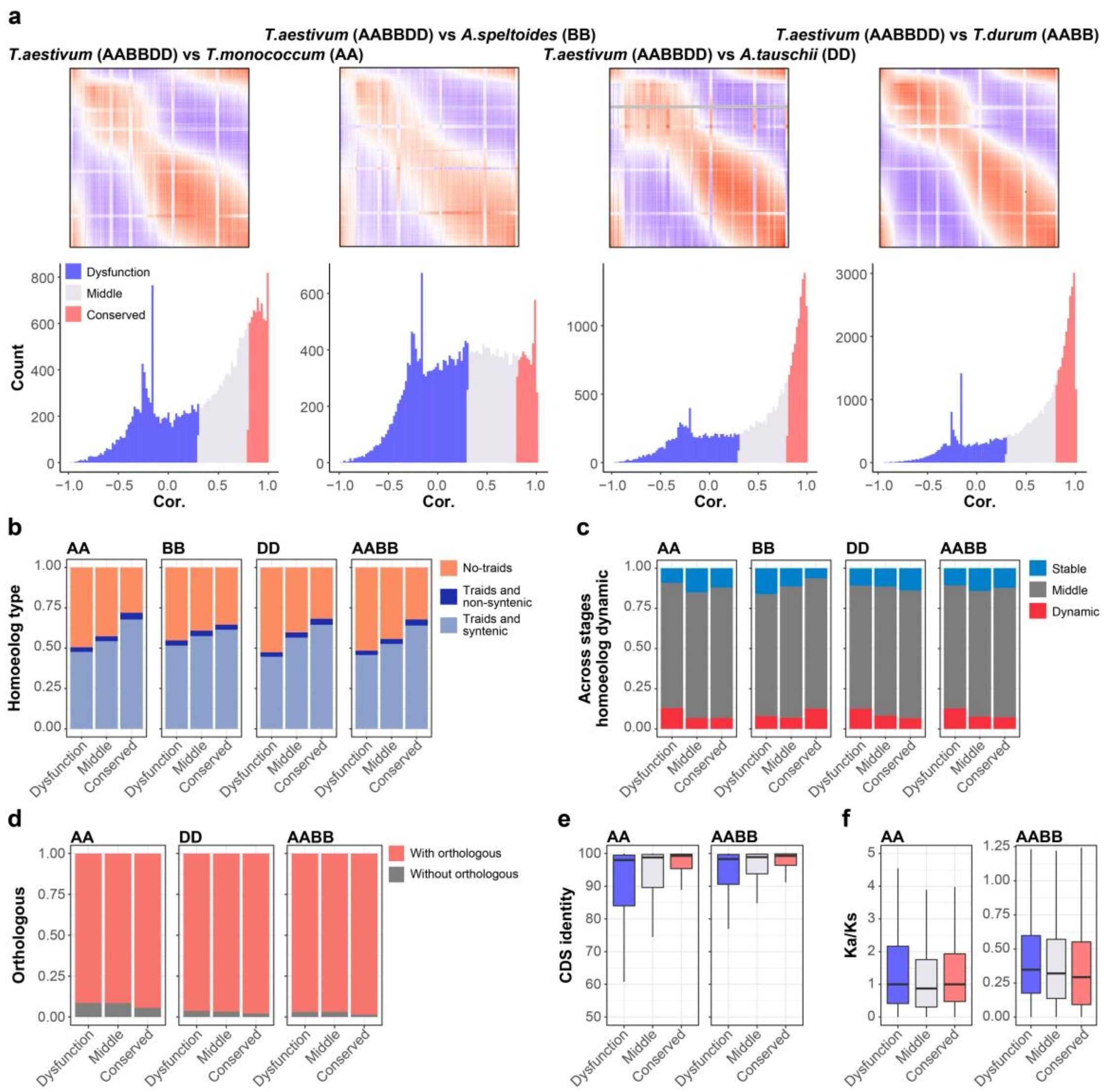

Figure S10. Comparisons of embryogenesis among different ploidy wheat. a, Comparisons of gene expressions between hexaploid wheat (AABBDD) and ancestors (AA, BB, DD and AABBDD) and corresponding Pearson correlation index. Each row represents an individual gene in hexaploid wheat and each line represents an individual gene in the ancestor. Pearson correlation index was from -1 to 1, and indicated by colors from blue to red. Genes were clustered into three categories based on Pearson index from the comparisons between hexaploid wheat and ancestors, which are dysfunction, middle and conserved (bottom). b, c, subgenome synteny (b) and triad dynamic expression across developmental stages (c) for gene set defined in a. d, Percentage of genes has orthologous in corresponding ancestors for gene sets defined in a. e, Sequence similarity between hexaploid wheat and different ancestors for the corresponding gene sets defined in a. f, Ka/Ks comparisons for different gene sets defined in a.
