# Supplementary material for: Dynamic chromatin regulatory programs during embryogenesis of hexaploid wheat
Source: Genome Biol. 2023 Jan 13;24:7. doi: 10.1186/s13059-022-02844-2 (PMC9837924; doi:10.1186/s13059-022-02844-2)
Supplement: Supplementary file 16 — Additional file 16: Dataset S14. Mutation of BBM promoter sequence. [file 13059_2022_2844_MOESM16_ESM.docx]

**Dataset S14. Mutation of BBM promoter sequence**

**>BBM-WT**

GTGTTGGCTCTCACGGTCACAGCAGCAGGGACGCTGCTTTGGACTAACACTCACCGACCAAGTGCTGGATCGGCGCTGATGCATGCATGCATGGATGCATGAAGTAAGATCTTCCGGATCATCATCTTCTTCTTCTTCTTCTTACAGATACATCTCCCTGTAATTACCAAAACATGTTTGATAAAACAACTAGCCGTAAGTCTATGTGTACAATTGCAGCAAATATATACTCCCTCCGTTTCAAAATAGATGATCCAATGGATCATCTAATTTGAAACGGAGGGAGTACTAGCAGCAACGATACTAGCTAGCCCAGCTGTGTGTAGCTGTTTCATTATATCCAGATAATGGCCAACACATCGCTCCTCCTAGCAGCTACTAGTAGCTAGTTGCAGTAGGATGATTAACATTCTCCCCGTAATCATGTATGGTCCCTGCATCTAGCGCTGCACATGACCGGCCGCGAGAGATCGAGGTCGACGGGCGGATCAGGCAGGCATGCATGTTCGCAGAGACGTTTAGGGCACTCCTTTAGTGCGTGGTGCCACTGTTCATCTCCTCTGGTGTGCCCGGAAGACAAGGGTGTGTGTCCTCGTAGAGGGCACAGGTTGCAACCCCAGCTCGCCTACTTTATTCCGAGTCACCGTGAGCTTTCTTTAGGGCTAACCTTTCTCGCTCTTTGGGGCTGGACGGGCGCTTATCGACGATGCCCGCCCGACTGATGCGGGGAAGACAGTGGGTTTGGATCATTTGATTATGCTACCAAGGAATGATCAAGAGATGGTAAATATAATATGCTCGGAGTGAGATCCACACTAGATCGACACTTTCAGTTCTACTCTTGCATTTTCCAGAAGAGATTTGGCAAGTAGCTACATATATTTATGCTTCCATGTACGTACATGAAAAAGAACAGACTCTACAGGCCTGCTAGGTTCCACTTACAACATGAAATCAGATTTTCGACCAGCTCGGCCTGAATTTCTTGCGGTTTGCAGTGAGCCATGTCAGTC

**>BBM-m1m2**

GTGTTGGCTCTCACGGTCACAGCAGCAGGGACGCTGCTTTGGACTAACACTCACCGACCAAGTGCTGGATCGGCGCTGATGCATGCATG----GATGCATGAAGTAAGATCTTCCGGATCATCATCTTCTTCTTCTTCTTCTTACAGATACATCTCCCTGTAATTACCAAAACATGTTTGATAAAACAACTAGCCGTAAGTCTATGTGTACAATTGCAGCAAATATATACTCCCTCCGTTTCAAAATAGATGATCCAATGGATCATCTAATTTGAAACGGAGGGAGTACTAGCAGCAACGATACTAGCTAGCCCAGCTGTGTGTAGCTGTTTCATTATATCCAGATAATGGCCAACACATCGCTCCTCCTAGCAGCTACTAGTAGCTAGTTGCAGTAGGATGATTAACATTCTCCCCGTAATCATGTATGGTCCCTGCATCTAGCGCTGCACATGACCGGCCGCGAGAGATCGAGGTCGACGGGCGGATCAGGCAGGCAT**A**CATGTTCGCAGAGACGTTTAGGGCACTCCTTTAGTGCGTGGTGCCACTGTTCATCTCCTCTGGTGTGCCCGGAAGACAAGGGTGTGTGTCCTCGTAGAGGGCACAGGTTGCAACCCCAGCTCGCCTACTTTATTCCGAGTCACCGTGAGCTTTCTTTAGGGCTAACCTTTCTCGCTCTTTGGGGCTGGACGGGCGCTTATCGACGATGCCCGCCCGACTGATGCGGGGAAGACAGTGGGTTTGGATCATTTGATTATGCTACCAAGGAATGATCAAGAGATGGTAAATATAATATGCTCGGAGTGAGATCCACACTAGATCGACACTTTCAGTTCTACTCTTGCATTTTCCAGAAGAGATTTGGCAAGTAGCTACATATATTTATGCTTCCATGTACGTACATGAAAAAGAACAGACTCTACAGGCCTGCTAGGTTCCACTTACAACATGAAATCAGATTTTCGACCAGCTCGGCCTGAATTTCTTGCGGTTTGCAGTGAGCCATGTCAGTC

Primer sequence：

| BBM-m1-F | TGATACATGCATACATGGAT |
| --- | --- |
| BBM-m1-R | ATCCATGTATGCATGTATCA |
| BBM-m2-F | AGGCAGGCATACATGTTCGCAGA |
| BBM-m2-R | TCTGCGAACATGTATGCCTGCCT |
