# Supplementary material for: Dynamic chromatin regulatory programs during embryogenesis of hexaploid wheat
Source: Genome Biol. 2023 Jan 13;24:7. doi: 10.1186/s13059-022-02844-2 (PMC9837924; doi:10.1186/s13059-022-02844-2)
Supplement: Supplementary file 17 — Additional file 17. Review history. [file 13059_2022_2844_MOESM17_ESM.docx]

Review History

**First round of review**

**Reviewer 1**

**Were you able to assess all statistics in the manuscript, including the appropriateness of statistical tests used?**

No, I do not feel adequately qualified to assess the statistics.

**Comments to author:**

In their manuscript, Zhao and colleagues present epigenomic dynamics during embryogenesis in wheat. This is very impressive work from a technical point-of-view as it is not easy to establish the methods used (e.g. CUT&TAG, ATAC-seq, etc.) from embryos especially at the earliest stages. I agree with the authors that their resource appears to "presents the most comprehensive reference developmental epigenomic landscape in crops". To the best of my knowledge, such comprehensive profiling has not been reported for any plant. Their three-phase model is also interesting and could offer insights into how gene expression programs are established in wheat/monocots. Overall, this manuscript will be of broad interest to those in the fields of epigenetics, development and plant biology.

However, their conclusions are mostly based on correlations from large-scale datasets (without bioreplicates?) from what appears to be different mixtures of seed tissues. In general, it is also unclear how significant many of the associations are both statistically and biologically. More replicates and orthogonal approaches would be needed to support many of the claims made in this manuscript. Alternatively, if bioreplicates showed consistent results then additional experiments may not be needed and the strength of the conclusions could be altered in the text. I have listed these and a few other major/minor comments below to help the authors improve their manuscript.

Major comments

* Were the DPA0, DPA2 and DPA4 samples whole ovules or were the eggs, zygotes and early embryos dissected out before profiling? If eggs/zygotes/embryos were not dissected out, then the reported patterns are confounded by the surrounding tissues. This would be a major caveat to the conclusions drawn from this study and, if true, this limitation should be explicitly stated in the results and methods. For example, on line 225 it is written that "In addition, chromatin states pattern at DPA0 was distinguished from other stages (Fig. 3d), revealing a drastic histone modification reprogramming upon fertilization." But if the chromatin states of whole ovules are being profiled, then this conclusion is not supported by the data presented. Same is true for the data presented in Fig. 4.

* Based on work in Arabidopsis, early embryos are decondensed followed by condensation at the onset of maturation. The authors should cite these previous studies examining chromatin dynamics in Arabidopsis (e.g. Ingouff, 2010, Curr. Biol., van Zanten, 2011, PNAS, Pappareddy, 2020, Genome Biol., etc.) and compare these results with what they found in wheat to highlight similarities and differences that may exist between monocots and dicots.

* How many biological replicates were generated from each stage for ATAC-seq and other methods? It would be informative to see individual bioreps in PCAs (e.g. 1b, 1e) and in Fig. 1f.

* Lines 151-153. Do more ATAC-seq peaks really suggest that there is more open chromatin? It seems that the number of bp that are accessible would be a better indicator of global chromatin dynamics.

Minor comments

* Lines 54-57. Although it is elaborated upon on lines 85-88, the authors should clarify why the comprehensive epigenomes of plant embryogenesis are necessary to reveal differential developmental strategies.

* "Pre-embryo" does not seem like an appropriate term for early embryos.

* Were ATAC-seq peaks detected downstream of TSS or just upstream as indicated in Fig. 1F?

* The lack of correspondence between pACRs and gene expression is unexpected. I would also expect gACRs to be more associated with gene expression since transcription requires chromatin decondensation. Does this mean that cis-regulatory elements are fundamentally different in wheat compared to other plants including rice, Arabidopsis, etc.? Or could there be another (technical) explanation for why dACRs were more associated with gene expression than either pACRs or gACRs?

* Lines 180-181. Please provide a reference that there is "transient expression of TEs during embryogenesis" in plants/wheat. I am not aware of this.

**Reviewer 2**

**Were you able to assess all statistics in the manuscript, including the appropriateness of statistical tests used?**

Yes, and I have assessed the statistics in my report.

**Comments to author:**

In their manuscript Zhao et al., studied the transcriptome trajectory and the chromatin dynamics during embryogenesis of the hexaploid wheat. They performed a time-series analysis which reveals as expected stage-specific and proximal-distal distinct chromatin accessibility and epigenomic dynamics concordant with transcriptome changes. As expected authors also observed that during early embryogenesis, remodeling of H3K27ac, H3K27me3, and chromatin accessibility establish a permissive environment increasing the accessibility of transcription factors to cis-elements.

Overall, the topic is interesting and data quality is very good. However, at the current state, the data presented in this manuscript are only descriptive and the conclusion based correlations. This article may be of interest to the plant community; however, I have concerned that the general principles may not be novel enough for a general audience.

Major comments:

1. Authors wrote "A higher correlation between dACRs, 170 pACRs with gene expression than gACRs was observed (Fig. 2a)." But my question is how they annotated the dACRs and gACRs to the right gene since that several genes could be probably associated to them. How to know when an ACR is not at the proximal part of the promoter of a gene to which gene it should be associated. This question is key and should be answered. This is even more important in the context of development since one regulatory element could be connected to the gene A in one developmental stage and to the gene B in another developmental stage.

2. Authors wrote that "a co-dynamic distribution pattern between ACRs and H3K9me3, but not other histone modifications, was observed at hot spots (Fig. 2c and Fig. S3c)." This result is interesting but the plots fig 2C and S3c are not very clear since we clearly observed that at 8 dpa the ATAC-seq signal is globally higher compare to 6 are 12 and the H3K9me3 signal also. Is it due to a problem of normalization and then an artifact? Or is it true. What is the method used for the normalization and is accurate?

What is the function of H3K9me3? How much H3K9me3 and the ATAC-seq signal correlates? Authors wrote "This may be correlated with the transient expression of TEs during embryogenesis." But where are the data showing a transient expression of TEs during embryogenesis?

3. Authors wrote "reg-ACRs were overlapped with H2A.Z (~15% overlapping), a histone variant with looser DNA wrapping than canonical H2A (Fig. 2i). Reg-dACRs with H2A.Z exhibited higher 'enhancer activity' with more accessibility and higher expression of targeted genes (Fig. 2j, k)." First I do not understand how a region that must be nucleosome free if it is accessible could show a stronger level of H2A.Z. Second, what mean for the authors enhancer activity? An accessible region could enhance or repress transcription. Third, Authors show results with H2A.Z but what about the histone modifications.

4. Authors wrote "Proximal and distal chromatin accessibility undergoes distinct reprogramming during embryogenesis, with different transcriptional regulation readout." I do understand the authors would like to express with this sentence.

5. I have serious doubt regarding the chromatin states performed by the authors. First, how authors did to start with 15 and end with 5 major functional classes. Second, authors wrote "enhancer-like classes were enriched for active histone modifications such as H3K27/9ac, H3K4me1/3". It would be great that authors generate heat maps centered (i) on enhancer-like region, (ii) promoter, (iii) transcriptional unit, (IV) on polycomb and (V) on TE to show how the level of each histone modification looks. This will allow the reader to see if a correlation exists. Of course the different regions should overlap.

6. Authors wrote that "we further examined H3K4me3 and H3K27me3 co-occupied bivalent regions" but where is the experiment that proved that H3K4me3 and H3K27me3 co-occupied bivalent regions and that it is not the signal observed because of the mixture of different cell type where in one cell type the region is only marked by H3k4me3 and in another cell type the same region is marked by H3K27me3?

7. Authors wrote "In both proximal and distal bivalent regions, we observed widespread ATAC-seq, H3K27ac, 247 H3K4me1, H3K9me3, Pol II, and H2A.Z signals, but not transcripts and transcription-associated H3K36me3, suggesting a transcriptionally poised state (Fig. 249 3g and Fig. S5b)." I do not understand what authors mean by "in both proximal and distal bivalent regions". Authors talk about enhancer or ORF? If it is enhancer why looking for transcripts? If it is ORF why talking about "proximal and distal"

8. Authors wrote "H3K27me3 decreased at both up- and down-regulated genes but more obvious for up-regulated genes." How do they explain this since this mark is known to be associated with gene repression?

9. Authors wrote "Collectively, reprogramming of histone modification, particularly H3K27ac and H3K27me3, and establishing chromatin accessibility at early embryo development ensures the proper transition from maternal to zygotic process and initiation of the embryo development." While causation and correlation can exist at the same time, correlation doesn't mean causation. To prove that authors must use genetics.

10. Authors wrote "Interestingly, TaLEC2 could bind the pACR of BBM directly (Fig. 5i)." I do not see any data showing that TaLEC2 could bind the pACR of BBM.

**Authors Response**

**Point-by-point responses to the reviewers’ comments:**

Reviewer #1:

In their manuscript, Zhao and colleagues present epigenomic dynamics during embryogenesis in wheat. This is very impressive work from a technical point-of-view as it is not easy to establish the methods used (e.g. CUT&TAG, ATAC-seq, etc.) from embryos especially at the earliest stages. I agree with the authors that their resource appears to "presents the most comprehensive reference developmental epigenomic landscape in crops". To the best of my knowledge, such comprehensive profiling has not been reported for any plant. Their three-phase model is also interesting and could offer insights into how gene expression programs are established in wheat/monocots. Overall, this manuscript will be of broad interest to those in the fields of epigenetics, development and plant biology.

*Reply: Thanks for the encouraging comments on our work.*

However, their conclusions are mostly based on correlations from large-scale datasets (without bioreplicates?) from what appears to be different mixtures of seed tissues. In general, it is also unclear how significant many of the associations are both statistically and biologically. More replicates and orthogonal approaches would be needed to support many of the claims made in this manuscript. Alternatively, if bioreplicates showed consistent results then additional experiments may not be needed and the strength of the conclusions could be altered in the text. I have listed these and a few other major/minor comments below to help the authors improve their manuscript.

*Reply: Thanks very much for your comments, we didn’t describe in a clear way. For all RNAseq, three bio-reps were sequenced, while for ATACseq and CUT&Tag, two bio-reps were conducted. We have added the PCA for individual bio-replicates in Fig. 1b,e,f, and with proper statistical analysis throughout the manuscript in the revision.*

Major comments

* Were the DPA0, DPA2 and DPA4 samples whole ovules or were the eggs, zygotes and early embryos dissected out before profiling? If eggs/zygotes/embryos were not dissected out, then the reported patterns are confounded by the surrounding tissues. This would be a major caveat to the conclusions drawn from this study and, if true, this limitation should be explicitly stated in the results and methods. For example, on line 225 it is written that "In addition, chromatin states pattern at DPA0 was distinguished from other stages (Fig. 3d), revealing a drastic histone modification reprogramming upon fertilization." But if the chromatin states of whole ovules are being profiled, then this conclusion is not supported by the data presented. Same is true for the data presented in Fig. 4.

*Reply: We thank reviewer for raising this important issue. Two different sampling strategies were used, in which embryo sac without the seed coat were used for profiling at stages from DPA0 to DPA4, and the isolated embryos were used for the rest stages (DPA6-22). Because a minimal number of 1,000 and 5,000 intact nuclei are required for CUT&Tag and ATAC-seq experiments in our experimental system, the technical challenge prevented us from isolating the embryos from surrounding tissues at earlier stages (DPA0-4). We agree with this reviewer that the mix of embryos with surrounding tissues would likely compromise the accuracy of epigenomic profiling at earlier stages; nevertheless, for the following reasons, we think this caveat only minimally affected the major conclusions of this study.*

*First, as demonstrated in PCA and clustering results, the similarity between histone modification patterns is not considerably influenced by sampling methods. Although embryos were isolated at DPA6 but not at DPA2 and 4, the chromatin accessibility and transcriptome at DPA4 is more similar to DPA6 than to DPA2 (Fig. 1b,1e). In addition, temporal expression of several marker genes, such as TaWOX11, TaLEC2, TaARR6, and TaABI5, exhibited similar patterns to that in B. distachyon and A.thaliana (Fig. 1d), in which the embryos were isolated [1,2]. The above results suggest that the embryo rather than the surrounding tissues dominated the results.*

*Second, transition points of both global chromatin states and individual histone modifications were not detected when the sampling method was changed (before or after DPA4). For example, transitions of chromatin states were largely observed between DPA0/DPA2, DPA0/DPA4, but not DPA4/DPA8 (Fig. 3e). Similarly, transitions of histone H3K27ac was mainly observed between DPA2/DPA4 or DPA8/DPA12 (Fig. 3f, Fig. S5a) and H3K27me3 were dramatically decreased between DPA2/DPA4 and greatly increased during DPA6-8-12(Fig. 3f, Fig. S5a). All of the above transitions occurred between stages in which an identical sampling method was applied. By contrast, H3K27ac, H3K4me3 and H3K9me3 don’t change much from DPA4 (embryo sac) to DPA6 (embryo) (Fig. 3f, Fig. S5a).*

*Third, in Figure 4, we mainly analyzed the dynamic of H3K27ac, H3K27me3 and chromatin accessibility on regulation of gene expression from DPA0 to DPA4 (Fig. 4a-g,k,l), of the same sampling strategy. For H3K27me3, we found dramatic reduction from DPA2 to DPA4, both are embryo sac samples (Fig. 4h-j), though we added DPA8 (embryo) for indicating the restoration of H3K27me3. This is also not likely to be artifact, since from DPA6 to DAP8-12 (all are embryo samples), we observed gradually increased H3K27me3 coverage (Fig. S5a).*

*In summary, while we cannot entirely exclude the potential influence of surrounding tissues on the results at DPA0-4, the above observations suggest that they likely minimally affected our conclusions. We have now clarified in the Methods (Line 587-595) that surrounding tissues were included in early samples, and indicated the different sampling for early and other embryogenesis samples in Result (Line 134-135) and added a paragraph describing the above caveat in the Discussion (Line 463-473).*

* Based on work in Arabidopsis, early embryos are decondensed followed by condensation at the onset of maturation. The authors should cite these previous studies examining chromatin dynamics in Arabidopsis (e.g. Ingouff, 2010, Curr. Biol., van Zanten, 2011, PNAS, Pappareddy, 2020, Genome Biol., etc.) and compare these results with what they found in wheat to highlight similarities and differences that may exist between monocots and dicots.

*Reply: We gratefully appreciate your valuable suggestion. We have cited previous papers on Arabidopsis and rice and made several comparisons between monocots and dicots in the Discussion (Line 504-509, and 647-651).*

* How many biological replicates were generated from each stage for ATAC-seq and other methods? It would be informative to see individual bioreps in PCAs (e.g. 1b, 1e) and in Fig. 1f.

*Reply: Thanks for your careful check. We did three bio-reps for RNAseq and two bio-reps for ATACseq and CUT&Tag, and have added the information regarding biological replicates in Method (Line 586-588), Result (Line 136-137), and Fig 1 and Fig S2 legends. PCA of individual bio-reps was added in Fig. 1b,1e, 1f.*

* Lines 151-153. Do more ATAC-seq peaks really suggest that there is more open chromatin? It seems that the number of bp that are accessible would be a better indicator of global chromatin dynamics.

*Reply: Thanks for your suggestion. We have added base-pair coverage of accessible chromatin regions in Fig. 1f in addition to peak numbers from two bio-reps. The conclusion was the same as the peak number.*

Minor comments:

* Lines 54-57. Although it is elaborated upon on lines 85-88, the authors should clarify why the comprehensive epigenomes of plant embryogenesis are necessary to reveal differential developmental strategies.

*Reply: Thanks. We have added the importance of epigenetic research in plant embryogenesis in line 42-46. In brief, time-series epigenome research emphasizes the importance of chromatin level regulation of early embryogenesis in mammalians. While in plant due to technique challenge, such epigenomic study is lagged. Thus, it is necessary for plant epigenome data generation during embryogenesis.*

* "Pre-embryo" does not seem like an appropriate term for early embryos.

*Reply: Thanks. We have changed this term as “pro-embryo” according literatures in flowering plants[3,4].*

* Were ATAC-seq peaks detected downstream of TSS or just upstream as indicated in Fig. 1F?

*Reply: Thanks, we have shown the ATAC-seq peaks distribution at whole genome level and gene coding regions in Fig. S1f, g. Generally, ATAC-seq peaks were detected mainly in the distal intergenic region (>75%), which is defined as more than 3Kb away from TSS (Fig. S1f). At gene coding region, we could observe highly enriched peak covering both up- and down-stream of TSS, as well as a small enriched peak around TES (Fig. S1g). Such pattern is consistent with finding in other plant species [5]. We have added detailed information to the legend of Fig. S1f, S1g.*

* The lack of correspondence between pACRs and gene expression is unexpected. I would also expect gACRs to be more associated with gene expression since transcription requires chromatin decondensation. Does this mean that cis-regulatory elements are fundamentally different in wheat compared to other plants including rice, Arabidopsis, etc.? Or could there be another (technical) explanation for why dACRs were more associated with gene expression than either pACRs or gACRs?

*Reply: Thank you for the comment. We would like to clarify that, as demonstrated in revised Fig. 2a, pACR was indeed associated with gene expression. In each sample, genes with pACRs were expressed significantly higher than those without a pACR (12.2 v.s. 1.94 Mann–Whitney U test, p < 0.00001)(Revised Fig. 2a). Furthermore, 20,871 of pACR was positively correlated (mean of R = 0.81, p < 0.05) with gene expression across stages, while only 3,182 pACR was negatively correlated. Using the same strategy, we compared the contribution of gACRs to gene expression, revealing that although gACRs were also associated with gene expression, they were not as prominent as pACRs (Revised Fig. 2a).*

*We guess this reviewer’s impression of dACRs being more correlated with gene expression than pACRs and gACRs came from Fig. 2a, where the best correlation coefficients between all types of ACR and gene expression were compared. Because of the uncertainty in assigning a dACR to the gene it potentially regulates, all genes within a 0.5-Mb region around a dACR were analyzed, and the one that exhibited the significant correlation (R>0.7) was selected to represent a dACR’s effect. While this strategy is valid for estimating the contribution of dACRs to gene expression, we have now realized that it is unfair to compare the effects of different ACR types, as the effects were assessed for multiple genes for dACRs but not for pACRs and gACRs.*

*In the revised manuscript, we only used the scanning strategy to demonstrate that a considerable portion of dACRs potentially contributes to gene regulation (Fig. 2a, 2g), with the comparison result generated by this strategy removed (Line 177-181). In addition, we added a new result showing that wheat ACRs tended to be far from the transcription start site (TSS) compared to other plants (Revised Fig. S2e) (Line 164-167). This result better motivates the need to analyze the gene regulation effects of dACRs in the wheat genome.*

* Lines 180-181. Please provide a reference that there is "transient expression of TEs during embryogenesis" in plants/wheat. I am not aware of this.

*Reply: Thanks, we agree with the reviewer that so far no such work is reported in plants/wheat. This is speculation based on research in animal field. TEs was activated at the 2-cell stage of early embryos in mouse, which was highly marked by open chromatin [6–9]. We think this is a very interesting topic to follow, so we did non-coding RNAseq for DPA6-8-12 to uncover the non-coding transcripts from those intergenic regions. As shown in revised Fig. 2d, Fig. S3h, we did find burst of non-coding transcript at DPA8 around those opened chromatin regions. See more details in below part for answering reviewer #2’s question 2.*

Reviewer #2:

In their manuscript Zhao et al., studied the transcriptome trajectory and the chromatin dynamics during embryogenesis of the hexaploid wheat. They performed a time-series analysis which reveals as expected stage-specific and proximal-distal distinct chromatin accessibility and epigenomic dynamics concordant with transcriptome changes. As expected authors also observed that during early embryogenesis, remodeling of H3K27ac, H3K27me3, and chromatin accessibility establish a permissive environment increasing the accessibility of transcription factors to cis-elements.

Overall, the topic is interesting and data quality is very good. However, at the current state, the data presented in this manuscript are only descriptive and the conclusion based correlations. This article may be of interest to the plant community; however, I have concerned that the general principles may not be novel enough for a general audience.

*Reply: We thank the reviewer for the positive comments on the topic and the data quality of this study. The major criticism raised by this reviewer concerns the descriptive nature of the results and the limited novelty of the findings to a general audience. Below, we explain why we think this study provides novel insights into the epigenomic regulation of plant development.*

*First, our study presents the most comprehensive epigenomic datasets accompanying plant embryogenesis, including genome-wide patterns of eight types of important histone modifications, H2A.Z histone variant, and chromatin accessibility. This is also validated by reviewer #1 as saying “To the best of my knowledge, such comprehensive profiling has not been reported for any plant.” This novel resource could serve as a reference to elucidate the epigenomic regulation of wheat development and to perform comparative epigenomics between plants and animals.*

*Second, we revealed important features of epigenomic dynamic that distinguish the early embryogenesis of wheat from that of mouse or human. Specifically, H3K4me3, an active histone mark, is rapidly removed at promoter after fertilization but re-established during major ZGA in mouse embryos [10–12]. However, only mild change was observed during wheat early embryos (Fig. 3f), especially not gained for the genes activated during ZGA (Fig. 4b). Moreover, a quick decline in H3K27ac level, an active histone mark, was observed after wheat fertilization then re-established after ZGA (Fig. 3f; Fig. 4b, 4i, 4l; Fig. S5a), which was not clear characterized during early mouse or human embryogenesis [10,13]. Moreover, a global erasure of H3K27me3 at promoter region, a well-known heterochromatin mark associated with gene repression, was observed during early ZGA in mouse and human [11,14]; however, no global drop was detected in wheat embryos until the DPA4 (Fig. 3f; Fig. S5a), pro-embryonic stage. While it is not entirely surprising that the epigenomic reprogramming during early embryogenesis differs between plant and animal species, our findings pinpoint the specific histone modifications with distinct kitnetic dynamic that might be involved. Although the detailed mechanisms underlying the differences remain to be elucidated, we feel the above findings would be thought-provoking and of broad interest to the audience outside the plant community. For example, it will be interesting to test in follow-up studies whether H3K27ac in plants functions as H3K4me3 in mammals during early epigenomic reprogramming.*

*Third, our findings provide insights into the organization and implication of chromatin in a large and polyploid genome. Wheat is an allohexaploid species containing three sub-genomes, with a large genome size of 16.5 Gb, more than five folds the human genome size, representing an excellent model for studying the epigenetic basis of genome organization and polyploidy. We reveal epigenomic characteristics associated with the large genome and the functional divergence between sub-genomes. We found that wheat is characterized by a significantly large portion of accessible chromatin and associated histone modifications located in regions distal from gene promoters than other plants with a relatively compact genome (Fig. 2, Revised Fig. S2e). Notably, distal accessible chromatin exhibited extensive developmental dynamics and was associated with gene expression (Fig. 2 g-k), indicating distal chromatin regulation is likely a common theme for regulating gene expression in large genomes. Moreover, we characterized epigenomic signatures correlated with biased expression among sub-genomes (Fig. 7g; Fig. S9) and divergent expression after polyploidization (Fig. 7a, 7f; Fig. S10a). These findings reveal an epigenomic basis of subgenome diversification in a polyploid genome.*

*Fourth, different from animals, plants embryogenesis is decoupled with deep organogenesis, end up with dormancy, a ‘quiescent status’ that allows plant seeds (mature embryo with endosperm and seed coat) to survive in harsh environmental conditions. Our data suggests that the lack of deep organogenesis is associated with H3K27me3 repression and compaction chromatin environment at the promoters of organ identity genes (Fig. 6b, c), which preventing specific transcription factors binding (Fig. 6e,f). Meanwhile, immature embryo is widely used as explants for regeneration and genetic transformation in wheat [15]. However, such capability for regeneration is reduced along with the maturation of embryo (Fig. S7a). Our epigenome data indicates that key factors, which need to be activated during the process of regeneration, are epigenetically silenced in mature embryo, with reduced chromatin accessibility (Fig. 6d). Thus, this finding could be useful for improving wheat regeneration efficiency, such as increasing the chromatin accessibility while induction of regeneration from mature embryo.*

*We have now significantly revised the data presentations and texts in the Results and Discussion sections to highlight the above novelties of our study better, and we hope these clarifications satisfactorily address this review’s concerns.*

*Finally, we agree with this reviewer that most of the conclusions of this study are descriptive in nature and are based on correlative analyses. However, we hope this reviewer could understand that genetic manipulation in wheat presents a significant challenge due to the polyploid genome as well as the long experimental period [16]. Taking the histone writers as an example, the wheat genome encodes nine genes responsible for adding the H3K27me3 modifications. Such extensive functional redundancy prevented us from performing genetic perturbation experiments to establish and validate causal relationships between chromatin states/histone modifications and developmental regulation.*

Major comments:

1. Authors wrote "A higher correlation between dACRs, pACRs with gene expression than gACRs was observed (Fig. 2a)." But my question is how they annotated the dACRs and gACRs to the right gene since that several genes could be probably associated to them. How to know when an ACR is not at the proximal part of the promoter of a gene to which gene it should be associated. This question is key and should be answered. This is even more important in the context of development since one regulatory element could be connected to the gene A in one developmental stage and to the gene B in another developmental stage.

*Reply: Thank you for this question. ACRs at proximal regions were assigned to the nearest genes. The ACRs located at -3Kb to +1Kb to TSS of genes were defined as pACRs. For ACRs that overlapped with genes’ body, or located within 2.5 Kb from TES of genes wre defined as gACRs. The annotation priority is pACRs, gACRs and dACRs. For dACRs, all genes within a 0.5-Mb region around a dACR were analyzed, and the ones that exhibited a significant correlation were selected as the targets (Fig. S4c). Therefore, one gene could have multiple dACRs and one dACRs could have multiple target genes (Fig. S4i,j,k). The similar correlation-based assignment strategy has been used in published literature [17–19].*

*We agree with the reviewer that the assignment of dACRs to target genes is important for gene regulation during development. Thus, we did in silico validation of the dACR-gene links by public Hi-C data in wheat and dACRs homologous segment in maize and rice which has public Hi-C and ChIA-PET data (Fig. S4 d,e). All the evidence demonstrated that our assignment method is logical. However, we cannot answer the question that “one regulatory element could be connected to gene A in one developmental stage and to gene B in another developmental stage” using this correlation method from our current data. The main reason is that we don’t have enough samples from varied tissues of different developmental stages to do the comparison. This question may be solved by multiple Hi-C/ChIA-PET data from diverse tissues and several ATAC-seq and RNA-seq data.*

*While this strategy is valid for estimating the contribution of dACRs to gene expression, we have now realized that it is unfair to compare the effects of different ACR types. Thus, we removed the comparison in Fig. 2a in the revised manuscript. In addition, we describe the dACR-assignment strategy in more detail in the Result (Line 208-210).*

2. Authors wrote that "a co-dynamic distribution pattern between ACRs and H3K9me3, but not other histone modifications, was observed at hot spots (Fig. 2c and Fig. S3c)." This result is interesting but the plots fig 2C and S3c are not very clear since we clearly observed that at 8 dpa the ATAC-seq signal is globally higher compare to 6 are 12 and the H3K9me3 signal also. Is it due to a problem of normalization and then an artifact? Or is it true. What is the method used for the normalization and is accurate?

*Reply: Thanks for this critical question. We believe that the dynamic patterns of ACRs and H3K9me3 did not result from normalization. Firstly, all the ATAC-seq peaks were called using the same threshold, and both biological replicates showed increased peak number at DPA8 (Fig. 1f). Secondly, the Y-axis represented the number of peaks per 10-Mb bin. This normalization method has been reported in previous studies[20,21].Thirdly, if the increase of ACRs at DPA8 was generated from the normalization artifact, we expect this increase to be observed in both proximal and distal regions. However, taking ACRs as an example, the increase of both peak number and chromatin accessibility were preferred for intergenic, rather than proximal regions (Revised Fig. S3b,c). Thus, this bias dynamic should be generated from the biological process, but not the calculation artifact.*

What is the function of H3K9me3? How much H3K9me3 and the ATAC-seq signal correlates? Authors wrote "This may be correlated with the transient expression of TEs during embryogenesis." But where are the data showing a transient expression of TEs during embryogenesis?

*Reply: Thank you for pointing out this issue. H3K9me3 is enriched in both TEs and expressed genes in plants, which function is not clear [22,23]. Based on the suggestion of this reviewer, we did further analysis between ATAC-seq and H3K9me3, as well as other histone modifications. In general, H3K9me3 was not enriched with dACRs region and was even mutually exclusive (Revised Fig. S3e). H2A.Z and H3K27ac were highly overlapped with dACRs beyond expected (Revised Fig. S3e). However, dACRs gained at DPA8 performed varying compared with consistent dACRs (Revised Fig. 2c, Fig. S3e, S3f). The dACRs that gained at DPA8 but disappear at DPA12 were termed “transient dACRs”. Intriguingly, H3K9me2/3 were relatively enriched in transient dACRs, which marked TEs and mediate heterochromatin. As expected, ‘transient dACRs’ involved a higher proportion of TEs compared with consistent dACRs, although the level is lower than genome-wide (Revised Fig. S3d).*

*We thus want to know if there is a relationship between transient dACRs and transcription of TEs or non-coding transcripts. Total RNA with removal of rRNA from wheat embryo at DPA6, 8 and 12 were sequenced (Revised Fig. S3g) (2-3 bio-reps for each stage), and the expression of coding and non-coding were calculated. As a result, there was a global increase in non-coding RNA(ncRNA) expression in DPA8 (Revised Fig. 2d), and this is correlated with the increase of dACRs number. More than 74% (n = 6,412) of non-coding genes that gained pACRs at DPA8 overlapped with TEs (Revised Fig. S3h), indicating that the transcription of non-coding genes could largely represent the transcription of TEs ). It suggests the distal chromatin accessibility increase at DPA8 may function in regulating transient transcription of TEs. Similarly, activation of TEs such as LINE is detected at the 2-cell stage of early embryos in mouse, which was highly marked by open chromatin and is essential for ZGA[24,25]. In Arabidopsis, the expression of 24-nt sRNAs reached peak at mid-embryonic stage which further promoted cell-autonomous TE silencing [26,27]. Further analysis of DNA methylome and small RNA profiling around the transition stage would give better conclusion of the functional consequence of such dACR eruptions in wheat.*

*Based on the reviewer’s suggestion, we did further analysis in Fig. 2 and Fig. S3 and have modified our description and conclusions in Result (Line 184-200) and Discussion (Line 540-554).*

3. Authors wrote "reg-ACRs were overlapped with H2A.Z (~15% overlapping), a histone variant with looser DNA wrapping than canonical H2A (Fig. 2i). Reg-dACRs with H2A.Z exhibited higher 'enhancer activity' with more accessibility and higher expression of targeted genes (Fig. 2j, k)." First I do not understand how a region that must be nucleosome free if it is accessible could show a stronger level of H2A.Z. Second, what mean for the authors enhancer activity? An accessible region could enhance or repress transcription. Third, Authors show results with H2A.Z but what about the histone modifications.

*Reply: Thank you for the comment. Not only nucleosome-free regions but also nucleosome regions could be detected by ATAC-seq (Response letter Figure1) [28,29]. For nucleosome regions, if the wrapping of DNA around the nucleosome is relatively loose, the Tn-5 insertion would be relatively easy, which could be further reflected by the high ATAC-seq signal and ATAC-seq peaks [28]. On the other hand, H2A.Z was wrapped relatively looser by DNA compared with canonical H2A [30]. Therefore, it is possible for the highly overlapping between dACRs and H2A.Z regions. In addition, the overlapping analysis between chromatin accessible regions and other histone modifications is reported in previous studies, such as the fig4b in [5] and fig1e in [31].*

*We agree that an accessible region could enhance or repress transcription. However, chromatin accessibility is positively correlated with the expression of targets in most conditions for both accessible regions that enhance or repress transcription [28,29]. For the repression case, there is a negative correlation between TFs expression and chromatin accessibility, but a positive correlation between chromatin accessibility and target expression (Response letter Figure2) [17,32]. Thus, the chromatin accessibility of the dACRs could represent the ability of those that active target genes. A dACR with high “enhancer activity” should have relatively high chromatin accessibility and target expression. This analysis strategy has been used in many previous studies [17–19,33].*

Third, Authors show results with H2A.Z but what about the histone modifications.

*Reply: We have analyzed other histone modifications according to the Reviewer’s comments in Revised Fig. 2i. H2A.Z and H3K27ac were relatively enriched for reg-dACRs.*

4. Authors wrote "Proximal and distal chromatin accessibility undergoes distinct reprogramming during embryogenesis, with different transcriptional regulation readout." I do understand the authors would like to express with this sentence.

*Reply: Thank you for pointing out this issue. We have replaced “readout” with “output” in the revision. In this section, we compared the number of peaks and function of target genes between pACRs and dACRs. The distinct reprogramming pattern between pACRs and dACRs could be exemplified by the different peak number changes, in which pACRs mainly increased at DPA2 while dACRs mainly increased at DPA8 (Fig. 2b). The burst of dACRs at DPA8 was correlated with generation of non-coding transcripts underlined, including an enriched proportion of TEs (Fig. 2c, 2d and Fig. S3 d-h). However, the pACRs at DPA8 was not significantly increased (Fig2c, FigS3b). In addition, the functional enrichment of regulated genes of pACRs and dACRs was different (Fig. 2f, 2g; Fig. S4a). Those differences indicate varied regulation weights of the two types of ACRs.*

5. I have serious doubt regarding the chromatin states performed by the authors.

First, how authors did to start with 15 and end with 5 major functional classes.

*Reply: Thanks, we understand the reviewer's concern. The five major functional classes analysis strategy referenced from [18]. In this study, the conclusions between 15-clusters and 5-clusters were highly consistent (Response letter Figure3). Plus, 15-clusters visualization was too complex to understand (Response letter Figure3). We, therefore, used the 5-clusters result as in the manuscript. Similar analysis strategies have been used in previous studies [18,34].*

Second, authors wrote "enhancer-like classes were enriched for active histone modifications such as H3K27/9ac, H3K4me1/3". It would be great that authors generate heat maps centered (i) on enhancer-like region, (ii) promoter, (iii) transcriptional unit, (IV) on polycomb and (V) on TE to show how the level of each histone modification looks. This will allow the reader to see if a correlation exists. Of course the different regions should overlap.

*Reply: Appreciate for your valuable suggestions. We have added the suggested heat map results in Revised Fig. 3b. Promoter and enhancer-like classes were enriched for active histone modifications such as H3K27ac, H3K4me3, higher chromatin accessibility, and histone variant H2A.Z.*

6. Authors wrote that "we further examined H3K4me3 and H3K27me3 co-occupied bivalent regions" but where is the experiment that proved that H3K4me3 and H3K27me3 co-occupied bivalent regions and that it is not the signal observed because of the mixture of different cell type where in one cell type the region is only marked by H3k4me3 and in another cell type the same region is marked by H3K27me3?

*Reply: We gratefully appreciate for your rigorous comment. We agree that to claim co-occupancy of H3K4me3 and H3K27me3 at bivalent nucleosome needs evidence such as ChIP-re-ChIP[35].We tried ChIP-re-ChIP-qPCR for wheat leaf tissue and found minimal 1g of fresh material was needed (Response letter Figure4), which is huge challenge for the embryos of wheat by our current dissecting methods. Actually, the main reason we used CUT&Tag instead of ChIPseq in this study is because that it requires much less input nuclei as described in the main text and method, while giving reliable result (Fig. S1a,b,c). However, there is no report for sequential CUT&Tag of different antibodies currently, to our best of knowledge. Currently, we are unable to provide such evidence for co-occupancy of H3K4me3 and H3K27me3 on the same nucleosome. In addition, the bivalent state is not the key focus of this manuscript. Therefore, we removed the analysis and statement of this part in the revision.*

7. Authors wrote "In both proximal and distal bivalent regions, we observed widespread ATAC-seq, H3K27ac, H3K4me1, H3K9me3, Pol II, and H2A.Z signals, but not transcripts and transcription-associated H3K36me3, suggesting a transcriptionally poised state (Fig. 2g and Fig. S5b)." I do not understand what authors mean by "in both proximal and distal bivalent regions". Authors talk about enhancer or ORF? If it is enhancer why looking for transcripts? If it is ORF why talking about "proximal and distal"

*Reply: Thanks for your comments, sorry for the misleading. Proximal represents the gene coding region (from 3Kb upstream of TSS to 2.5Kb downstream of TES), while distal represents the dACR at the intergenic regions. We wonder what other histone modification feature is specific for those bivalent regions. For proximal bivalent region (gene coding region), we found no transcript or transcription associated H3K36me3. For distal bivalent region, we wonder whether there might be enhancer RNA like transcript as the case of e-RNA in mammalian system [36].However, since the bivalent regions defined here are not rigorous as reviewer pointed out, and the bivalent region is not the main focus of this manuscript, we decide to remove this part.*

8. Authors wrote "H3K27me3 decreased at both up- and down-regulated genes but more obvious for up-regulated genes." How do they explain this since this mark is known to be associated with gene repression?

*Reply: Thanks, we have rephrased this sentence to make it clear (Line 279-281). H3K27me3 is known to associate with gene repression. Here, we observed a remarkable decreasing of H3K27me3 for the up-regulated gene clusters (Fig. 4b), which fits well with H3K27me3 being linked to repression. However, we still found slightly decreased H3K27me3 at down-regulated genes, which suggested that other factors may be involved for those genes’ expression as well. It is reported that loss of PRC2, writer of H3K27me3, caused genome-wide reduction of H3K27me3, but still nearly half genes’ expression is down-regulated in Arabidopsis [37].*

9. Authors wrote "Collectively, reprogramming of histone modification, particularly H3K27ac and H3K27me3, and establishing chromatin accessibility at early embryo development ensures the proper transition from maternal to zygotic process and initiation of the embryo development." While causation and correlation can exist at the same time, correlation doesn't mean causation. To prove that authors must use genetics.

*Reply: Thanks for the comments. We fully agree with reviewer that correlation doesn't mean causation. We have changed the phrase “ensures” to “correlates with” in the revision.*

*We attempted to knock down or knock out “writers” for H3K37me3 or H3K27ac, without success so far. It is technique challenge to get this work done in allohexaploid wheat. There are 9 potential H3K27me3 methyltransferase in common wheat (Response letter Figure5). CRISPR-Cas9 mediated genome editing strategy give low efficiency in hexaploid wheat [38]. For H3K27ac, giving the non-specificity of HAT or HDAC function on individual histone acetylation, it is even harder to provide genetic evidence.*

10. Authors wrote "Interestingly, TaLEC2 could bind the pACR of BBM directly (Fig. 5i)." I do not see any data showing that TaLEC2 could bind the pACR of BBM.

*Reply: Thanks for the comments. We showed that TaLEC2 could active the expression of luciferase reporter of proTaBBM::LUC, which contains the pACR fragment of TaBBM in Fig. 5i. In the revision, we have added the mutated version of proTaBBM, with disruption of potential TaLEC2 binding motifs within pACR fragment of TaBBM promoter. With such mutation, TaLEC2 could no longer active the luciferase reporter (Revised Fig. 5i). Thus it provides indirect evidence that TaLEC2 could active TaBBM via binding the motifs within pACR of TaBBM. We agree with the reviewer that this is not an evidence for directly binding of TaLEC2 to pACR of TaBBM. So we have revised the sentence as “Interestingly, TaLEC2 could active TaBBM expression via the B3 protein binding motifs within pACR of TaBBM” in lines 358-360 of the revision.*

*Reference*

*1. Hao Z, Zhang Z, Xiang D, Venglat P, Chen J, Gao P, et al. Conserved, divergent and heterochronic gene expression during Brachypodium and Arabidopsis embryo development. Plant Reprod. 2021;1–18.*

*2. Hofmann F, Schon MA, Nodine MD. The embryonic transcriptome of Arabidopsis thaliana. Plant Reprod. 2019;32:77–91.*

*3. Armenta-Medina A, Gillmor CS, Gao P, Mora-Macias J, Kochian LV, Xiang D, et al. Developmental and genomic architecture of plant embryogenesis: from model plant to crops. Plant Commun. 2021;2:100136.*

*4. Dresselhaus T, Jürgens G. Comparative Embryogenesis in Angiosperms: Activation and Patterning of Embryonic Cell Lineages. Annu Rev Plant Biol. 2021;72:641–76.*

*5. Lu Z, Marand AP, Ricci WA, Ethridge CL, Zhang X, Schmitz RJ. The prevalence, evolution and chromatin signatures of plant regulatory elements. Nat Plants. 2019;5:1250–9.*

*6. Evsikov AV, de Vries WN, Peaston AE, Radford EE, Fancher KS, Chen FH, et al. Systems biology of the 2-cell mouse embryo. Cytogenet Genome Res. 2004;105:240–50.*

*7. Becker JS, Nicetto D, Zaret KS. H3K9me3-Dependent Heterochromatin: Barrier to Cell Fate Changes. Trends Genet. 2016;32:29–41.*

*8. Zhang W, Chen F, Chen R, Xie D, Yang J, Zhao X, et al. Zscan4c activates endogenous retrovirus MERVL and cleavage embryo genes. Nucleic Acids Res. 2019;47:8485–501.*

*9. Guo M, Zhang Y, Zhou J, Bi Y, Xu J, Xu C, et al. Precise temporal regulation of Dux is important for embryo development. Cell Res. 2019;29:956–9.*

*10. Xu R, Li C, Liu X, Gao S. Insights into epigenetic patterns in mammalian early embryos. Protein Cell. 2021;12:7–28.*

*11. Liu X, Wang C, Liu W, Li J, Li C, Kou X, et al. Distinct features of H3K4me3 and H3K27me3 chromatin domains in pre-implantation embryos. Nature. 2016;537:558–62.*

*12. Dahl JA, Jung I, Aanes H, Greggains GD, Manaf A, Lerdrup M, et al. Broad histone H3K4me3 domains in mouse oocytes modulate maternal-to-zygotic transition. Nature. 2016;537:548–52.*

*13. Xia W, Xie W. Rebooting the Epigenomes during Mammalian Early Embryogenesis. Stem Cell Reports. 2020;15:1158–75.*

*14. Zheng H, Huang B, Zhang B, Xiang Y, Du Z, Xu Q, et al. Resetting Epigenetic Memory by Reprogramming of Histone Modifications in Mammals. Mol Cell. 2016;63:1066–79.*

*15. Hensel G. Genetic transformation of Triticeae cereals - Summary of almost three-decade’s development. Biotechnol Adv. 2020;40:107484.*

*16. Imani J, Kogel K-H. Plant Transformation Techniques: Agrobacterium- and Microparticle-Mediated Gene Transfer in Cereal Plants. Methods Mol Biol. 2020;2124:281–94.*

*17. Corces MR, Granja JM, Shams S, Louie BH, Seoane JA, Zhou W, et al. The chromatin accessibility landscape of primary human cancers. Science. 2018;362:eaav1898.*

*18. Gorkin DU, Barozzi I, Zhao Y, Zhang Y, Huang H, Lee AY, et al. An atlas of dynamic chromatin landscapes in mouse fetal development. Nature. 2020;583:744–51.*

*19. Trevino AE, Sinnott-Armstrong N, Andersen J, Yoon S-J, Huber N, Pritchard JK, et al. Chromatin accessibility dynamics in a model of human forebrain development. Science. 2020;367:eaay1645.*

*20. Li Z, Wang M, Lin K, Xie Y, Guo J, Ye L, et al. The bread wheat epigenomic map reveals distinct chromatin architectural and evolutionary features of functional genetic elements. Genome Biol. 2019;20:139.*

*21. IWGSC, Appels R, Eversole K, Stein N, Feuillet C, Keller B, et al. Shifting the limits in wheat research and breeding using a fully annotated reference genome. Science. American Association for the Advancement of Science; 2018;361:eaar7191.*

*22. Roudier F, Teixeira FK, Colot V. Chromatin indexing in Arabidopsis: an epigenomic tale of tails and more. Trends Genet. 2009;25:511–7.*

*23. Xu L, Jiang H. Writing and Reading Histone H3 Lysine 9 Methylation in Arabidopsis. Front Plant Sci. 2020;11:452.*

*24. Jachowicz JW, Bing X, Pontabry J, Bošković A, Rando OJ, Torres-Padilla M-E. LINE-1 activation after fertilization regulates global chromatin accessibility in the early mouse embryo. Nat Genet. 2017;49:1502–10.*

*25. Percharde M, Lin C-J, Yin Y, Guan J, Peixoto GA, Bulut-Karslioglu A, et al. A LINE1-Nucleolin Partnership Regulates Early Development and ESC Identity. Cell. 2018;174:391-405.e19.*

*26. Papareddy RK, Páldi K, Paulraj S, Kao P, Lutzmayer S, Nodine MD. Chromatin regulates expression of small RNAs to help maintain transposon methylome homeostasis in Arabidopsis. Genome Biol. 2020;21:251.*

*27. Li C, Gent JI, Xu H, Fu H, Russell SD, Sundaresan V. Resetting of the 24-nt siRNA landscape in rice zygotes. Genome Res. 2022;32:309–23.*

*28. Buenrostro JD, Wu B, Chang HY, Greenleaf WJ. ATAC-seq: A Method for Assaying Chromatin Accessibility Genome-Wide. Curr Protoc Mol Biol. 2015;109:21.29.1-21.29.9.*

*29. Tsompana M, Buck MJ. Chromatin accessibility: a window into the genome. Epigenetics Chromatin. 2014;7:33.*

*30. Giaimo BD, Ferrante F, Herchenröther A, Hake SB, Borggrefe T. The histone variant H2A.Z in gene regulation. Epigenetics Chromatin. 2019;12:37.*

*31. Murphy KE, Meng FW, Makowski CE, Murphy PJ. Genome-wide chromatin accessibility is restricted by ANP32E. Nat Commun. 2020;11:5063.*

*32. Berest I, Arnold C, Reyes-Palomares A, Palla G, Rasmussen KD, Giles H, et al. Quantification of Differential Transcription Factor Activity and Multiomics-Based Classification into Activators and Repressors: diffTF. Cell Rep. 2019;29:3147-3159.e12.*

*33. Amiri A, Coppola G, Scuderi S, Wu F, Roychowdhury T, Liu F, et al. Transcriptome and epigenome landscape of human cortical development modeled in organoids. Science. 2018;362:eaat6720.*

*34. Wu L-Y, Shang G-D, Wang F-X, Gao J, Wan M-C, Xu Z-G, et al. Dynamic chromatin state profiling reveals regulatory roles of auxin and cytokinin in shoot regeneration. Developmental Cell. 2022;*

*35. Desvoyes B, Sequeira-Mendes J, Vergara Z, Madeira S, Gutierrez C. Sequential ChIP Protocol for Profiling Bivalent Epigenetic Modifications (ReChIP). Methods Mol Biol. 2018;1675:83–97.*

*36. Ye R, Cao C, Xue Y. Enhancer RNA: biogenesis, function, and regulation. Essays Biochem. 2020;64:883–94.*

*37. Bouyer D, Roudier F, Heese M, Andersen ED, Gey D, Nowack MK, et al. Polycomb repressive complex 2 controls the embryo-to-seedling phase transition. PLoS Genet. 2011;7:e1002014.*

*38. Ansari WA, Chandanshive SU, Bhatt V, Nadaf AB, Vats S, Katara JL, et al. Genome Editing in Cereals: Approaches, Applications and Challenges. Int J Mol Sci. 2020;21:E4040.*

**Second round of review**

**Reviewer 2**

In their revised version authors improved their manuscript. However I still have some major concerns about it. I strongly believe that this article may be of interest to the plant community; but I have concerned that the general principles may not be novel enough for a general audience.

Authors argue that one a major point raised in their paper is the fact that they "revealed important features of epigenomic dynamic that distinguish the early embryogenesis of wheat from that of mouse or human." Embryogenesis in plant and animal has nothing in common and is not comparable.

Authors in their response letter that "For dACRs, all genes within a 0.5-Mb region around a dACR were analyzed, and the ones that exhibited a significant correlation were selected as the targets (Fig. S4c)." This cannot be done this way. A regulatory element can be an enhancer but also a repressor sequence then if the authors assign all the time dACRs to a gene that exhibited a significant correlation within a 0.5-Mb region around it does not make sense for me. I totally disagree with this way of assigning a regulatory element to a gene. This is totally biased.

In addition the hi-C used were not done on the same tissue; I agree that it could be used as a good proxy but it is not enough for a publication it a top journal in the field.

Authors wrote in their response about H3K9me3 "H3K9me3 is enriched in both TEs and expressed genes in plants, which function is not clear [22,23]" citing one paper using ChIP on ChIP and a review citing the paper that used the ChIP on ChIP. In fact almost no ChIP-seq are publicly available for H3K9me3 in plant and it is not clear at all if this mark is even present in plant. Authors showed a screenshot on figure 1A in which the same gene at almost the same position seems to display an H3K9me3 signal and H3K9me2 which is not possible the K9 can only be either di methylated or methylated but not both.

Authors wrote that "it is possible for the highly overlapping between dACRs and H2A.Z regions. "

I disagree with that as you can see in the following papers (https://doi.org/10.1038/s41467-020-18821-x figure 1A ; https://doi.org/10.1093/nar/gkaa157 Figure 2C and 2D) the H2AZ signal does not overlap with the ATAC-seq signal.

I have a concern about the H2AZ antibody. What control was made to prove that it does not cross react and recognize specifically H2AZ? This control is mandatory. By the way none of the antibodies used are described.

Authors did not answer my comment about "enhancer-like classes" that "were enriched for active histone modifications such as H3K27/9ac, H3K4me1/3". It would be great that authors generate heat maps centered (i) on enhancer-like region, (ii) promoter, (iii) transcriptional unit, (IV) on polycomb and (V) on TE to show how the level of each histone modification looks. This will allow the reader to see if a correlation exists. Of course the different regions should overlap. What they did is not what I asked for.

**Reviewer 3**

Due to difficulitiy of tissue isolation and technical limitation, the epigenetic mechanism of embryogenes remain largely elusive in crop. Zhao et al integrated diverse omics data to explore chromatin and transcriptomic dynamics during eight embryogenesis stages (DPA0-22) of wheat. Interestingly, they found a sharp burst of distal ACRs at DPA8 but quick loss of these ACRs at DPA12, which may be correlated with transient expression of TEs during embryogenesis. They also revealed remodeling of histone modifications during different stages of embryogenesis. This impressive study provide comprehensive epigenomic landscapes during wheat embryogenesis and will be an invaluable resource for mechanistic analysis of the epigenomic regulation of crop embryogenesis. Overall, the conclusions are novel and the addressed scientific questions are interesting. This study will be of broad interest to plant reproductive biology and crop breeding. I have a few minor comments below.

1 Line 161, there should be a blank between number and 'Kb', like '125 Mb' in line 166. Also check other sentences to keep the format consistent in whole manuscript.

2 Line 179, a blank is missed between 'distal (d)' and 'ACRs'.

3 They found chromatin state pattern at DPA0 was distinguished from other stages (Fig. 3e), however, I can't understand how they get this conclusion from Fig.3e.

4 H3K27me3 and H3K9me3 exhibited higher variation among sub-genomes than active histone marks such as H3K27ac, H3K4me3, and histone variant H2A.Z (Fig.3g). Any statistic test?

5 A typo in figure legend of Fig. 7e. "Statistic test??"

**Authors Response**

**Point-by-point responses to the reviewers’ comments:**

Reviewer #2

1. In their revised version authors improved their manuscript. However I still have some major concerns about it. I strongly believe that this article may be of interest to the plant community; but I have concerned that the general principles may not be novel enough for a general audience.

*Reply: Thanks for the reviewer’s critical comment, which makes us to rethink about our study at a broader point of view. Embryogenesis represents the beginning of life cycle for both animals and flower plants. This is valued for animals as well as plants.*

*Though there is numerous studies on transcriptome and gene functional analysis in plant embryogenesis, this is the first chromatin landscape dynamic analysis dataset presented in plant kingdom, even for the model plant Arabidopsis. So we would expect many plant scientists from different research fields such as crops genetics, breeding, plant epigenetics, reproductive development, and bioinformatics would feel interest to read. Considering about the size of the plant research community, we don’t think this could not be considered as a ‘general’ audience.*

*For scientists in animal research community, the fact that many studies have been conducted to understand the epigenomic reprogramming at early embryogenesis in human and mouse indicates the importance for comparison between species for conservation and diversification analysis. So we would expect some of them may be curious about how different and similar for epigenetic regulated embryogenesis between species from two kingdoms separated for 1000 mya. There are numerous examples for animal and plant scientists have common research interests, such as hormone pathways, environmental stimuli sensing, and immunity systems. We do think both community could learn and be inspired by reading information from two distinct kingdoms.*

*Wheat is hexaploid species, different from mouse and human with diploid genome. This could be a novel angle for looking at transcriptome and epigenomic dynamic during embryogenesis for homeologs from different sub-genomes. Indeed, there is generally difference for active and repressive histone marks dynamics among different subgenome.*

*Thus, we think our finding may be of interest for a broad general audience.*

2. Authors argue that one a major point raised in their paper is the fact that they "revealed important features of epigenomic dynamic that distinguish the early embryogenesis of wheat from that of mouse or human." Embryogenesis in plant and animal has nothing in common and is not comparable.

*Reply: We agree that there are huge morphological differences between plant and animal embryonic development, especially at late embryonic stages, but we can’t agree with the reviewer’s statement of “Embryogenesis in plant and animal has nothing in common and is not comparable".*

*Firstly, the cell fate determination during embryogenesis is similar in plants and animals: following fertilization, two terminally differentiated gametes were fused into a totipotent zygote, which would undergo cellular division followed by differentiation [1–3]. Secondly, during the maternal-to-zygotic transition (MZT) process, both animal and plant zygote would undergo maternal genome silencing and zygotic activation [4–8]. Animal and plant have independently evolved over 1000 mya, but the molecular mechanisms during MZT are still comparable. Zhao and colleagues showed that “plant MZT is similar to that in animals, showing a typical two-step process” [6]. Thirdly, There are many comparisons between the parental contribution in early embryogenesis in plants and animals [7–15]. In the previous study, the authors declared that " flowering plants evolved strategies to regulate early embryogenesis that is similar to those described in animals"[7]. While in other studies, the authors stated that " in contrast to early animal embryogenesis, early plant embryogenesis is mostly under zygotic control"[8], and " In contrast to animals, the plant zygotic genome is activated shortly after fertilization. The maternal and paternal genomes contribute equally to the transcriptome of early embryos"[12]. Fourthly, there are studies to compare the timing of ZGA between plant and animal and concluded that "In animals, early embryo divisions occur within a constant total volume, utilizing maternally deposited transcripts until ZGA, whereas plant embryonic volume increases continuously after fertilization"[14]. Fifth, there are homologs may play the same roles in animals and plants, for example in maize "ZmMAB1 may also function like its animal homolog, the key ACD regulator MEL-26, a factor required for embryogenic morphogenesis that regulates the formation of mitotic spindles in the early embryo"[15].*

*These indicate that it’s comparable between animal and plant at least for the process of MZT and ZGA with both conservation and divergence. In this study, we mainly compared the epigenetics dynamic between animal and plant during MZT and ZGA process, a stage similar as the aforementioned studies have focused on. We indeed find similar histone modification reprogramming pattern in wheat compared with animal, but differences in details of the kinetics for individual histone mark. Thus, we have modified our conclusion in the result and discussion of the 2nd revision manuscript, with emphasis of both conservation and diversification.*

3. Authors in their response letter that "For dACRs, all genes within a 0.5-Mb region around a dACR were analyzed, and the ones that exhibited a significant correlation were selected as the targets (Fig. S4c)." This cannot be done this way. A regulatory element can be an enhancer but also a repressor sequence then if the authors assign all the time dACRs to a gene that exhibited a significant correlation within a 0.5-Mb region around it does not make sense for me. I totally disagree with this way of assigning a regulatory element to a gene. This is totally biased.

*Reply: Thanks for your comments. We do agree that dACRs may function as enhancers or silencers. In this study, we only focused on enhancers rather than silencers, that is why we used a correlation strategy for positive regulatory dACRs discovering, which were named as reg-dACRs.*

*The correlation strategy for distal enhancer-genes assignment was used in many studies and has been validated by multiple experiments. The followings are brief citations and description of such analysis in these articles:*

*(1) In ‘The chromatin accessibility landscape of primary human cancers’ (2018, Science), the authors showed that “we restricted the length scale of this analysis to 500 kb to avoid spurious predictions. Using a conservative FDR cutoff of 0.01, we identified 81,323 unique links between distal ATAC-seq peaks and genes” in the “Linking of DNA regulatory elements to genes predicts interactions relevant to cancer biology” section [16]. Plus, the accuracy of the peak-to-gene links approach has been fully demonstrated using CRISPR interference (CRISPRi) and other strategies.*

*(2) In “An atlas of dynamic chromatin landscapes in mouse fetal development” (2020, Nature), the authors showed that “to predict enhancer target genes using the correlation between gene expression (as measured by RNA-seq) and H3K27ac enrichment at candidate enhancers within the same TAD” [17]. The threshold of this paper was described that “Only those putative enhancers showing a P value ≤ 0.05 (for both strategies) and an SCC ≥ 0.25 were retained”. The authors showed that “This correlation-based map predicts experimentally determined enhancer–gene interactions with higher accuracy than assigning an enhancer to the nearest gene”. In addition, the feasibility of the correlation strategy was validated by the eQTL and the in vivo method. In mouse, H3K27ac could characterize enhancers and the author used the H3K27ac-RNA correlation to find enhancer-target linkers. In plant, however, enhancers are best characterized by chromatin accessibility, rather than a single histone modification [18], and we thus used the ATAC-RNA correlation to find enhancer-target links.*

*(3) In “Chromatin accessibility dynamics in a model of human forebrain development” (2020, Science), the authors showed that “We adapted an approach to discover putative enhancer-gene linkages on the basis of the coordination of accessibility and gene expression across cell lineages. For each gene, we then computed correlations across samples between gene expression and accessible peaks within a 500-kb window, taking into account the distribution of spurious correlations” [19]. This paper used the correlation strategy and 500-kb distance.*

*(4) In “Chromatin and gene-regulatory dynamics of the developing human cerebral cortex at single-cell resolution” (2021, cell), the authors showed that “We then obtained candidate peak-gene pairs by associating peaks with a genomic distance between 1 and 250 kb to the TSS of protein coding and lincRNA genes to the respective genes. For each candidate peak-gene pair we computed the Pearson correlation coefficient of CPM-normalized counts of accessibility and gene expression data and computed FDR-adjusted P-values for these coefficients based on their t-statistic. We defined a set of 64,878 high-confidence peak-to-gene links by only retaining pairs with |PCC|>0.4 and FDR-adjusted P-value < 0.05”[20]. Although single-cell data was used in this paper, almost the same correlation strategy was applied.*

*In sum, the correlation strategies were widely used in previous studies, although the data type and distance between distal peaks to genes were varied in different species.*

4. In addition the hi-C used were not done on the same tissue; I agree that it could be used as a good proxy but it is not enough for a publication it a top journal in the field.

*Reply: Thanks for your suggestion and comment. No Hi-C data was available currently for wheat embryo tissue as far as we know. It is another great challenge to perform Hi-C experiments in wheat embryo due to the large amount of input nuclei needed for reaching the resolution of detection of chromatin loop, roughly about 2-5x10e7nuclei for one sample according to recent publication of HiC assay in plant [21–24]. We could get maximal 1-2x 10e6 intact nuclei from DPA6-22 developing wheat embryo, even less amount for DPA0-4. That is also the main reason why we choose CUT&Tag for histone modification marks profiling instead of ChIPseq.*

*Even used Hi-C data from a different tissue, we get statistically enriched reg-dACR with loop anchor regions (Fig. S4d). One would expect an even higher overlap when the same tissue is used to generate HiC data, which would further prove the accuracy of the reg-dACR-genes assignment.*

5. Authors wrote in their response about H3K9me3 "H3K9me3 is enriched in both TEs and expressed genes in plants, which function is not clear [22,23]" citing one paper using ChIP on ChIP and a review citing the paper that used the ChIP on ChIP. In fact almost no ChIP-seq are publicly available for H3K9me3 in plant and it is not clear at all if this mark is even present in plant.

*Reply: Thanks for the reviewer’s carefully checking, sorry for the meaningless citations we used in the last version of response letter. Indeed, both H3K9me2 and H3K9me3 ChIP-seq data have been generated in plant “Dynamic chromatin state profiling reveals regulatory roles of auxin and cytokinein in shoot regeneration” [25]. The antibody they used for H3K9me3 is “Abcam AB_306848(ab8898)”, the same as we used in this study. In the 2nd revision submitted currently, we have added all information regarding the antibodies in Table S19.*

6. Authors showed a screenshot on figure 1A in which the same gene at almost the same position seems to display an H3K9me3 signal and H3K9me2 which is not possible the K9 can only be either di methylated or methylated but not both.

*Reply: Thanks for your critical comment about H3K9me2 and H3K9me3 signal from a similar region shown in Fig. 1A. Our CUT&Tag is from bulk cell population, the peak intensity is from the sum of population of cells. We currently can’t get single cell resolution, so the peak of H3K9me2 and H3K9me3 from similar region of some specific genome loci as shown in Fig. 1A, doesn’t mean in the same single cell both H3K9me2 and H3K9me3 co-exist. As well, the relative intensity is significantly different from each other.*

*The other argument we want to make is that only the TSS region of case-shown gene looks “similar” for H3K9me2 and H3K9me3 in Fig.1A. The profiles of two histone marks are different if one looks at the whole genic region in Fig. 1A. While we zoom out to look at a broader region, these two histone marks are totally different, as shown below. In addition, the overall distribution patter of these two histone mark is different (Fig. S2a). So it is unlikely the signals of these two histone marks are the same.*

7. Authors wrote that "it is possible for the highly overlapping between dACRs and H2A.Z regions. "I disagree with that as you can see in the following papers (https://doi.org/10.1038/s41467-020-18821-x figure 1A ; https://doi.org/10.1093/nar/gkaa157 Figure 2C and 2D) the H2AZ signal does not overlap with the ATAC-seq signal。

*Reply: Thanks for your comments and references. In theory, H2A.Z signal is on nucleosomes and ATAC-seq signal is mainly on nuclear free regions. There shouldn't be any overlap between H2A.Z and chromatin accessibility. The "overlapping " here means the intersection of peaks in bioinformatics analysis just like the Fig.1e in [26] (the publication reviewer mentioned), and more than 1 bp intersection was defined as "overlap". We realized that the "overlap" may be misleading and changed the statement in the revised manuscript Line 228 that "reg-dACRs were mostly associated with H2A.Z" and clarify the term of ‘overlap’ in the legends of Fig. 2j and 2k.*

*However, we believe the general conclusion about correlation between reg-dACRs and flanking H2A.Z intensity is right with supportive evidences from previous publications and our current study.*

*For instance, in publication entitled “Genome-wide chromatin accessibility is restricted by ANP32E” (https://doi.org/10.1038/s41467-020-18821-x) [26], the authors showed that “there was considerable overlap of H3K27ac, H2A.Z, and chromatin accessibility” and “These results indicate that H2A.Z is consistently correlated with chromatin accessibility”. Both the analysis method and conclusions are similar between our study and this paper.*

*The analysis method in Fig. 2j of our manuscript and the statistics in revised Fig. 2i is the same as the Fig. 1e in previous publication [26].*

*In “H2A.Z is dispensable for both basal and activated transcription in post-mitotic mouse muscles” (https://doi.org/10.1093/nar/gkaa157) [27], the authors showed that “chromatin was the most accessible between the +1 and −1 nucleosomes surrounding the TSS(Fig. 2D)” while H2A.Z was most enriched at the +1 and −1 nucleosomes (Fig. 2D). This indicates that there is a correlation between chromatin accessibility and H2A.Z at flanking nucleosomes. This is consistent with our finding.*

8. I have a concern about the H2AZ antibody. What control was made to prove that it does not cross react and recognize specifically H2AZ? This control is mandatory. By the way none of the antibodies used are described.

*Reply: Thanks for your comment and suggestions. There are six H2A.Z coding genes in wheat. It is impossible for generating knock-out mutant. As well, currently there is no H2A.Z-Tag transgenic wheat line in the research community, so it is hard to get a proper control in wheat per se.*

*The antibody for H2A.Z in this study is a gift from Prof. Roger B Deal lab in the University of Emory, USA. It has been used for ChIP-seq and ChIP-qPCR in many publications in Arabidopsis [28–30], including our previous publication[31].This indicates that the H2A.Z antibody used in this study could capture H2A.Z specifically in Arabidopsis. The sequence of H2A.Z coding genes in wheat are quite similar to those in Arabidopsis compared with H2A coding genes in wheat (Fig below). So we believed that the H2A.Z antibody works for wheat as well. We are sorry for missing the antibody information. We have added the information to Table S19 in the 2nd revision.*

9. Authors did not answer my comment about "enhancer-like classes" that "were enriched for active histone modifications such as H3K27/9ac, H3K4me1/3". It would be great that authors generate heat maps centered (i) on enhancer-like region, (ii) promoter, (iii) transcriptional unit, (IV) on polycomb and (V) on TE to show how the level of each histone modification looks. This will allow the reader to see if a correlation exists. Of course the different regions should overlap. What they did is not what I asked for.

*Reply: Thanks for your suggestion. Sorry we didn’t get your meaning last time. Now we have added this part of analysis as you suggested in Fig. S5a in the 2nd revision.*

Reviewer #3:

Due to difficulitiy of tissue isolation and technical limitation, the epigenetic mechanism of embryogenes remain largely elusive in crop. Zhao et al integrated diverse omics data to explore chromatin and transcriptomic dynamics during eight embryogenesis stages (DPA0-22) of wheat. Interestingly, they found a sharp burst of distal ACRs at DPA8 but quick loss of these ACRs at DPA12, which may be correlated with transient expression of TEs during embryogenesis. They also revealed remodeling of histone modifications during different stages of embryogenesis. This impressive study provide comprehensive epigenomic landscapes during wheat embryogenesis and will be an invaluable resource for mechanistic analysis of the epigenomic regulation of crop embryogenesis. Overall, the conclusions are novel and the addressed scientific questions are interesting. This study will be of broad interest to plant reproductive biology and crop breeding. I have a few minor comments below.

*Reply: Thanks for your positive comments on our work.*

1 Line 161, there should be a blank between number and 'Kb', like '125 Mb' in line 166. Also check other sentences to keep the format consistent in whole manuscript.

*Reply: Thanks for the comments. We have gone through the paper and corrected this mistake in revised manuscript.*

2 Line 179, a blank is missed between 'distal (d)' and 'ACRs'.

*Reply: Thanks, we have corrected this mistake on Line 179.*

3 They found chromatin state pattern at DPA0 was distinguished from other stages (Fig. 3e), however, I can't understand how they get this conclusion from Fig.3e.

*Reply: Thanks for your careful check. In Fig. 3e, there was more than 20% percentage of genome coverage changed its chromatin state in DPA2/4/8 compared with DPA0, while there was less fraction of chromatin state changed in the comparison between DPA4 and DPA2, between DPA8 and DPA2, and between DPA8 and DPA4. This result indicated that there was more different between DPA0 and other stages.*

4 H3K27me3 and H3K9me3 exhibited higher variation among sub-genomes than active histone marks such as H3K27ac, H3K4me3, and histone variant H2A.Z (Fig.3g). Any statistic test?

*Reply: Thanks for the suggestion. We have added the statistic test in Fig. S5e of the revised manuscript. The absolute values of the ratio among subgenomes in H3K27me3 and H3K9me3 were significantly higher than those in H2A.Z, H3K27ac and H3K4me3. This indicated that H3K27me3 and H3K9me3 exhibited higher variation than H3K27ac, H3K4me3 and H2A.Z. LSD test was used for the statistic test.*

5 A typo in figure legend of Fig. 7e. "Statistic test??"

*Reply: Thanks, we have corrected this typo and added related statistic test information in the legends of Fig. 7e the revised manuscript.*

*References*

*1. Armenta-Medina A, Gillmor CS, Gao P, Mora-Macias J, Kochian LV, Xiang D, et al. Developmental and genomic architecture of plant embryogenesis: from model plant to crops. Plant Commun. 2021;2:100136.*

*2. Dresselhaus T, Jürgens G. Comparative Embryogenesis in Angiosperms: Activation and Patterning of Embryonic Cell Lineages. Annu Rev Plant Biol. 2021;72:641–76.*

*3. Gerri C, Menchero S, Mahadevaiah SK, Turner JMA, Niakan KK. Human Embryogenesis: A Comparative Perspective. Annu Rev Cell Dev Biol. 2020;36:411–40.*

*4. Xu R, Li C, Liu X, Gao S. Insights into epigenetic patterns in mammalian early embryos. Protein Cell. 2021;12:7–28.*

*5. Xia W, Xie W. Rebooting the Epigenomes during Mammalian Early Embryogenesis. Stem Cell Reports. 2020;15:1158–75.*

*6. Zhao P, Zhou X, Shen K, Liu Z, Cheng T, Liu D, et al. Two-Step Maternal-to-Zygotic Transition with Two-Phase Parental Genome Contributions. Dev Cell. 2019;49:882-893.e5.*

*7. Autran D, Baroux C, Raissig MT, Lenormand T, Wittig M, Grob S, et al. Maternal epigenetic pathways control parental contributions to Arabidopsis early embryogenesis. Cell. 2011;145:707–19.*

*8. Nodine MD, Bartel DP. Maternal and paternal genomes contribute equally to the transcriptome of early plant embryos. Nature. 2012;482:94–7.*

*9. Schon MA, Nodine MD. Widespread Contamination of Arabidopsis Embryo and Endosperm Transcriptome Data Sets. Plant Cell. 2017;29:608–17.*

*10. Baroux C, Autran D, Raissig MT, Grimanelli D, Grossniklaus U. Parental contributions to the transcriptome of early plant embryos. Curr Opin Genet Dev. 2013;23:72–4.*

*11. Del Toro-De León G, Lepe-Soltero D, Gillmor CS. Zygotic genome activation in isogenic and hybrid plant embryos. Curr Opin Plant Biol. 2016;29:148–53.*

*12. Zhao P, Zhou X, Zheng Y, Ren Y, Sun M-X. Equal parental contribution to the transcriptome is not equal control of embryogenesis. Nat Plants. 2020;6:1354–64.*

*13. Shi C, Luo P, Du Y-T, Chen H, Huang X, Cheng T-H, et al. Maternal control of suspensor programmed cell death via gibberellin signaling. Nat Commun. Nature Publishing Group; 2019;10:3484.*

*14. Anderson SN, Johnson CS, Chesnut J, Jones DS, Khanday I, Woodhouse M, et al. The Zygotic Transition Is Initiated in Unicellular Plant Zygotes with Asymmetric Activation of Parental Genomes. Dev Cell. 2017;43:349-358.e4.*

*15. Chen J, Strieder N, Krohn NG, Cyprys P, Sprunck S, Engelmann JC, et al. Zygotic Genome Activation Occurs Shortly after Fertilization in Maize. Plant Cell. 2017;29:2106–25.*

*16. Corces MR, Granja JM, Shams S, Louie BH, Seoane JA, Zhou W, et al. The chromatin accessibility landscape of primary human cancers. Science. 2018;362:eaav1898.*

*17. Gorkin DU, Barozzi I, Zhao Y, Zhang Y, Huang H, Lee AY, et al. An atlas of dynamic chromatin landscapes in mouse fetal development. Nature. 2020;583:744–51.*

*18. Yan W, Chen D, Schumacher J, Durantini D, Engelhorn J, Chen M, et al. Dynamic control of enhancer activity drives stage-specific gene expression during flower morphogenesis. Nat Commun. 2019;10:1705.*

*19. Trevino AE, Sinnott-Armstrong N, Andersen J, Yoon S-J, Huber N, Pritchard JK, et al. Chromatin accessibility dynamics in a model of human forebrain development. Science. 2020;367:eaay1645.*

*20. Trevino AE, Müller F, Andersen J, Sundaram L, Kathiria A, Shcherbina A, et al. Chromatin and gene-regulatory dynamics of the developing human cerebral cortex at single-cell resolution. Cell. Elsevier; 2021;184:5053-5069.e23.*

*21. Concia L, Veluchamy A, Ramirez-Prado JS, Martin-Ramirez A, Huang Y, Perez M, et al. Wheat chromatin architecture is organized in genome territories and transcription factories. Genome Biology. 2020;21:104.*

*22. Sun L, Jing Y, Liu X, Li Q, Xue Z, Cheng Z, et al. Heat stress-induced transposon activation correlates with 3D chromatin organization rearrangement in Arabidopsis. Nat Commun. 2020;11:1886.*

*23. Moissiard G, Cokus SJ, Cary J, Feng S, Billi AC, Stroud H, et al. MORC family ATPases required for heterochromatin condensation and gene silencing. Science. 2012;336:1448–51.*

*24. Yang T, Wang D, Tian G, Sun L, Yang M, Yin X, et al. Chromatin remodeling complexes regulate genome architecture in Arabidopsis. The Plant Cell. 2022;34:2638–51.*

*25. Wu L-Y, Shang G-D, Wang F-X, Gao J, Wan M-C, Xu Z-G, et al. Dynamic chromatin state profiling reveals regulatory roles of auxin and cytokinin in shoot regeneration. Developmental Cell. 2022;*

*26. Murphy KE, Meng FW, Makowski CE, Murphy PJ. Genome-wide chromatin accessibility is restricted by ANP32E. Nat Commun. 2020;11:5063.*

*27. Belotti E, Lacoste N, Simonet T, Papin C, Padmanabhan K, Scionti I, et al. H2A.Z is dispensable for both basal and activated transcription in post-mitotic mouse muscles. Nucleic Acids Research. 2020;48:4601–13.*

*28. Deal RB, Topp CN, McKinney EC, Meagher RB. Repression of Flowering in Arabidopsis Requires Activation of FLOWERING LOCUS C Expression by the Histone Variant H2A.Z. The Plant Cell. 2007;19:74–83.*

*29. Torres ES, Deal RB. The histone variant H2A.Z and chromatin remodeler BRAHMA act coordinately and antagonistically to regulate transcription and nucleosome dynamics in Arabidopsis. The Plant Journal. 2019;99:144–62.*

*30. Sijacic P, Holder DH, Bajic M, Deal RB. Methyl-CpG-binding domain 9 (MBD9) is required for H2A.Z incorporation into chromatin at a subset of H2A.Z-enriched regions in the Arabidopsis genome. PLOS Genetics. Public Library of Science; 2019;15:e1008326.*

*31. Lee U-S, Bieluszewski T, Xiao J, Yamaguchi A, Wagner D. H2A.Z contributes to trithorax activity at the AGAMOUS locus. Molecular Plant. 2022;15:207–10.*

**Third round of review**

**Reviewer 2**

In their revised version authors improved their manuscript. However, I still have some concerns about it.

One of my previous comments was:

“Authors in their response letter that "For dACRs, all genes within a 0.5-Mb region around a dACR were analyzed, and the ones that exhibited a significant correlation were selected as the targets (Fig. S4c)." This cannot be done this way. A regulatory element can be an enhancer but also a repressor sequence then if the authors assign all the time dACRs to a gene that exhibited a significant correlation within a 0.5-Mb region around it does not make sense for me. I totally disagree with this way of assigning a regulatory element to a gene. This is totally biased.”

The response from authors is:

“Thanks for your comments. We do agree that dACRs may function as enhancers or silencers. In this study, we only focused on enhancers rather than silencers, that is why we used a correlation strategy for positive regulatory dACRs discovering, which were named as reg-dACRs.

The correlation strategy for distal enhancer-genes assignment was used in many studies and has been validated by multiple experiments. The followings are brief citations and description of such analysis in these articles:

The paper cited by the authors cannot be used to prove that what they did is relevant.

(i) For the first paper cited authors wrote “The breadth and depth of this sequencing study enabled a robust association of ATAC-seq peaks with the genes that they are predicted to regulate. To do this, we implemented a strategy based on the correlation of ATAC-seq accessibility and gene expression across all samples (Fig. 5A, N=373 with matched RNA-seq and ATAC-seq). Because promoter capture Hi-C data suggested that >75% of three-dimensional (3D) promoter-based interactions occur within a 500-kbp distance (38), we restricted the length scale of this analysis to 500 kbp to avoid spurious predictions.” This means that (i) they used N=373 samples and also that they did not choose the distance randomly but “Because promoter capture Hi-C data suggested that >75% of three-dimensional (3D) promoter-based interactions occur within a 500-kbp distance”. The number of sample and the way they choose the distance makes this study and the revised version not comparable.

(ii) For the second one as authors highlight it “to predict enhancer target genes using the correlation between gene expression (as measured by RNA-seq) and H3K27ac enrichment at candidate enhancers within the same TAD” It is written within the same TAD meaning that yhey have interaction data to support this interaction. Again this is not what authors did in this paper.

(iii) For the third one they “ collected 117 ATAC-seq (assay for transposase-accessible chromatin with high-throughput sequencing) samples and 54 RNA-sequencing (RNA-seq) samples from hCSs and hSSs derived from 7 hiPS cell lines (tables S1 and S2)” and used the method presented in the first paper cited in (i). For the same reason it is not comparable.

(iv) For the last paper it is single cell data that where used and then of course it is also no comparable to what authors did in this manuscript.

To my view the best is tone down their conclusion and phrase it more cautiously or even better remove it since this is not a major point or conclusion in the paper.

Another previous comment was:

“Authors wrote in their response about H3K9me3 "H3K9me3 is enriched in both TEs and expressed genes in plants, which function is not clear [22,23]" citing one paper using ChIP on ChIP and a review citing the paper that used the ChIP on ChIP. In fact almost no ChIP-seq are publicly available for H3K9me3 in plant and it is not clear at all if this mark is even present in plant.”

The response from authors is:

Thanks for the reviewer’s carefully checking, sorry for the meaningless citations we used in the last version of response letter. Indeed, both H3K9me2 and H3K9me3 ChIP- seq data have been generated in plant “Dynamic chromatin state profiling reveals regulatory roles of auxin and cytokinein in shoot regeneration” [25]. The antibody they used for H3K9me3 is “Abcam AB_306848(ab8898)”, the same as we used in this study. In the 2nd revision submitted currently, we have added all information regarding the antibodies in Table S19.

I have an issue with this antibody. As you can read it from the abcam web page (https://www.abcam.com/histone-h3-tri-methyl-k9-antibody-chip-grade-ab8898.html) . The supplier wrote about the specificity that “Histone H3 (tri methyl K9) antibody (ab8898) is specific for Histone H3 tri methyl Lysine 9. Shows slight cross-reactivity with tri methyl K27, which shares a similar epitope (please see Western blot image).” This antibody seems to cross react with other histone mark and at least H3K27me3 then to my view the best is either to remove the H3K9me3 data from the paper which will not impact the article since this is not a major point also, or to use a second antibody.

Another previous comment was:

Authors did not answer my comment about "enhancer-like classes" that "were enriched for active histone modifications such as H3K27/9ac, H3K4me1/3". It would be great that authors generate heat maps centered (i) on enhancer-like region, (ii) promoter, (iii) transcriptional unit, (IV) on polycomb and (V) on TE to show how the level of each histone modification looks. This will allow the reader to see if a correlation exists. Of course, the different regions should not overlap.

The response from authors is:

Thanks for your suggestion. Sorry we didn’t get your meaning last time. Now we have added this part of analysis as you suggested in Fig. S5a in the 2nd revision.

I am sorry but in figure S5A, none of the cluster is labeled. I do no know which cluster correspond to which chromatin state.

**Authors Response**

**Point-by-point responses to the reviewers’ comments:**

Reviewer #2:

In their revised version authors improved their manuscript. However, I still have some concerns about it.

One of my previous comments was:

1. “Authors in their response letter that "For dACRs, all genes within a 0.5-Mb region around a dACR were analyzed, and the ones that exhibited a significant correlation were selected as the targets (Fig. S4c)." This cannot be done this way. A regulatory element can be an enhancer but also a repressor sequence then if the authors assign all the time dACRs to a gene that exhibited a significant correlation within a 0.5-Mb region around it does not make sense for me. I totally disagree with this way of assigning a regulatory element to a gene. This is totally biased.”

The response from authors is:

“Thanks for your comments. We do agree that dACRs may function as enhancers or silencers. In this study, we only focused on enhancers rather than silencers, that is why we used a correlation strategy for positive regulatory dACRs discovering, which were named as reg-dACRs.

The correlation strategy for distal enhancer-genes assignment was used in many studies and has been validated by multiple experiments. The followings are brief citations and description of such analysis in these articles:

The paper cited by the authors cannot be used to prove that what they did is relevant.

(i) For the first paper cited authors wrote “The breadth and depth of this sequencing study enabled a robust association of ATAC-seq peaks with the genes that they are predicted to regulate. To do this, we implemented a strategy based on the correlation of ATAC-seq accessibility and gene expression across all samples (Fig. 5A, N=373 with matched RNA-seq and ATAC-seq). Because promoter capture Hi-C data suggested that >75% of three-dimensional (3D) promoter-based interactions occur within a 500-kbp distance (38), we restricted the length scale of this analysis to 500 kbp to avoid spurious predictions.” This means that (i) they used N=373 samples and also that they did not choose the distance randomly but “Because promoter capture Hi-C data suggested that >75% of three-dimensional (3D) promoter-based interactions occur within a 500-kbp distance”. The number of sample and the way they choose the distance makes this study and the revised version not comparable.

(ii) For the second one as authors highlight it “to predict enhancer target genes using the correlation between gene expression (as measured by RNA-seq) and H3K27ac enrichment at candidate enhancers within the same TAD” It is written within the same TAD meaning that yhey have interaction data to support this interaction. Again this is not what authors did in this paper.

(iii) For the third one they “ collected 117 ATAC-seq (assay for transposase-accessible chromatin with high-throughput sequencing) samples and 54 RNA-sequencing (RNA-seq) samples from hCSs and hSSs derived from 7 hiPS cell lines (tables S1 and S2)” and used the method presented in the first paper cited in (i). For the same reason it is not comparable.

(iv) For the last paper it is single cell data that where used and then of course it is also no comparable to what authors did in this manuscript.

To my view the best is tone down their conclusion and phrase it more cautiously or even better remove it since this is not a major point or conclusion in the paper.

*Reply: Thanks for your comments. We have removed this part analysis in Fig. 2 and Line 208-231 in the manuscript, as well as some related discussion.*

2. Another previous comment was:

“Authors wrote in their response about H3K9me3 "H3K9me3 is enriched in both TEs and expressed genes in plants, which function is not clear [22,23]" citing one paper using ChIP on ChIP and a review citing the paper that used the ChIP on ChIP. In fact almost no ChIP-seq are publicly available for H3K9me3 in plant and it is not clear at all if this mark is even present in plant.”

The response from authors is:

Thanks for the reviewer’s carefully checking, sorry for the meaningless citations we used in the last version of response letter. Indeed, both H3K9me2 and H3K9me3 ChIP- seq data have been generated in plant “Dynamic chromatin state profiling reveals regulatory roles of auxin and cytokinein in shoot regeneration” [25]. The antibody they used for H3K9me3 is “Abcam AB_306848(ab8898)”, the same as we used in this study. In the 2nd revision submitted currently, we have added all information regarding the antibodies in Table S19.

I have an issue with this antibody. As you can read it from the abcam web page (https://www.abcam.com/histone-h3-tri-methyl-k9-antibody-chip-grade-ab8898.html) . The supplier wrote about the specificity that “Histone H3 (tri methyl K9) antibody (ab8898) is specific for Histone H3 tri methyl Lysine 9. Shows slight cross-reactivity with tri methyl K27, which shares a similar epitope (please see Western blot image).” This antibody seems to cross react with other histone mark and at least H3K27me3 then to my view the best is either to remove the H3K9me3 data from the paper which will not impact the article since this is not a major point also, or to use a second antibody.

*Reply: Thanks for your comments. We have globally removed the H3K9me3 related data in the revised manuscript. Due to remove of H3K9me3, we have to rebuild the chromatin state and redo the related analysis in Fig. 3. Although there is a little bit difference, the main conclusions are not changed in the current revision.*

3. Another previous comment was:

Authors did not answer my comment about "enhancer-like classes" that "were enriched for active histone modifications such as H3K27/9ac, H3K4me1/3". It would be great that authors generate heat maps centered (i) on enhancer-like region, (ii) promoter, (iii) transcriptional unit, (IV) on polycomb and (V) on TE to show how the level of each histone modification looks. This will allow the reader to see if a correlation exists. Of course, the different regions should not overlap.

The response from authors is:

Thanks for your suggestion. Sorry we didn’t get your meaning last time. Now we have added this part of analysis as you suggested in Fig. S5a in the 2nd revision.

I am sorry but in figure S5A, none of the cluster is labeled. I do no know which cluster correspond to which chromatin state.

*Reply: Thanks for the reviewer’s careful checking, sorry for the mistake. We added the label in the revised figures. We think this analysis might be important, so the enriched histone modifications were placed in the main Fig. 3b, and the rest was placed in Fig. S4a of current revision.*

**Fourth round of review**

**Reviewer 2**

I am happy with the edits the authors have made to the document.
